# Reconstructing Late Pleistocene paleoclimate at the scale of human behavior: an example from the Neandertal occupation of La Ferrassie (France)

**DOI:** 10.1038/s41598-020-80777-1

**Published:** 2021-01-14

**Authors:** Sarah Pederzani, Vera Aldeias, Harold L. Dibble, Paul Goldberg, Jean-Jacques Hublin, Stéphane Madelaine, Shannon P. McPherron, Dennis Sandgathe, Teresa E. Steele, Alain Turq, Kate Britton

**Affiliations:** 1 Department of Human Evolution, Max-Planck-Institute for Evolutionary Anthropology, Leipzig, Germany; 2 Department of Archaeology, University of Aberdeen, Aberdeen, UK; 3 Interdisciplinary Center for Archaeology and Evolution of Human Behaviour, University of Algarve, Faro, Portugal; 4 Department of Anthropology, University of Pennsylvania, Philadelphia, USA; 5 Institute for Human Origins, Arizona State University, Tempe, USA; 6 CAS, SEALS, University of Wollongong, Wollongong, Australia; 7 Institute for Archaeological Sciences, Eberhard Karls University Tübingen, Tübingen, Germany; 8 Collège de France, Paris, France; 9 Musée National de Préhistoire, Les Eyzies-de-Tayac, France; 10 CNRS, University of Bordeaux, MCC, PACEA UMR 5199, Musée de Sauveterre-la-Lémance, Pessac, France; 11 Department of Archaeology, Simon Fraser University, Burnaby, Canada; 12 University of Pennsylvania Museum of Archaeology and Anthropology, Philadelphia, USA; 13 Department of Anthropology, University of California, Davis, Davis USA

**Keywords:** Palaeoclimate, Anthropology, Archaeology, Stable isotope analysis

## Abstract

Exploring the role of changing climates in human evolution is currently impeded by a scarcity of climatic information at the same temporal scale as the human behaviors documented in archaeological sites. This is mainly caused by high uncertainties in the chronometric dates used to correlate long-term climatic records with archaeological deposits. One solution is to generate climatic data directly from archaeological materials representing human behavior. Here we use oxygen isotope measurements of *Bos/Bison* tooth enamel to reconstruct summer and winter temperatures in the Late Pleistocene when Neandertals were using the site of La Ferrassie. Our results indicate that, despite the generally cold conditions of the broader period and despite direct evidence for cold features in certain sediments at the site, Neandertals used the site predominantly when climatic conditions were mild, similar to conditions in modern day France. We suggest that due to millennial scale climate variability, the periods of human activity and their climatic characteristics may not be representative of average conditions inferred from chronological correlations with long-term climatic records. These results highlight the importance of using direct routes, such as the high-resolution archives in tooth enamel from anthropogenically accumulated faunal assemblages, to establish climatic conditions at a human scale.

## Introduction

The study of Pleistocene human behavior is intimately connected to the environmental context in which human activity took place. During the Late Pleistocene, Europe was characterized by rapidly oscillating climatic shifts of dramatic magnitude^1,2^. Such changes would have posed significant challenges for all hominins, requiring great flexibility and resilience. However, how and to what degree hominin groups responded to harsh and rapidly changing climatic conditions in Late Pleistocene Europe is both debated and remains poorly understood, largely due to a lack of high resolution climate records that can be sufficiently linked to archaeological evidence. Specifically, while excellent local and global climate archives do exist^3–6^, the low precision in chronological dating methods for the archaeological record and for relevant climate archives often leads to a high uncertainty in correlating climatic data obtained from spatially removed archives with archaeological evidence of human activity. Thus, human–environment relationships are often established at time scales as broad as the marine isotope stage (MIS) level, as we have often been unable to incorporate into our interpretations intense climatic shifts that we know occurred on shorter time scales within each MIS.

This issue is especially acute for the record of Neandertal behavior, most of which occurs prior to the limit of radiocarbon dating. The rich archaeological record of southwest France and its role as a Late Pleistocene refugium^7^ make it a key region for investigating Neandertal behavior and climate resilience. However, due to the challenges outlined above, the regional climatic context for the last glaciation (MIS 5d to MIS 3; ca. 116—29 ka ago) prior to the Last Glacial Maximum remains poorly understood. This situation poses a substantial challenge, as ongoing research on Neandertal subsistence and hunting behavior^8,9^, fire use^10,11^, mobility, and lithic technology^12–14^ are heavily reliant on establishing or testing links with the local climatic context. Most such studies necessarily have to rely on using broad MIS scale climatic assignments. This means that archaeological records of human activities in some cases may have been produced during millennial-scale climate events under conditions that were fundamentally different from the broadly average climatic conditions of the corresponding MIS. Thus, we are missing data on human adaptations to short-term climatic variations within these larger climatic stages, and this in turn adds to the challenges of modeling changes in behavior across the Neandertal to *Homo sapiens* demographic transition that occurred roughly 50 to 40 thousand years ago^15,16^.

To address this issue of scale, we exploit the direct contextual connection between anthropogenically accumulated faunal assemblages and the archaeological record to reconstruct local seasonal climatic conditions during human activity. This contextual association between faunal assemblages and other archaeological material has previously been exploited in studies that infer environmental conditions from the species composition of such faunal assemblages in order to establish higher resolution environmental context for MIS 5 to 3 in southwest France^13,17^. Such approaches have demonstrated the usefulness of obtaining environmental or climatic context for human behavior in a manner that does not solely rely on correlation via chronometric ages. Here we build on these existing approaches by adding information from oxygen stable isotope analysis of faunal tooth enamel, a proxy of paleoclimate that is both quantitative and largely unaffected by anthropogenic biases related to prey choice or hunting behavior. Similar approaches have been previously employed for Late Pleistocene Europe^18–21^, but the implications of using anthropogenically accumulated archives for environmental archives have not always been specifically highlighted (but see^22^). We use oxygen isotope measurements ($\delta$^18^O) of sequentially sampled *Bos/Bison* tooth enamel to elucidate seasonal paleoclimate for La Ferrassie—a recently re-excavated and dated Pleistocene site in southwest France with evidence of Neandertal activity spanning MIS 5 to MIS 3^23–26^ (see Supplementary Text 1). We confirm that *Bos/Bison* $\delta$^18^O values are suitable as a local climatic proxy using strontium isotope analysis (^87^Sr/^86^Sr). Moreover, we complement diachronic changes in paleoclimate and paleoseasonality established by oxygen isotope analysis with environmental information on the underlying plant biome such as forest cover and isotopic niche of *Bos/Bison* from carbon and nitrogen stable isotope data ($\delta$^13^C and $\delta$^15^ N), generated from *Bos/Bison* bone collagen. We generate paleoclimate evidence for occupations at La Ferrassie dated to MIS 4 (Layer 2—associated with OSL dates of 62.5 $\pm \hspace{0.17em}$4.0 ka and 74.2 $\pm \hspace{0.17em}$4.6 ka; mean 68.4 $\pm \hspace{0.17em}$6 ka^27^) and MIS 3 (Layers 5A and 5B—^14^C dated to 47.5—44.2 ka cal. BP 95% probability ^28^ and OSL dated to 42.7 $\pm \hspace{0.17em}$4.7 ka^27^; however note an age inversion in the ^14^C dates between Layers 4 and 5, see Supplementary Text 1). In addition to the MIS 4 dates, Layer 2 is characterized by intense cold-climate features, such as cryoturbation and banded fabric, which were penecontemporaneous with its deposition (see Supplementary Text 2), suggesting formation during a cold stage, which contrasts with a mixed faunal assemblage containing substantial numbers of temperate adapted fauna (see Supplementary Text 3). We also use $\delta$^18^O to generate paleotemperature estimates to facilitate a comparison of our paleoclimate data with other paleoclimate proxies.

Due to the anthropogenic nature of the La Ferrassie faunal assemblage—as evidenced by low proportion of carnivore modifications on bone fragments (see Supplementary Text 4 and Supplementary Figure S6)—our stable isotope results of faunal remains are directly tied to hominin activity at the site. Thus, environmental reconstructions based on these remains will reflect the conditions during which the site was occupied by Neandertals. This enables us to generate a record that is directly relevant on a behavioral scale.

## Results

**Figure 1:**
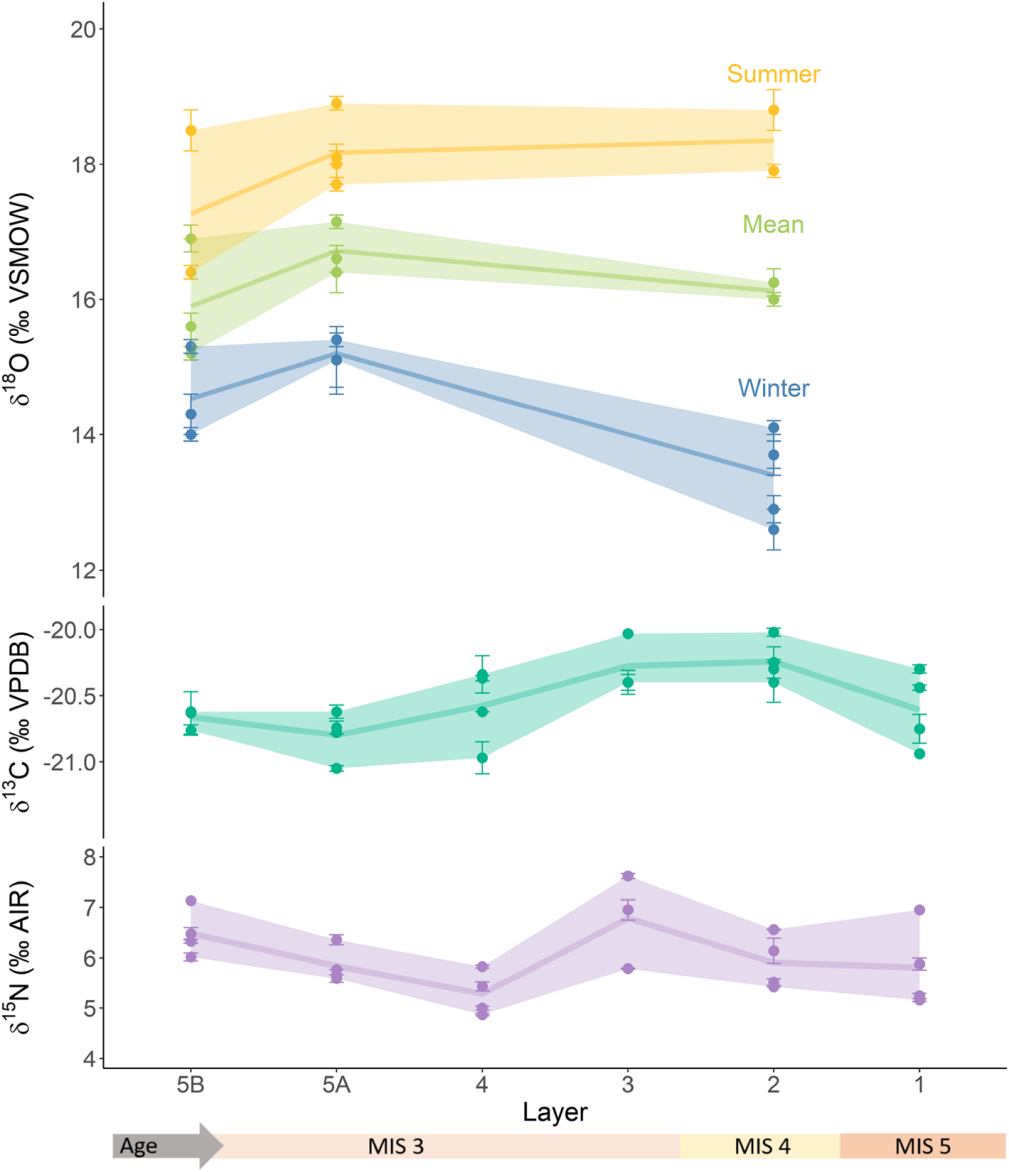
Correlated changes in $\delta$^18^O from tooth enamel (top) and $\delta$^13^C from bone collagen (middle) reflect minimal environmental changes across archaeological layers at La Ferrassie. Summer (yellow), mean annual (green) and winter (blue) $\delta$^18^O values are shown as individual points with a line connecting the layer means and a ribbon visualizing the maximal spread of the data. Summer peak and winter trough values are derived from sinusoidal $\delta$^18^O curves and are shown in Supplementary Figure S8. Mean annual points represent the average of peak and trough values, analogous to the calculation of modern mean annual temperatures. Summer temperatures and mean annual conditions stay stable between Layer 2 and 5. Winter temperatures show small differences between Layer 2 and Layers 5A and 5B, and are lowest and most variable in Layer 2. Diachronic in $\delta$^13^C and $\delta$^15^N values are congruent with small changes in winter $\delta$^18^O and support that each system robustly reflects an overall picture of relatively homogeneous temperature, soil isotope values and/or plant community structure with only subtle changes between layers.

All stable isotope measurements are listed in Supplementary Table S4 ($\delta$^18^O), Supplementary Table S5 (^87^Sr/^86^Sr) and Supplementary Table S6 ($\delta$^13^C and $\delta$^15^N). All teeth yielded a complete or partial sinusoidal $\delta$^18^O time series, with clearly identifiable winter troughs and/or summer peaks (Supplementary Figure S8), typical of annual cycles of temperature driven $\delta$^18^O fluctuations of environmental water recorded in faunal tooth enamel via drinking water and incorporation of oxygen into tooth enamel bioapatite (see Supplementary Text 5 and Supplementary Text 8). One individual did not display a clearly visible sinusoidal pattern (F7–14) and has been excluded from further analyses. All Sr isotope measurements of enamel, which are reflective of local bedrock ^87^Sr/^86^Sr, are homogeneous between different seasons, as well as across different individuals, with all values falling between 0.7100 and 0.7109 (mean = 0.7104 $\pm \hspace{0.17em}$0.0003 1 s.d.; see Supplementary Text 10 and Supplementary Figure S11). The enamel values also closely match the ^87^Sr/^86^Sr range of local bioavailable strontium as evidenced by modern local plants sampled close to the location of the site (mean = 0.7106 $\pm \hspace{0.17em}$0.0004 1 s.d.^29^; Supplementary Figure S11). They are within the range of values expected for the local limestone bedrock and nearby sandy riverbeds and are consistent with a restricted movement over a relatively small home range^30^ (see Supplementary Figure S12). Summer peak and winter trough $\delta$^18^O values were extracted by visual examination of sinusoidal $\delta$^18^O curves (see Supplementary Figure S8) and are summarized in Fig. 1. A clear pattern of $\delta$^18^O change is visible between layers, with more pronounced seasonality (winter − summer difference) in Layer 2 compared to Layers 5A and 5B, mostly related to changes in winter climatic conditions. Winter $\delta$^18^O values are significantly different between layers as determined by ANOVA (p = 0.006; normality of the dependent variable checked using a qqnorm plot; equality of variances checked using Levene’s test p = 0.46), with a significant change from Layer 2 to Layer 5A (as determined by a Tukey’s range test, p = 0.006) with an effect size of d = − 3.46.

Summer $\delta$^18^O values and mean annual conditions on the other hand stay more consistent through time (d < 2, see Supplementary Table S3  for an overview of all effect sizes). A slight cooling trend ($\delta$^18^O decrease) can be observed throughout Layer 5, from 5A to 5B in summer, winter and mean annual $\delta$^18^O values, but these changes are not statistically significant and show small effect sizes (d_summer_ = 1.14; d_winter_ = 1.34; d_mean annual_ = 1.19). In Layer 5B, we observe both teeth with higher $\delta$^18^O (I4-785) and slightly lower $\delta$^18^O (I4-616 and I4-792), but this does not appear correspond to a spatial trend within the layer stratigraphy (see Supplementary Figure S17).

Changes through time in $\delta$^18^O correlate well with small but concurrent shifts observed in plant ecosystem and feeding niche as evidenced by changes in $\delta$^13^C of *Bos/Bison* bone collagen (Fig. 1), with warmer layers showing lower $\delta$^13^C. For example, a good correlation can be observed between mean winter $\delta$^18^O and $\delta$^13^C bone collagen values within different layers (R^2^ = 0.76; Pearson correlation p = 0.0002; Supplementary Figure S13). Inter-level variations in *Bos/Bison* bone collagen $\delta$^15^N values are also observable and broadly map onto changes seen in the other systems, albeit with some possible temporal lag compared to $\delta$^18^O and $\delta$^13^C (Fig. 1; Supplementary Figure S14). This broad and consistent agreement between different isotopic systems—each reflecting different aspects of environmental change—highlights the robustness of the diachronic trends observed across the stratigraphic sequence.

**Figure 2:**
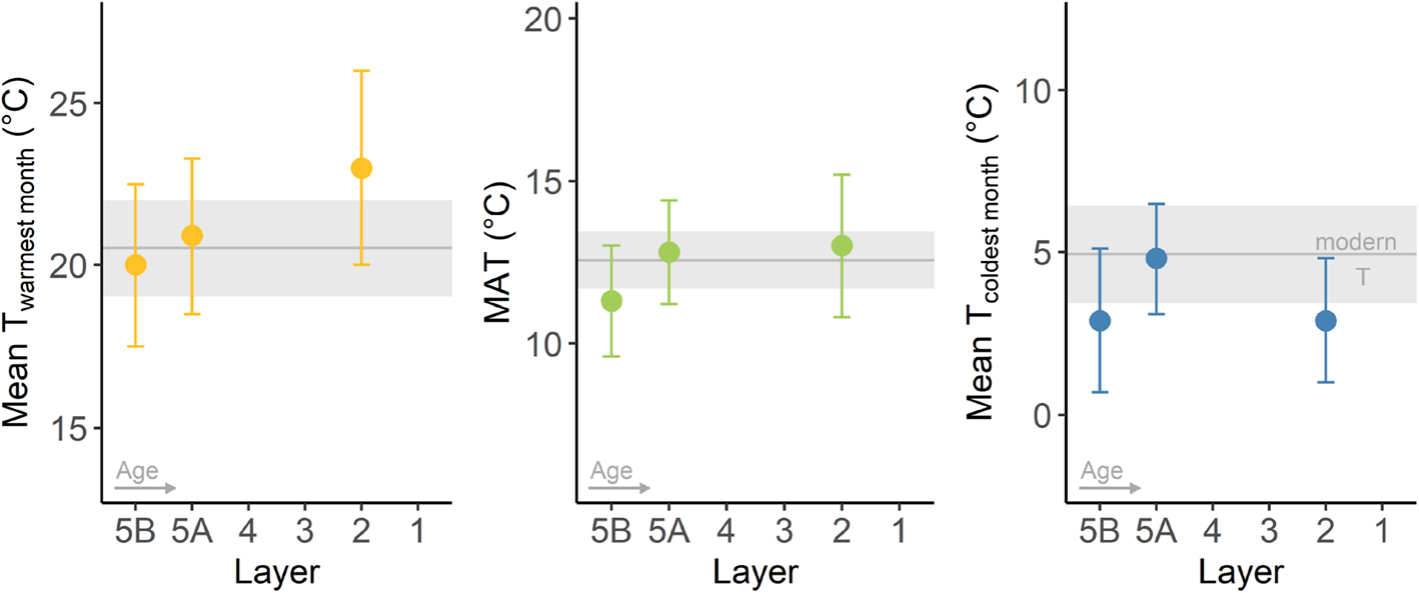
Reconstructed mean temperatures for the warmest month (left, yellow), mean annual temperature (middle, green) and mean temperatures of the coldest month (right, blue) and of the year by layer compared to modern day conditions in Gourdon (mean—grey line, standard deviation—grey ribbon, 1996–2017 average^31^). Error bars indicate compound error around the temperature reconstruction.

Summer and winter temperature estimates—derived from the linear relationships between enamel $\delta$^18^O and drinking water $\delta$^18^O and drinking water and $\delta$^18^O and air temperature respectively (see methods and model prerequisites in Supplementary Text 7 and 8) were generated to enable cross-comparison of our results with modern day conditions and other paleoclimate proxies. These estimates yield seasonal paleotemperatures close to modern day conditions in southwest France (Fig. 2). Temperatures of the warmest month mostly overlap with or exceed the modern July temperature of 20.5 °C^31^. The reconstructed range for mean temperature of the coldest month overlaps extensively with the modern January temperature of 4.9 °C, although reconstructed layer means are mostly slightly lower than today. This suggests an overall more pronounced temperature seasonality compared to the modern day, particularly for Layer 2. It should be noted that despite being mostly lower than modern conditions, all reconstructed winter temperatures are above 0 °C. Mean annual temperatures (MAT) reconstructed for Layers 2, 5A and 5B are very close to modern day mean annual temperatures in the region.

## Discussion and conclusion

Oxygen isotope delta values of *Bos/Bison* tooth enamel are homogeneous across layers, indicating a similarity in climate across these periods of site use (Fig. 1). Relatively high $\delta$^18^O values across all layers suggest that *Bos/Bison* lived in relatively mild conditions. Temperatures reconstructed to facilitate comparison with other proxies indicate very mild conditions for all three Upper Pleistocene layers at La Ferrassie with warm summers and cool winters, and no winters below freezing (Fig. 2).

Paleotemperature estimates are necessary to enable cross-proxy comparisons, but are subject to caveats related to model assumptions related to animal physiology, animal drinking behavior, atmospheric circulation and the isotopic composition of sea water among others and therefore carry some inherent uncertainty (see Supplementary Text 8). However, independent of precise temperature estimates, given the strong relationship between $\delta$^18^O of *Bos/Bison* tooth enamel with $\delta$^18^O of precipitation and in turn between $\delta$^18^O_precip_ and temperature (^32–34^, see Supplementary Text 8), the consistency in raw enamel $\delta$^18^O across MIS 3 and MIS 4 layers (Layer 5 and Layer 2) suggests a climatic similarity between these occupations, which is most likely related to a consistency in temperature between the different phases of site use. Other impacts that could distort a temperature signal in $\delta$^18^O could include diachronic changes in leaf water consumption^35–37^, changes in the isotopic composition of sea water^38–40^, a pronounced change in atmospheric circulation^39,41,42^, a diachronic change of *Bos/Bison* to a hydrologically distinct habitat^43^, or strong diachronic differences in aridity^35,44^. However, we argue that these effects are isotopically too small to substantially bias temperature effects on $\delta$^18^O in this case. Due to to the strong metabolic controls on *Bos/Bison* drinking requirements and surface water uptake *Bos/Bison* drinking behavior is very stable across modern day climates and ecosystems and a strong difference in the proportion of consumed water from food is unlikely even in the Pleistocene (see Supplementary Text 8). Isotopic data from fluid inclusions and other proxies for $\delta$^18^O_precip_ suggest that the effects on $\delta$^18^O_precip_ from changes in the isotopic composition of sea water between glacial and interglacial phases in MIS 3 and 4 are small (less than intra-layer variability seen here) and therefore are unlikely to have much impact on our result (see Supplementary Text 8). The impact of variability in atmospheric circulation on the $\delta$^18^O_precip_-temperature relationship and how this is numerically represented in the uncertainty estimate of paleotemperature reconstructions is discussed in depth in Supplementary Text 8, but previous work indicates that this relationship remains relatively stable into the Late Pleistocene^45^. A difference in *Bos/Bison* habitat between layers could have implications for oxygen isotopic patterns, if habitats contain isotopically divergent major sources of drinking water. This has for instance been proposed for MIS 8–7 fauna at the site of Payre, southeast France, where animals in valley habitats could access river water with low $\delta$^18^O while animals in plateau habitats could use precipitation derived drinking water with comparatively higher $\delta$^18^O^43,46^. Strontium isotope ratios of tooth enamel show that *Bos/Bison* ranged over small geographical areas close to the site, and most likely did not move as far as the higher plateaus to the northeast of the site (Supplementary Text 10 and Supplementary Figures S11 and S12). This makes them generally a faithful proxy for local climatic conditions, but leaves open the possibility of ranging across valleys and low elevation limestone plateaus in the vicinity of the site. However, in contrast to the hydrotopgraphical situation at Payre, we estimate the isotopic differences in our specific site setting to be no more than approximately 1‰—less than inter-individual variability within one layer (see Supplementary Text 8 for details). Additionally, the pronounced seasonal amplitude seen in tooth enamel $\delta$^18^O time series (Supplementary Figure S8) demonstrates that *Bos/Bison* did not consume large amounts of water from seasonally buffered water sources that isotopically diverge from precipitation such as deep groundwater or water from large lakes.

A climatically and specifically temperature driven diachronic similarity in $\delta$^18^O is supported by congruous patterns in carbon and nitrogen stable isotope values of *Bos/Bison* bone collagen (Fig. 1), albeit with some temporal offsets between $\delta$^15^N compared to $\delta$^13^C and $\delta$^18^O, similar to off-set patterns observed at other late Pleistocene sites^47,48^. The negative correlation in the subtle diachronic changes in $\delta$^18^O and $\delta$^13^C additionally further strengthen the interpretation that $\delta$^18^O is predominantly temperature driven with negligible influence of aridity, as low $\delta$^18^O phases coincide with drier or more open environments characterized by higher $\delta$^13^C values in the plant baseline^20,49–51^. If strong aridity effects were acting on $\delta$^18^O, a positive correlation between the tracers would be expected. An absence of strong aridity differences between Layers 5 and 2 is also supported by their similarity in $\delta$^15^N, which has been found to be higher in more arid phases of MIS 3 in southwest France^20,52^.

**Figure 3:**
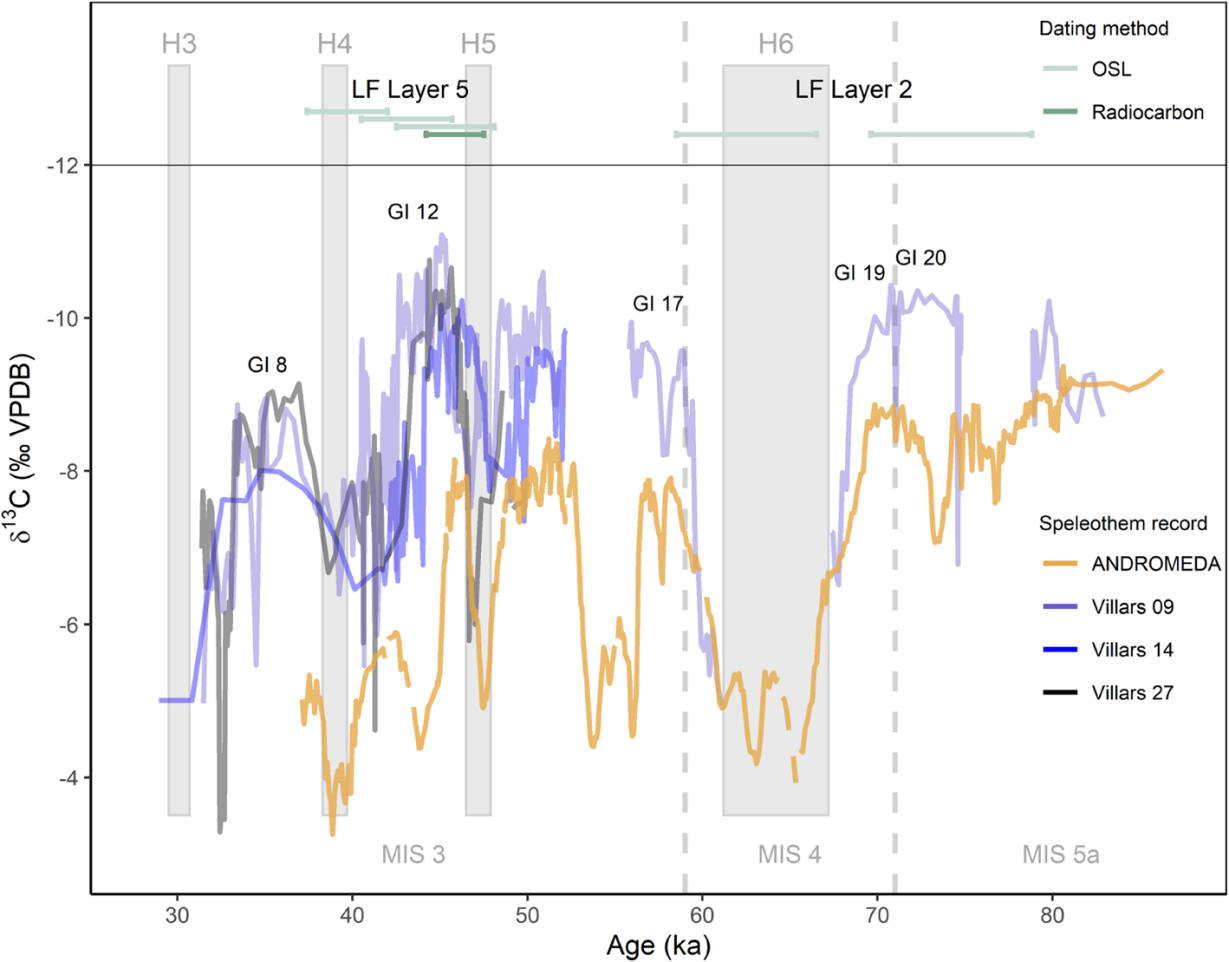
Speleothem records (bottom) from Villars cave^3^ and Ejulve cave (ANDROMEDA speleothem; 4) show substantial millennial scale climate variability in both MIS 4 and MIS 3. Given the age ranges (top)^27^ and the warm temperatures of site occupation obtained for La Ferrassie Layer 2 suggest that site activity in MIS 4/late 5a may be attributed to one or several MIS 4 ephemeral warm phases. La Ferrassie Layer 5 likely falls into a warm episode in MIS 3 possibly around Greenland Interstadial 12 (but note an age inversion in the ^14^C dates of Layer 5 and Layer 4 as discussed in Supplementary Text 1).

In the resulting framework of interpreting $\delta$^18^O as being predominantly driven by temperature, our results mean that the deposition of faunal remains and, therefore, activity at the site in these layers took place when local conditions were comparatively warm, similar to modern day conditions. Oxygen stable isotope values show that climatic conditions were similar between Layer 2 and Layers 5A and 5B with only small variations in winter climatic conditions (Fig. 1), despite dates falling into the predominantly cold MIS 4 and the warmer MIS 3 respectively (Fig. 3, Supplementary Text 1). Consequently, environmental conditions were relatively similar across different periods of Neandertal activity at La Ferrassie, with possible small changes in plant ecosystem, a trend also seen in pollen records of this time period^53^.

The similarity in stable isotope systems across different episodes of activity at the site stands in contrast to the overall climatic conditions reconstructed for the marine isotopes stages (4 and 3) represented in Layer 2 and 5, respectively, based on their dates. This is particularly the case for Layer 2, which is attributed to MIS 4 and shows pronounced cold temperature features in the sediments and this mismatch between isotopic and geological climate indicators does not appear to be the result of reworking processes. Layer 2 is comprised primarily of frost-derived breakdown products of limestone (bedrock or very large blocks of roof collapse). Only at the very base of the layer do we observe quartz grains inherited from Layer 1. Therefore, Layer 2 represents soliflucted material derived mainly from cold-induced breakdown of this limestone source to the west of the site and little from a previously existing sedimentary source, if at all. Indications of cold climatic conditions during layer formation are found in both the Layer portions derived from cryoclastic breakdown of limestone beadrock (close to the wall) and portions more affected by the solifluction cone (further away from the wall). The even distribution of reindeer bones and bones identified as large cervid or reindeer throughout the layer as well as a lack of oxygen isotopic difference between *Bos/Bison* from different portions of the Layer (see Supplementary Text 12) also suggests that the *Bos/Bison* remains found in Layer 2 were deposited during the time of Layer 2 sediment formation and are unlikely to be derived from reworked material that was deposited at an earlier time during a warmer climate. We therefore believe the two climatic proxies to broadly represent the same time period, but at different scales of time averaging.

MIS 4 is generally characterized as a severe cold stage with significant ice expansion^54,55^ and is evidenced as such in western Europe and southwest France in particular in speleothem records^3,4^ (Fig. 3) and sea surface temperatures in the French Atlantic^5^. The development of a cold steppe tundra after ca 70–65 ka is evidenced in a number of French and Spanish pollen archives^5,6,56,57^ and rodent assemblages from southwest France (Les Pradelles Layers 2a and 2b, Chez-Pinaud Jonzac Layers 24–10^17^). These records match reindeer-dominated macrofaunal assemblages present at a number of MIS 4 archaeological sites (e.g. Pech de l’Azé IV Layer 4C, Roc de Marsal Layers 4 and 5, Les Pradelles Layers 11-9, Chez-Pinaud Jonzac Layers 24-10, La Quina Layer 8)^17^. Pollen and beetle (Coleoptera) analyses yield mean annual temperature (MAT) estimates for MIS 4 between − 4 and 6 °C and summer temperatures of 10 to 18 °C for eastern France^58,59^ while rodent $\delta$^18^O_p_ values from the site of Les Pradelles suggest summer temperatures of ca. 16–17 °C in western France during this time^60^.

We propose that the inconsistencies between the isotopic data and the marine isotope stage reconstructions and sedimentary evidence can be resolved by considering the presence of millennial scale warm episodes within each MIS, which are captured in the short-term records of faunal stable isotope data from punctuated archaeological occupations. Such short-term oscillations are not visible in the time averaged climatic records from sediments. Speleothem, pollen, and sea surface temperature records all show substantial millennial scale climatic variability in MIS 4 and late MIS 5a. They include several short warm phases during the overall cold conditions of MIS 4^3–6,56^ (Fig. 3), which could be reflected in the short-term climatic record of tooth enamel from animals butchered at archaeological sites that are the product of punctuated human activity. Similarly, the dates for Layer 5A and 5B^27,28^ and the warm climatic reconstruction from our stable isotope data indicate that these layers are most likely associated with a warm phase in MIS 3, likely around Greenland Interstadial (GI) 12 (but note the ^14^C age inversion between Layer 4 and Layer 4 as discussed in Supplementary Text 1) which is documented in speleothems and sea surface temperature records^3–5^. Our results for this phase match well with these other temperature records as well as MIS simulations^20,61^.

So whereas Layer 2 shows unequivocal sedimentary evidence for sub-zero temperatures at least during portions of the layer formation period (see Supplementary Text 2) representative of much of MIS 4, the climatic variability on smaller time scales including brief warm episodes known already for MIS 4 are not resolved in this broadly time averaged sedimentary record. They are captured in short term, sub-annually resolved stable isotope data from faunal remains, which provide a ‘snap-shot’ record of climatic conditions during the lifetimes of prey animals before butchery and deposition at the site by Neandertal groups. In this way, as an anthropogenic archive, stable isotope values of faunal remains show climatic conditions specifically during human occupation of an archaeological site. Such phases of site use may be punctuated in nature and can potentially fall into time periods with climatic conditions that are not necessarily representative of a longer time climatic average obtained from more time averaged climatic proxies. Consequently, correlations between environmental change and Paleolithic settlement patterns can differ substantially depending on the time resolution and geological or anthropogenic nature of the climatic proxy that is used, as they operate on fundamentally different time scales.

This observation may help explain why exploring environmental influences on Paleolithic settlement patterns, behavior, or material culture in southwest France has so far been challenging, with different studies and sites yielding contradictory results^8,11,12,14,17^. While factors such as dating uncertainty, heterogeneity in excavation methodology and assemblage curation are important, some disagreements may also stem from the use of broadly time-averaged geologically accumulated climate archives. In the case of La Ferrassie, we propose that the difference between proxies (geological data and faunal stable isotope data) suggests that Neandertal activity at the site was largely restricted to brief warm intervals of the generally cold MIS 4/late MIS 5a interval, indicating a particular site use pattern tied to mild climatic conditions. While surprising, alternative explanations rooted in methodological issues or characteristics of the site formation cannot explain our results. As discussed above, we can to a large extent exclude that the isotopic patterns seen here are the result of reworking of material from an earlier warm phase. Similarly, an erroneous age assignment of Layer 2 appears unlikely. OSL and Radiocarbon ages are in good agreement for the upper sections of the sequence, and single grain OSL analysis for the older of the two Layer 2 OSL samples indicates that this sample is well bleached^27^. We therefore have no indication for an age overestimation due to insufficient bleaching for Layer 2, supporting the MIS 4 assignment.

Support for this punctuated pattern of site use during similar environmental conditions can also be found in the homogeneity of archaeological assemblages in terms of exploitation of faunal resources and lithic technology (see Supplementary Texts 1, 3 and 4). Blank production is dominated by Levallois technology throughout the MIS 5—3 sequence and stone tools contain variable proportions of scrapers between 30–50% (Supplementary Text 1). Similar assemblages are commonly found throughout MIS 5, 4 and sometimes 3 in southwest France with a wide variety of associated faunal spectra, although longer sequences that cover several marine isotope stages often also exhibit layers with stone tool assemblages described as Quina Mousterian^12,14,17^, which is absent at La Ferrassie. The proportion of different prey taxa, shows little change across the sequence (Supplementary Figure S5), and we also observe a relatively stable level of processing intensity, as indicated by impact marks from marrow extraction or surface modifications generally (i.e. cut marks, scraping, anvil-marks, use as retoucher; Supplementary Figure S6). Such archaeological data imperfectly capture underlying complex behaviors and are each driven by multiple interconnected factors. However, we suggest that a site-use pattern restricted to warm phases meant that La Ferrassie was particularly suited or attractive for warm phase occupation and may have provided a favorable environment for a specific suite of behaviors related to subsistence, site use, or material culture in Neandertal technology.

A similar explanation has been suggested for the lower layers at Roc de Marsal where luminescence dates place the deposits in MIS 4 but with a more temperate faunal assemblage suggesting a wooded environment^62^. Meanwhile, at other sits in the region, such as Pech de l’Azé IV, the upper layers of Roc de Marsal, Les Pradelles, Chez-Pinaud Jonzac and La Quina,^17^ the presence of reindeer dominated faunal assemblages seems to suggest that Neandertal groups were nonetheless using the region more broadly during colder conditions. However, independent isotopic evidence on the climatic conditions from periods of human occupation at these other site is missing. More studies generating climatic evidence directly from archaeological materials—for example using stable isotope analysis of anthropogenic faunal assemblages—are needed to further explore the relationship between patterns of activity between different sites in the same region and potential factors that may drive such patterns.

Thus, isotopic analyses of anthropogenic faunal assemblages at La Ferrassie demonstrate that climatic conditions during human activity at Paleolithic archaeological sites can substantially differ from average climatic conditions of the broader time interval bracketed by chronometric dates of archaeological layers. We propose that this mismatch is a consequence of pronounced (sub-)millennial scale climate complexity in the Late Pleistocene record that remain largely invisible in more strongly time average geological archives. Our results highlight the necessity for obtaining direct evidence for climatic conditions of human site use in order to establish robust insights into human behavior and climatic resilience on short time scales beyond the marine isotope stage scale, a key aspect for elucidating the relationship between climatic shifts and human subsistence, and mobility.

## Methods

Tooth enamel of *Bos/Bison* teeth from three archaeological layers (Layer 2, 5A and 5B; see Supplementary Table S2) was sequentially sampled (n_samples_ = 178, n_teeth_ = 13) to generate paleoclimatic data from $\delta$^18^O of bioapatite phosphate. A mixture of *Bos/Bison* third molars, second molars and 4th premolars were chosen from layers where several suitable teeth were available. To ensure the applicability of the $\delta$^18^O data for paleoclimate reconstruction, we use strontium radiogenic isotope data from the same individuals (subset of n_samples_ = 15, n_teeth_ = 7) to exclude long distance migratory behavior in the *Bos/Bison* used in this study. Due to the low number of suitable teeth available from the site, our sampling strategy was focused on sampling a larger number of teeth and in some cases it cannot be excluded that the same individual was sampled twice (see Supplementary Text 5). Using information on tooth position, tooth wear and similarity of stable isotope profiles, we conclude that data from a minimum of 12 distinct individuals are represented by the 13 teeth used in our study (see Supplementary Text 5 and see Supplementary Tables S7–S9). We complement diachronic changes in paleoclimate and paleoseasonality with environmental information on the underlying plant biome such as forest cover and isotopic niche of *Bos/Bison* from carbon and nitrogen stable isotope data, generated from *Bos/Bison* bone collagen (n = 28) covering the complete Middle Paleolithic sequence of the site (Layers 1 through 5B). We discuss the relationship and contextual connection between bone and tooth samples in Supplementary Text 12.

To obtain tooth enamel samples for oxygen and strontium isotope analysis sequential samples were drilled in small strips (ca. 8 × 1.5 × 0.7 mm) perpendicular to the tooth growth axis using a diamond tipped drill bit. Bioapatite phosphate was extracted from tooth enamel powders and converted to silver phosphate using a HF digestion and rapid precipitation methodology without prior pretreatment (see Supplementary Text 5). Oxygen isotope ratios of Ag_3_PO_4_ were generally analyzed in triplicate, using a high temperature elemental analyzer (TC/EA) coupled to a Delta V mass spectrometer via a Conflo IV interface (Thermo Fisher Scientific, Bremen, Germany; see Supplementary Text 5). Oxygen isotope delta values were two-point scale normalized to the VSMOW scale using matrix matched standards calibrated to international reference materials and scale normalization was checked using three separate quality control standards including NIST SRM 120c (previously NBS 120c), which gave values of 21.8 (n = 43) $\pm \hspace{0.17em}$0.5‰ (see full details in Supplementary Text 5). Reproducibility of replicate measurements of each sample was 0.2‰ on average. Raw $\delta$^18^O seasonal curves were corrected for damping of the amplitude from time-averaging due to enamel mineralization times and sampling geometry prior to conversion into temperature values using methods in^41^ (see Supplementary Texts 7 and 8). Bone collagen was extracted using a modified Longin method including an ultrafiltration step and then analyzed for carbon and nitrogen stable isotopes using a Flash EA 2000 Organic Elemental Analyzer coupled to a Delta XP ratio mass spectrometer via a Conflo III interface (Thermo Fisher Scientific, Bremen, Germany; see details in Supplementary Text 9). Sample preparation for strontium isotope analysis was conducted using methods following^63^ and analyzed using a Neptune Multi-Collector Inductively Coupled Plasma Mass Spectrometer (MC-ICPMS, Thermo Fisher Scientific, Bremen, Germany; see Supplementary Text 10). This article, including code for all data analyses, was written in R (version 3.6.2) and the manuscript rendered using RMarkdown. All raw data as well as the RMarkdown script to reproduce the article and its analyses are available at https://osf.io/sfnb8. Packages and version details can be found in Supplementary Text 13.

## Supplementary Information

Supplementary Information.

## References

[1] Dansgaard W (1982). A new Greenland deep ice core. Science.

[2] Stuiver M, Grootes PM (2000). GISP2 oxygen isotope ratios. Quatern. Res..

[3] Genty D (2010). Isotopic characterization of rapid climatic events during OIS3 and OIS4 in Villars Cave stalagmites (SW-France) and correlation with Atlantic and Mediterranean pollen records. Quatern. Sci. Rev..

[4] Pérez-Mejías C (2019). Orbital-to-millennial scale climate variability during Marine Isotope Stages 5 to 3 in northeast Iberia. Quatern. Sci. Rev..

[5] Sánchez Goñi MF, Bard E, Landais A, Rossignol L, D’Errico F (2013). Air–sea temperature decoupling in western Europe during the last interglacial–glacial transition. Nat. Geosci..

[6] Fletcher WJ (2010). Millennial-scale variability during the last glacial in vegetation records from Europe. Quatern. Sci. Rev..

[7] Hofreiter M, Stewart J (2009). Ecological change, range fluctuations and population dynamics during the pleistocene. Curr. Biol..

[8] Hodgkins J (2016). Climate-mediated shifts in Neandertal subsistence behaviors at Pech de l’Azé IV and Roc de Marsal (Dordogne Valley, France). J. Hum. Evol..

[9] Rendu W (2019). Subsistence strategy changes during the Middle to Upper Paleolithic transition reveals specific adaptations of human populations to their environment. Sci. Rep..

[10] Dibble HL (2017). How did hominins adapt to ice age Europe without fire?. Curr. Anthropol..

[11] Sorensen AC (2017). On the relationship between climate and Neandertal fire use during the Last Glacial in south-west France. Quatern. Int..

[12] Delagnes A, Rendu W (2011). Shifts in Neandertal mobility, technology and subsistence strategies in western France. J. Archaeol. Sci..

[13] Discamps E, Jaubert J, Bachellerie F (2011). Human choices and environmental constraints: Deciphering the variability of large game procurement from Mousterian to Aurignacian times (MIS 5–3) in southwestern France. Quatern. Sci. Rev..

[14] Faivre J-P (2014). The contribution of lithic production systems to the interpretation of Mousterian industrial variability in south-western France: The example of Combe-Grenal (Dordogne, France). Quatern. Int..

[15] Hublin JJ (2015). The modern human colonization of western Eurasia: When and where?. Quatern. Sci. Rev..

[16] Higham T (2014). The timing and spatiotemporal patterning of Neanderthal disappearance. Nature.

[17] Discamps E, Royer A (2016). Reconstructing palaeoenvironmental conditions faced by Mousterian hunters during MIS 5 to 3 in southwestern France: A multi-scale approach using data from large and small mammal communities. Quatern. Int..

[18] Bernard A (2009). Pleistocene seasonal temperature variations recorded in the δ 18O of Bison priscus teeth. Earth Planet. Sci. Lett..

[19] Fabre M (2011). Late Pleistocene climatic change in the French Jura (Gigny) recorded in the δ18O of phosphate from ungulate tooth enamel. Quatern. Res..

[20] Richards MP (2017). Temporal variations in Equus tooth isotope values (C, N, O) from the Middle Paleolithic site of Combe Grenal, France (ca. 150,000 to 50,000 BP). J. Archaeol. Sci. Rep..

[21] Scherler L, Tütken T, Becker D (2014). Carbon and oxygen stable isotope compositions of late Pleistocene mammal teeth from dolines of Ajoie (Northwestern Switzerland). Quatern. Res. (United States).

[22] Skrzypek G, Winiewski A, Grierson PF (2011). How cold was it for Neanderthals moving to Central Europe during warm phases of the last glaciation?. Quatern. Sci. Rev..

[23] Capitan L, Peyrony D (1921). Découverte d’un sixième squelette moustérien à La Ferrassie (Dordogne). Rev. Anthropol..

[24] Peyrony, D. La Ferrassie. Moustérien, Périgordien, Aurignacien. Préhistoire III. *Préhistoire* (1934)

[25] Turq, A. *et al.* La Ferrassie: Rapport d’opération pour l’année 2012 (2012).

[26] Delporte, H. & Delibrias, G. *Le grand abri de la Ferrassie: fouilles 1968–1973*. (Ed. du Laboratoire de paléontologie humaine et de préhistoire, 1984).

[27] Guérin G (2015). A multi-method luminescence dating of the Palaeolithic sequence of La Ferrassie based on new excavations adjacent to the La Ferrassie 1 and 2 skeletons. J. Archaeol. Sci..

[28] Talamo S (2020). The new 14C chronology for the Palaeolithic site of La Ferrassie, France: The disappearance of Neanderthals and the arrival of Homo sapiens in France. J. Quatern. Sci..

[29] Britton, K. *et al.* Sampling plants and malacofauna in 87Sr/86Sr bioavailability studies: Implications for isoscape mapping and reconstructing of past mobility patterns. Front. Ecol. Evol. **8, **579473 (2020).

[30] Willmes M (2018). Mapping of bioavailable strontium isotope ratios in France for archaeological provenance studies. Appl. Geochem..

[31] Deutscher Wetterdienst. Monthly mean air temperature of Gourdon, Dépt. Lot; Aquitaine/France (1996–2017) (2020).

[32] Hoppe KA (2006). Correlation between the oxygen isotope ratio of North American bison teeth and local waters: Implication for paleoclimatic reconstructions. Earth Planet. Sci. Lett..

[33] D’Angela D, Longinelli A (1990). Oxygen isotopes in living mammal’s bone phosphate: Further results. Chem. Geol. Isot. Geosci. Sect..

[34] Rozanski K, Araguás-Araguás L, Gonfiantini R (1992). Relation between long-term trends of oxygen-18 isotope composition of precipitation and source. Sci. New Ser..

[35] Levin NE, Cerling TE, Passey BH, Harris JM, Ehleringer JR (2006). A stable isotope aridity index for terrestrial environments. Proc. Natl. Acad. Sci. U.S.A..

[36] Kohn MJ, Schoeninger MJ, Valley JW (1996). Herbivore tooth oxygen isotope compositions: Effects of diet and physiology. Geochim. Cosmochim. Acta.

[37] Bocherens H, Koch PL, Mariotti A, Geraads D, Jaeger J-J (1996). Isotopic biogeochemistry (^13^C, ^18^O) of mammalian enamel from african pleistocene hominid sites. Palaios.

[38] Shackleton N (1967). Oxygen isotope analyses and Pleistocene temperatures re-assessed. Nature.

[39] Dansgaard W (1964). Stable isotopes in precipitation. Tellus.

[40] Gat JR (2010). Isotope Hydrology: A Study of the Water Cycle.

[41] Pryor AJE, Stevens RE, O’Connell TC, Lister JR (2014). Quantification and propagation of errors when converting vertebrate biomineral oxygen isotope data to temperature for palaeoclimate reconstruction. Palaeogeogr. Palaeoclimatol. Palaeoecol..

[42] Rozanski K, Araguás-Araguás L, Gonfiantini R (1993). Isotopic patterns in modern global precipitation. Clim. Change Cont. Isot. Rec..

[43] Bocherens H (2016). Direct isotopic evidence for subsistence variability in Middle Pleistocene Neanderthals (Payre, southeastern France). Quatern. Sci. Rev..

[44] Ingraham, N. L., Caldwell, E. A. & Verhagen, B. T. Arid Catchments. in *Isotope tracers in catchment hydrology*, 435–465 (Elsevier, 1998). 10.1016/B978-0-444-81546-0.50020-3.

[45] Tütken T, Furrer H, Walter Vennemann T (2007). Stable isotope compositions of mammoth teeth from Niederweningen, Switzerland: Implications for the Late Pleistocene climate, environment, and diet. Quatern. Int..

[46] Ecker M (2013). Middle pleistocene ecology and neanderthal subsistence: Insights from stable isotope analyses in Payre (Ardèche, southeastern France). J. Hum. Evol..

[47] Stevens RE (2008). Nitrogen isotope analyses of reindeer (Rangifer tarandus), 45,000 BP to 9,000 BP: Palaeoenvironmental reconstructions. Palaeogeogr. Palaeoclimatol. Palaeoecol..

[48] Stevens RE, Hermoso-Buxán XL, Marín-Arroyo AB, González-Morales MR, Straus LG (2014). Investigation of Late Pleistocene and Early Holocene palaeoenvironmental change at El Mirón cave (Cantabria, Spain): Insights from carbon and nitrogen isotope analyses of red deer. Palaeogeogr. Palaeoclimatol. Palaeoecol..

[49] Drucker DG, Bridault A, Hobson KA, Szuma E, Bocherens H (2008). Can carbon-13 in large herbivores reflect the canopy effect in temperate and boreal ecosystems? Evidence from modern and ancient ungulates. Palaeogeogr. Palaeoclimatol. Palaeoecol..

[50] Diefendorf AF, Mueller KE, Wing SL, Koch PL, Freeman KH (2010). Global patterns in leaf ^13^C discrimination and implications for studies of past and future climate. Proc. Natl. Acad. Sci..

[51] Feranec RS, García N, Díez JC, Arsuaga JL (2010). Understanding the ecology of mammalian carnivorans and herbivores from Valdegoba cave (Burgos, northern Spain) through stable isotope analysis. Palaeogeogr. Palaeoclimatol. Palaeoecol..

[52] Bocherens H, Drucker DG, Madelaine S (2014). Evidence for a 15N positive excursion in terrestrial foodwebs at the Middle to Upper Palaeolithic transition in south-western France: Implications for early modern human palaeodiet and palaeoenvironment. J. Hum. Evol..

[53] Sanchez Goni MF (2008). Contrasting impacts of Dansgaard-Oeschger events over a western European latitudinal transect modulated by orbital parameters. Quatern. Sci. Rev..

[54] Ruddiman WF, McIntyre A (1981). Oceanic mechanisms for amplification of the 23,000-year ice-volume cycle. Science.

[55] Kindler P (2014). Temperature reconstruction from 10 to 120 kyr b2k from the NGRIP ice core. Clim. Past.

[56] Guiter F (2003). The last climatic cycles in Western Europe: A comparison between long continuous lacustrine sequences from France and other terrestrial records. Quatern. Int..

[57] de Beaulieu J-L, Reille M (1992). The last climatic cycle at La Grande Pile (Vosges, France) a new pollen profile. Quatern. Sci. Rev..

[58] Van Andel TH, Tzedakis PC (1996). Palaeolithic landscapes of Europe and environs, 150,000–25,000 years ago: An overview. Quatern. Sci. Rev..

[59] Ponel P (1995). Rissian, Eemian and Würmian Coleoptera assemblages from La Grande Pile (Vosges, France). Palaeogeogr. Palaeoclimatol. Palaeoecol..

[60] Royer A (2013). Late Pleistocene (MIS 3–4) climate inferred from micromammal communities and δ 18O of rodents from Les Pradelles, France. Quatern. Res. (United States).

[61] Barron, E., Andel, T. H. van & Pollard, D. Glacial environments II: Reconstructing the climate of Europe in the last glaciation. *Neanderthals and modern humans in the European landscape during the last glaciation* 57–78 (2003).

[62] Guérin G (2012). Multi-method (TL and OSL), multi-material (quartz and flint) dating of the Mousterian site of Roc de Marsal (Dordogne, France): Correlating Neanderthal occupations with the climatic variability of MIS 5-3. J. Archaeol. Sci..

[63] Copeland SR (2008). Strontium isotope ratios (87Sr/86Sr) of tooth enamel: A comparison of solution and laser ablation multicollector inductively coupled plasma mass spectrometry methods. Rapid Commun. Mass Spectrom..

