# Supplementary material for: Reconstructing Late Pleistocene paleoclimate at the scale of human behavior: an example from the Neandertal occupation of La Ferrassie (France)
Source: Sci Rep. 2021 Jan 14;11:1419. doi: 10.1038/s41598-020-80777-1 (PMC7809458; doi:10.1038/s41598-020-80777-1)
Supplement: Supplementary file 1 — Supplementary Information. [file 41598_2020_80777_MOESM1_ESM.pdf]

Supplementary Information for

Reconstructing Late Pleistocene paleoclimate at the scale of human behavior: An example from the Neandertal occupation of La Ferrassie (France)

Sarah Pederzani, Vera Aldeias, Harold L. Dibble, Paul Goldberg, Jean-Jacques Hublin, Stéphane Madelaine, Shannon P. McPherron, Dennis Sandgathe, Teresa E. Steele, Alain Turq, Kate Britton

Corresponding author: Sarah Pederzani

Email address: sarah_pederzani@eva.mpg.de

This PDF file includes:

Supplementary text (Supplementary Information 1-15)

Supplementary Figures S1 to S19

Supplementary Tables S1 to S9

Captions for Supplementary Figures S1 to S19 and Supplementary Tables S1 to S9

SI References

Other supplementary materials for this manuscript include the following:

The code and data used to produce the main text and supplementary information and underlying analyses are available at https://osf.io/sfnb8/?view_only=622493c0f28b48c99eece196a5fe0b7b.

1. Site background

The site of La Ferrassie is situated in the Dordogne region of southwest France, close to the commune of Le Bugue. La Ferrassie comprises a complex of several sites: the 'Grand Abri'

(large rock shelter), a smaller rock shelter and a small cave, (Upper Cave). In this study we focus on the Grand Abri (hereafter La Ferrassie) which was excavated by Capitan and Peyrony and later by Delporte.[1–4] Our study is based on samples from the recent excavations by Turq and colleagues.[5] During excavation, from 2010 to 2015, nine major stratigraphic units were defined, belonging to the Middle and Upper Paleolithic including the Middle to Upper Paleolithic transition period (Supplementary Figure S1 A); Layer 9 was spatially restricted and is not depicted in the stratigraphic image below). The chronology for the site was established using a combination of OSL[6,7] and radiocarbon dates[6,8] and fits in a time frame from MIS 5 to the beginning of MIS 2 (Supplementary Figure S1 A). Layer 1, at the base of the sequence, is placed into MIS 5b/5a based on two OSL dates of 86.7 $\pm$ 7.2 ka and 91.5 $\pm$ 8.8 ka[6] (Supplementary Figure S1 A). Two OSL dates of 62.5 $\pm$ 4.0 ka and 74.2 $\pm$ 4.6 ka (mean 68.4 $\pm$ 6 ka) place Layer 2 predominantly in MIS 4, with some possibility of a late MIS 5a date.[6] Due to the presence of pronounced cold climate features in the Layer 2 sediments (see Supplementary Text 2), the layer most likely belongs to MIS 4.[6] Layers 3 through 5 then cover early to mid MIS 3. The most recent dating effort of the site yielded layer boundary dates for these layers of 52.4 - 46.2 ka (cal. BP 95% probability), 49.1 - 45.8 ka (cal. BP 95% probability) and 47.5 - 44.2 ka (cal. BP 95% probability) for Layer 3, 4 and 5 respectively. However, in the unmodeled ages there was an age inversion in the radiocarbon dates of Layers 4 and 5 with no obvious resolution.[8] While the quality indicators of the ^{14}C dates and extracted collagen do not suggest any issues with the reliability of the dates, the geological features of the sediments do not indicate a reworking of Layers 4 and 5.[8] At the same time, the ^{14}C ages for each layer are internally very consistent, indicating a lack of mixing with other deposits and making a reworking hypothesis less likely. Unfortunately, this contradiction cannot be solved at this time, meaning that the ages of Layer 4 should be treated with caution at this point. Based on the geological analyses of the layer sediments we retain the stratigraphic order of these layers when presenting stable isotope results. It should also be noted that for the purposes of the interpretations in our paper, smaller shifts of the age of Layer 5 within MIS 3 do not affect any of our main conclusions.

The Middle Paleolithic stone tool assemblages show a consistently high amount of Levallois blank production compared to other blank production technologies (Supplementary Figure S1 B), and it remains the most frequent production technology with small variations between ca. 10 and 20 % across different layers. Small amounts of Discoid and Type Five blank production are present in all MP layers to varying degrees, but are not nearly as abundant as Levallois produced pieces in any MP layer. The relative abundance of scrapers out of all stone tools (flakes with retouch) shows considerable variability across the MP sequence, ranging from relatively low abundances of 37 % and 30% of plotted tools in Layers 1 and 3 but higher proportions close to 50 % or more in Layers 2, 4 and 5 (Supplementary Figure S1 B). The characteristics of the La Ferrassie Middle Paleolithic lithic assemblages are consistent with what has been described as Levallois Mousterian. However, it should be noted that the relative abundance of both Levallois produced lithics and scrapers reported here are often lower than what Peyrony reported and used to define the La Ferrassie Mousterian facies; this discrepancy is likely due to selection bias present in these older collections.[9]

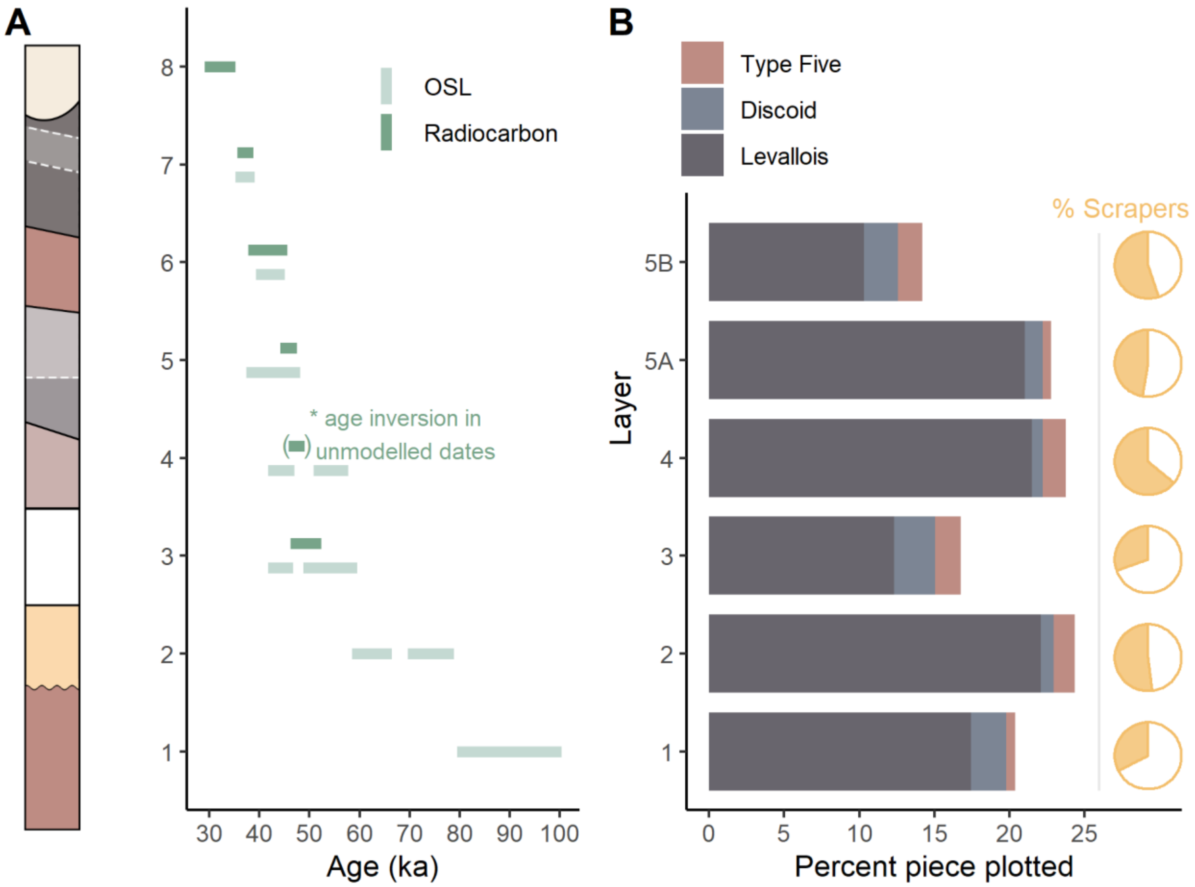

Supplementary Figure S1:Excavations of the Western Sector uncovered a stratigraphic section (A) of 9 Middle and Upper Paleolithic layers (Layer 9 not shown due to spatially restricted extent of this layer) with ages covering late MIS 5 to early MIS 2 (A). Solid black lines in the stratigraphy schematic denote layer boundaries, while dashed white lines demarcate sub-unit divisions. The Middle Paleolithic layers examined in this study are dominated by Levallois blank production with moderate to high proportions of scrapers (B; the orange portion indicates percent scrapers out of the total number of tools per layer). Radiocarbon dates are presented following Model 2 in 8. Note that there was an age inversion in the unmodelled ^{14}C dates between Layer 4 and 5 that remains unresolved. Radiocarbon ages for Layer 4 therefore should be treated with some caution.

The density of anthropogenic artifacts as exemplified by piece plotted (larger than 2.5 cm) bone fragments and stone tools shows a noticeable change across the Middle Paleolithic sequence, with a pronounced increase in artifact density from Layer 4 onward, with the highest artifact density in Layer 5B (Supplementary Figure S2). Indeed, an increase in the amount of anthropogenic inputs was used to differentiate between Layer 5A and 5B, layers that in terms of their sedimentary matrix are otherwise considered to be the same stratigraphic unit.

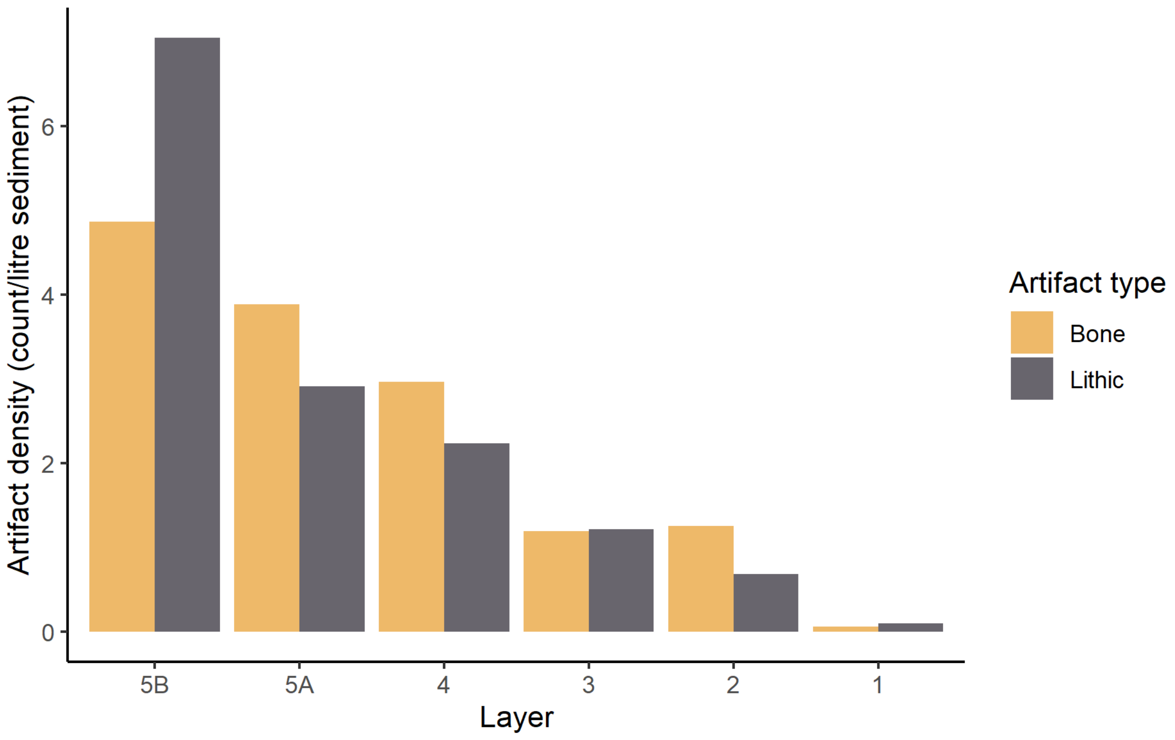

Supplementary Figure S2:The density (count per liter of sediment) of piece plotted bone fragments and lithics steadily increases throughout the Middle Paleolithic sequence of La Ferrassie, with highest densities in Layer 5B.

2. Geology

During the latest excavations, we recognized nine stratigraphic units based on standard lithological criteria, such as color, composition, texture, and the geometry of the sedimentary body. We provide in Supplementary Table S1 descriptions and working interpretations of units 1-5 (those from the Middle Paleolithic).

Supplementary Table S1: Generalized summary of La Ferrassie stratigraphy and site history.

Strat. unit	Industry	Description	Comments
5B	Ferrassie Mousterian	• Compact bedded yellowish red brown silty sand with abundant ~cm-sized fragments of lithics and bone<br>• More granular in appearance and richer in rounded limestone pebbles than 5A<br>• The finer fraction is composed of calcite and quartz sand intermixed with calcerous clay and is proportionally richer in 5B than 5A,	• Dry fall talus cone whose origin is a platform emanating from upper cave but whose apex was near the present day road

		which has a higher proportion of coarse anthropogenic elements (bone, lithics)	
5A	Upper Mousterian	• Reddish yellow pebbly silty sand composed of calcite and quartz sand intermixed with calcerous silty clay with generally platy limestone clasts and abundant bone fragments<br>• Richer in anthropogenic inputs (bone, lithics) than 5B	• *Idem*
4	Ferrassie Mousterian	• Massive, compact silty medium sand with relatively abundant cm-sized pieces of bone and flint, which are larger than those in Layer 5<br>• Clearly truncates Layer 3 in Sq. I4<br>• Upper half of Layer 4 is banked up against the roof fall and is generally horizontal	• Large collapse of roof after initial accumulation of Unit 4<br>• Common inputs from human occupation
3	Bifaces and large flakes	• Clear contact with Layer 2 below<br>• Poorly sorted light brown silty sand with pebbles and cm-sized fragments of Mn-stained, angular burned bone fragments.<br>• Inclined to SSE<br>• Increasingly stony and flint- and bone-rich to north	• Mudflow derived from NW<br>• Source area is no longer visible<br>• Roof continues to collapse
2	Bifaces and large flakes	• Cemented stony sand with lenses of coarser limestone, which climb upward in the profile; some well-rounded cm-sized gravel<br>• Increasingly limestone rich toward the cave wall<br>• Unconformity between Layers 1 and 2<br>• The base of this unit is inclined from NW to SE; bones also show orientation in this direction<br>• Deposits are subhorizontal but contact dips to the S	• They are inclined from NW to SE, thus pointing to a source of this sediment from the NW, in the direction of the present day road<br>• The original source material was removed during construction of the present day road<br>• Solifluction deposit pene-contemporaneously affected by cryoturbation and ice-lensing that also affected the top of Unit 1 (cold climate)<br>• Roof starts to collapse
1B	Bifaces and large flakes	• Upper part truncated by the contact with Layer 2<br>• Locally red sand (with limestone *éboulis*) locally laminated and with pockets and concentrations of iron grains/pisolites	• Fluvial with contributions of roof fall and slabs derived from the roof.
1A	Sterile?	• Dark red sand (with limestone *éboulis*) locally laminated and with pockets and concentrations of iron grains/pisolites	• Fluvial with contributions of roof fall and slabs derived from the roof.

Layer 2 represents a distinct cold episode in a number of ways. It is composed essentially of finely comminuted limestone grains derived from disintegration of limestone at its source to the NW. Here, bedrock or blocks of massive limestone roof collapse must have existed in the area of the current departmental road, but their remains were removed during road construction during the end of the 19th c. Owing to the fresh, unweathered nature of the limestone grains, this fragmentation was mechanical in nature, likely produced by freeze-thaw processes. Similar microscopic indicators of distinctly cold climate with alternating freeze-thaw include banded fabrics and calcareous silt cappings on the upper surfaces of coarse components (Supplementary Figure S3 and Supplementary Figure S4). Stratigraphic contact and orientations analyses, alongside the microscopic evidence of silt cappings and banded fabric and field observations, clearly associates the deposition of Layer 2 with pronounced cold conditions by solifluction – a downslope slow creep process typical of periglacial landscapes. It can be excluded that cryoturbation features in this Layer represent a reworking by later freeze-thaw processes. The last 'stratigraphic phase' is the formation of ice lenses and silt cappings, which show no signs of disturbance since their formation.

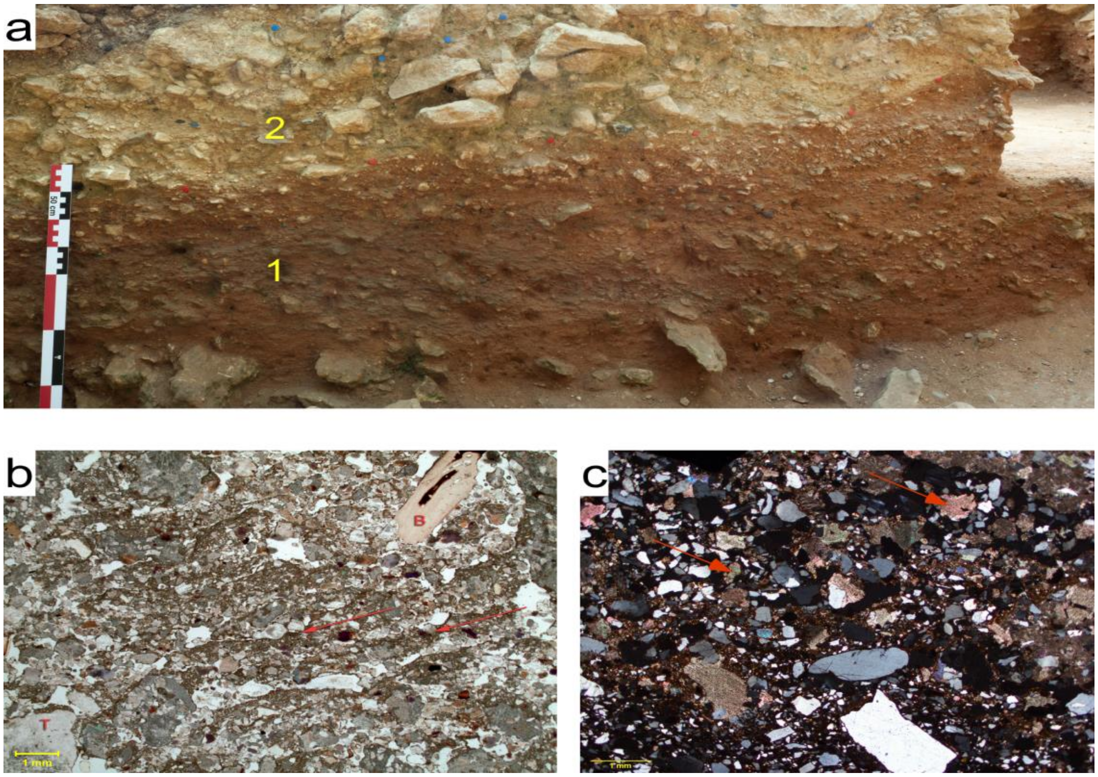

Supplementary Figure S3:a) Field view of Layers 1 (reddish brown) and 2 (yellowish white) in Squares H5-G5 showing cryoturbated aspect of Layer 2 and its sharp, undulating contact with underlying Layer 1 indicative of solifluction. Note the finely comminuted nature of the limestone particles in Layer 2. Scale has 10 cm major increments, 1 cm minor increments; b) Photomicrograph of Layer 2 deposits showing ice lensing (red arrows) that produces banded fabric (lenticular aggregates separated by segregation voids) in silty granular deposits rich in limestone sand; a bone (B) is at upper right. Plane polarized light (PPL), scale is 1 mm; c) Photomicrograph of Layer 2 deposits in cross-polarized light (XPL). Note the abundance of angular calcareous sand (red arrows) indicative of mechanical disintegration of limestone produced by freeze-thaw. Scale is 1 mm.

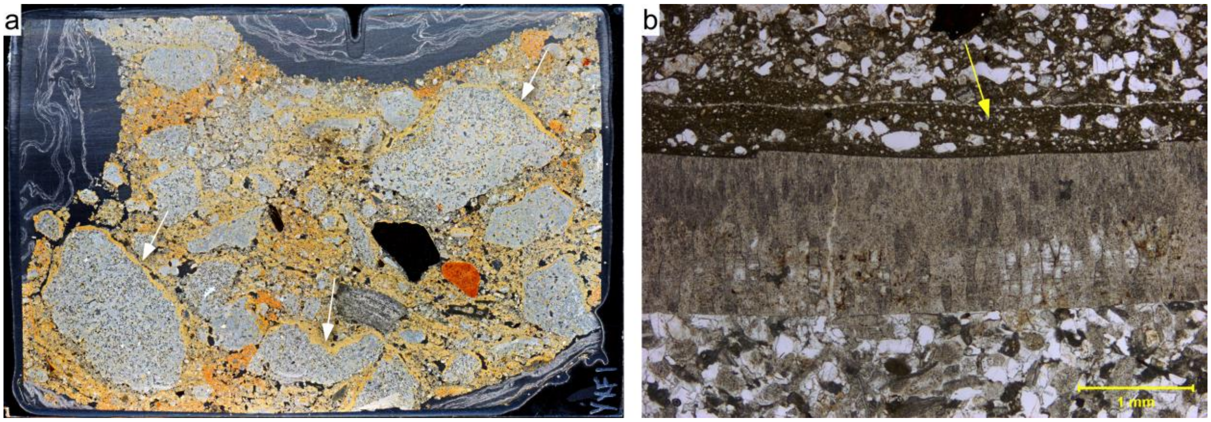

Supplementary Figure S4:a) Thin section scan (dark field illumination) of sample LF10-1 from Layer 2 illustrating silt cappings on fresh limestone clasts (white arrows). Size of thin section is 75 x 50 mm. b) Photomicrograph of Layer 2 deposits showing thick calcareous silt capping, indicatirng cold, freeze-thaw conditions, on an oyster fragment derived from the local limestone (yellow arrow). Plane-polarized light (PPL); scale is 1 mm.

Layer 5 is subdivided into two subunits, 5A and 5B, that are similar in their sedimentary matrix but differ in terms of fine fraction and density of anthropogenic inputs, the latter are more abundant in 5A. The deposits vary in thickness from 50 cm to ~30 cm near the wall and are comprised of silty sands with dm-sized roof fall. Deposition relates to slope dry-fall processes with a depositional cone emanating from the platform existing somewhere in front of the Upper Cave. Both field and micromorphological observations do not reveal any indications of cold-associated features.

3. Faunal spectrum

The relative proportion of the main prey taxa in the faunal assemblage (taxon identifiable ungulates with >1% abundance) remains very consistent across the Middle Paleolithic layers of La Ferrassie and is dominated by temperate adapted fauna such as aurochs/bison (*Bos sp./Bison sp.*) and red deer (*Cervus elaphus*)(Supplementary Figure S5). Roe deer (*Capreolus capreolus*) occur in smaller numbers, with their highest proportion in Layer 2. Reindeer

(*Rangifer tarandus*) are found in all Middle Paleolithic layers, but never in large numbers even in the MIS 4 attributed Layer 2. This is in contrast to other southern French sites such as Pech de L'Azé IV or Roc de Marsal, where MIS 4 attributed layers are often strongly dominated by reindeer remains.[10,11] The absence of reindeer in favor of more temperate adapted species indicates that the faunal assemblage accumulated during rather mild environmental conditions with at least some forest cover that enabled forest adapted species such as roe deer to be present in the region. While expected for the upper part of the Middle Paleolithic sequence, which falls into MIS 3, this is a rather unexpected result for the MIS 4 associated Layer 2.

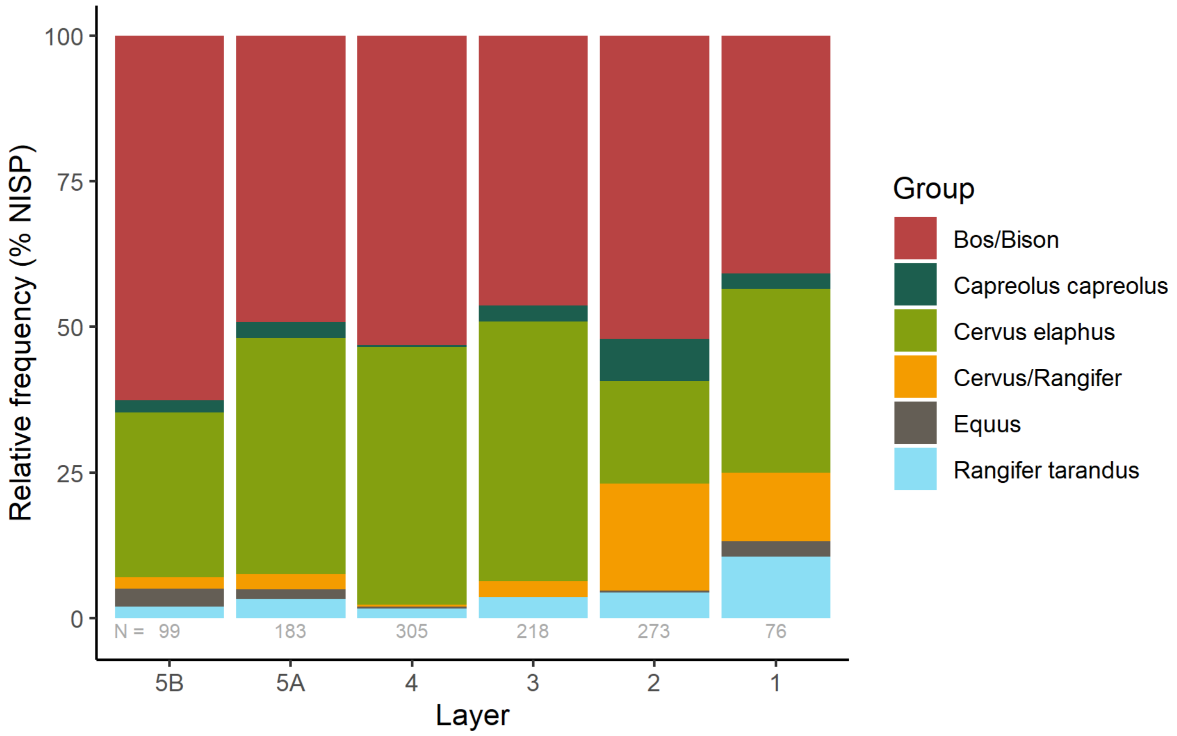

Supplementary Figure S5:Relative frequency of major (on average >1 % abundance) ungulate taxon groups across the Middle Paleolithic layers show a faunal spectrum dominated by temperate species with little diachronic variation.

4. Bone surface modifications

Surface modifications on bone fragments (> 2.5 cm size cut-off) are dominated by human modifications (cut-marks, scraping, percussion notches, anvil-marks, use as a retoucher)

rather than carnivore modifications (chewing, punctures, digestive etching) in all Middle Paleolithic layers and across body size classes (Supplementary Figure S6, top). This demonstrates that the faunal assemblage is predominantly of anthropogenic origin rather than from carnivore accumulation, and is therefore tied directly to human occupation and activity at the rock shelter. It should be noted that sample sizes of small ungulates are often small and differences in relative frequency of surface modifications from other body size classes may not represent a meaningful pattern. Impact notches on long bones can serve as an indicator of marrow extraction from animal bones. At La Ferrassie bones of large ungulates show substantially less percussion marks than medium or small ungulates (but note small sample sizes of small ungulates). Like other features of the faunal assemblages, this feature is relatively consistent throughout the Middle Paleolithic sequence with only minor changes between different stratigraphic layers (Supplementary Figure S6, bottom). This suggests a relative stability in the intensity of marrow extraction and carcass processing intensity to extract nutrients. This may also indicate that nutritional stress of Neandertals at the site varied little over time.

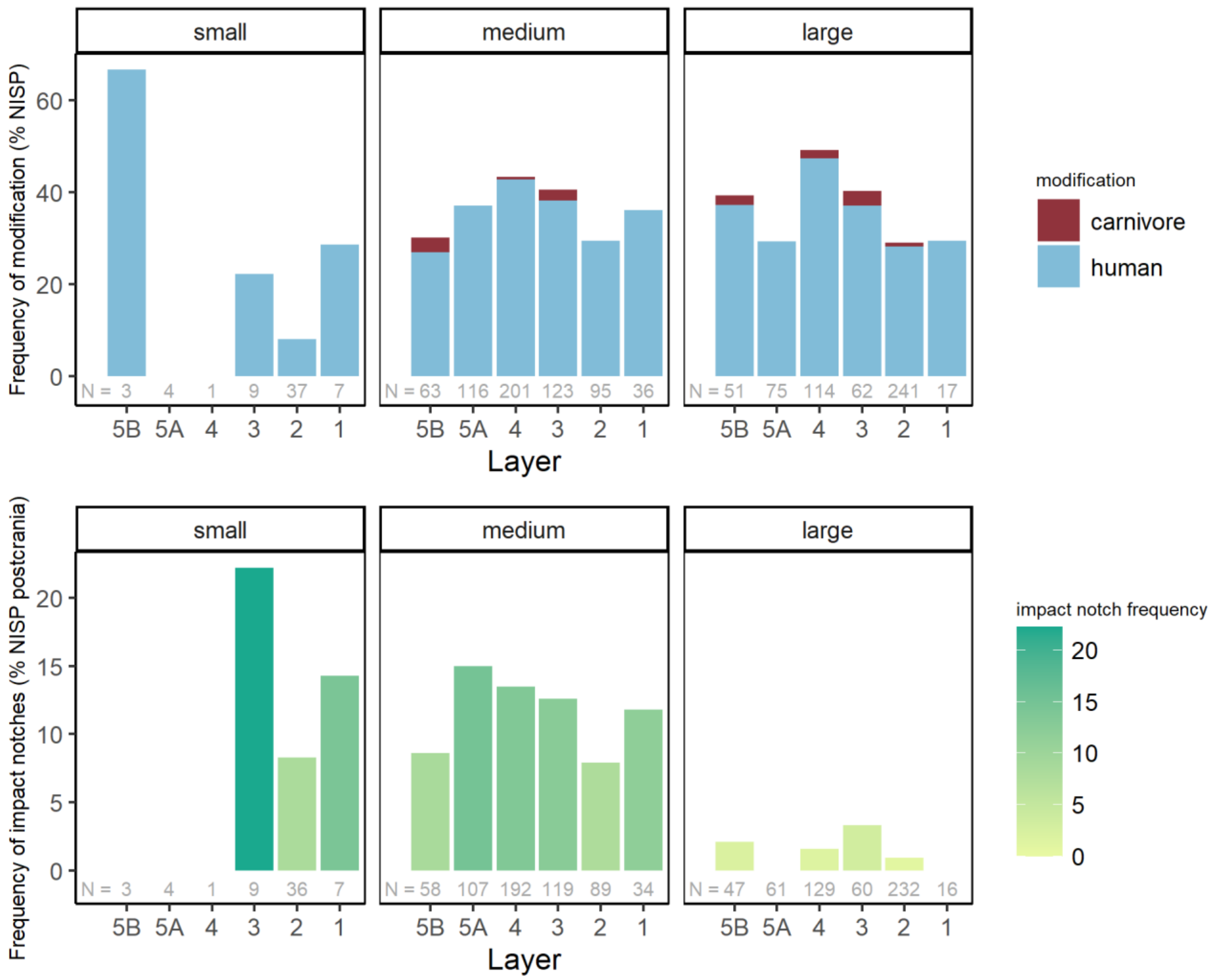

Supplementary Figure S6:Relative frequencies of human (blue) and carnivore (red) surface modifications on ungulate bones relative to total NISP (printed below bars) grouped by layer (panels) underline the anthropogenic nature of the La Ferrassie faunal assemblage. Bar heights indicate the proportion of fragments with a particular modification and total NISP counts exclude isolated teeth and antler fragments. Small ungulates include roe deer, medium ungulates include red deer and reindeer, large ungulates include Bos/Bison and horses.

5. Oxygen stable isotope methodology

Tooth enamel of *Bos/Bison* teeth from three archaeological layers (Layer 2, 5A and 5B; see Supplementary Table S2) was sequentially sampled ($n_{samples}$ = 178, n_{teeth} = 13) to generate paleotemperature estimates from $\delta^{18}O$ of bioapatite phosphate. A mixture of *Bos/Bison* third molars, second molars and 4th premolars were chosen from layers where several suitable teeth were available. Due to the low number of suitable teeth available from the site, our sampling strategy was focused on sampling a larger number of teeth even of different tooth

positions that could potentially belong to the same individual. To reduce as much as possible the potential for repeated sampling of the same animal, we used tooth wear and similarity of stable isotope results in addition to tooth position to determine the minimum number of sampled individuals and number of distinct years of isotopic input represented in our study (see Supplementary Table S7, Supplementary Table S8 and Supplementary Table S9). In almost all cases with only one exception in Layer 2, it could be shown that attribution of several teeth to the same individual is very unlikely. Thus, based on the listed criteria, we determine that the oxygen stable isotope data in this study stem from a minimum of 12 distinct individuals.

Prior to sampling, teeth dental calculus was removed in the sampling area using gentle abrasion with a diamond tipped drill bit. Teeth were then repeatedly sonicated in Milli-Q ultrapure water to remove sediment residue. To obtain tooth enamel samples for oxygen and strontium isotope analysis sequential samples were drilled in small strips (ca. 8 x 1.5 x 0.7 mm) perpendicular to the tooth growth axis using a diamond tipped drill bit. Series of sequential samples were drilled covering the complete crown length of one tooth loph. An example of a tooth before and after sampling can be seen in Supplementary Figure S7.

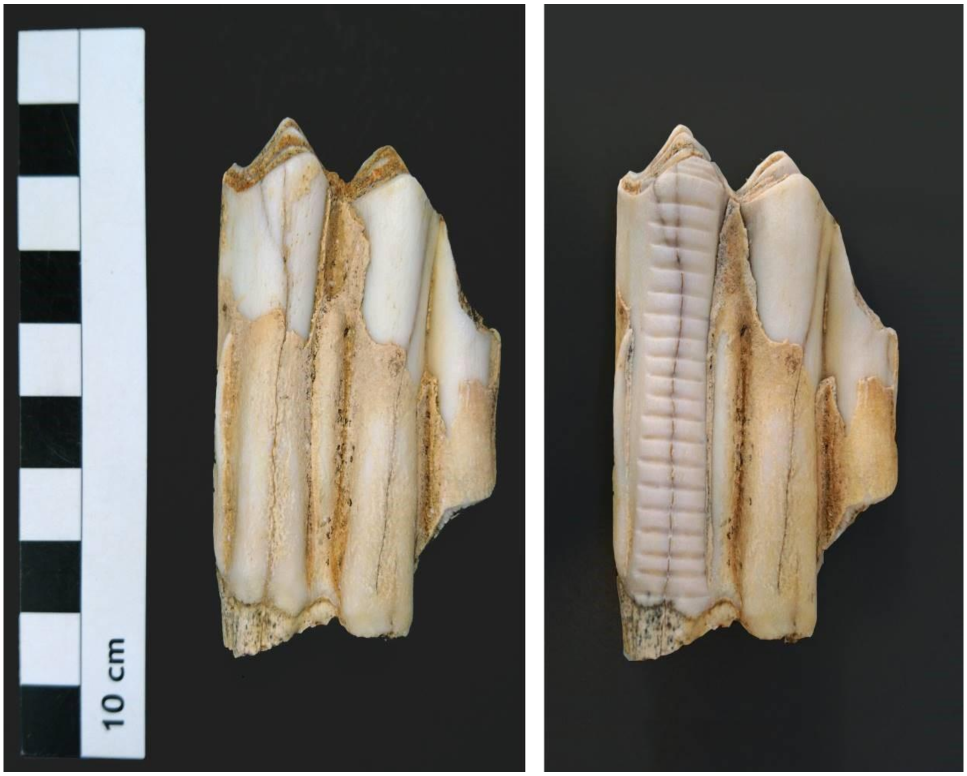

Supplementary Figure S7:Example photographs of a *Bos/Bison* molar (F7-294) before (left) and after (right) serial sampling for oxygen and strontium isotope analysis.

Tooth enamel powders were converted to silver phosphate (Ag_3PO_4) for oxygen isotope measurements of bioapatite phosphate following an adapted version of the rapid precipitation protocol developed by 12 and modified by 13. Approximately 5 mg of tooth enamel powder of each sample was weighed into 2 mL Eppendorf microcentrifuge tubes. To obtain a phosphate solution and remove calcium as the samples were digested in 0.4 mL of 2 M hydrofluoric acid (HF) on an agitator for 24 hours. The phosphate containing solution was then removed from the resulting calcium fluoride (CaF_2) residue and transferred into a new microcentrifuge tube. To maximize phosphate recovery the CaF_2 residue was washed once with 0.1 mL MilliQ ultrapure water and the wash added to the phosphate containing solution. The sample solution was then neutralized as indicated by the color change point of Bromthymolblue indicator using 25% ammonia solution (NH_4OH). 60 µL of NH_4OH were first added to each sample using an automatic pipette after which the solutions was slowly titrated to the color change point using individual drops of NH_4OH added with a 100 µL Hamilton Microliter fixed needle syringe (Hamilton Bonaduz AG, Switzerland). After

neutralization, Ag_3PO_4 was crash precipitated by addition of 0.4 mL 2 M silver nitrate ($AgNO_3$) solution. The resulting precipitate was pelleted by centrifugation (12000 rpm for 5 Min) and the silver nitrate supernatant removed. The silver phosphate was then washed three times with MilliQ ultrapure water using centrifugation and vortex mixing steps between rinses to eliminate any silver nitrate from the sample. Silver phosphate samples were then dried over night at 50 °C and stored over desiccant until further analysis.

Oxygen isotope ratios of Ag_3PO_4 were analyzed using a high temperature elemental analyzer (TC/EA) coupled to a Delta V isotope ratio mass spectrometer via a Conflo IV interface (Thermo Fisher Scientific, Bremen, Germany) at the Max-Planck-Institute for Evolutionary Anthropology (MPI-EVA). Approximately 0.5 mg of each silver phosphate sample was weighed into cleaned silver capsules (3x4 mm, IVA Analysentechnik, Meerbusch, Germany) and introduced to the TC/EA using a Costech Zero Blank Autosampler (Costech International, Cernusco sul Naviglio, Italy). Conversion to CO was achieved using a reactor temperature of 1400 °C and gases separated using an Agilent Technologies 0.6 m x 1.4" x 4 mm stainless steel GC column with 80/100 mesh 5 Å molecular sieve packing (IVA Analysentechnik, Meerbusch, Germany) maintained at 80 °C with a column carrier gas flow of 100 mL/min. Samples were usually measured in triplicate except in cases where additional measurements were conducted to improve the measurement precision or if individuals measurements failed to conform to quality control criteria such as acceptable sample amount to peak area relationship. Oxygen isotope delta values were two-point scale normalized to the VSMOW scale using a commercially available Ag_3PO_4 standard (B2207, $\delta^{18}O = 21.7 \pm 0.3$ ‰, 1 s.d.; Elemental Microanalysis, Okehampton, UK) and an in-house Ag3PO4 standard (KDHP, $\delta^{18}O = 4.2 \pm 0.3$ ‰) whose accepted value was previously calibrated to B2207 and IAEA-SO-6 (barium sulfate, $\delta^{18}O = -11.35 \pm 0.3$ ‰, 1 s.d. as reported in 14). A commercial silver phosphate was used as a quality control standard (Sigma Aldrich silver phosphate AS337382, $\delta^{18}O = 14.0 \pm 0.3$ ‰, Sigma Aldrich, Steinheim, Germany) in addition to aliquots of NIST SRM 120c (previously NBS 120c, $\delta^{18}O$ reported between 19.9 and 22.6 ‰ with a consensus value of 21.7 ‰[15]) and an in-house cow enamel standard which were precipitated with equal

treatment alongside each sample batch. Measurements of the quality control standards gave values of 14.0 ± 0.2 ‰ for AS337382 (n = 156), 15.3 ± 0.2 ‰ for BRWE (n = 44) and 21.8 (n = 43) ± 0.5 ‰. Reproducibility of replicate measurements of each sample was 0.2 ‰ on average.

6. Oxygen isotope intratooth profiles

Using a serial sampling approach, series of time-dependent $\delta^{18}O$ measurements were generated for each sampled *Bos/Bison* tooth to extract seasonal temperature information (Supplementary Figure S8). Due a pronounced temperature effect $\delta^{18}O$ values of precipitation vary seasonally with high values indicating the summer season and low values indicating the winter season.[16–18] This is in turn recorded in tooth enamel $\delta^{18}O$ values of obligate drinking animals such as *Bos/Bison*, which are strongly and linearly linked to the oxygen isotopic composition of drinking water, as this forms the largest contributor to body water.[19,20] The incremental growth and lack of remodeling in tooth enamel additionally enable a sequential sampling approach in which a series of small samples is taken along the tooth growth axis to obtain a time-dependent series of $\delta^{18}O$ measurements.[21,22] In *Bos/Bison* such $\delta^{18}O$ time series can cover a time frame of a few months to over one year, depending on the formation time of the teeth used.[23] Time-dependent $\delta^{18}O$ measurement series obtained in this way consequently have sub-annual time-resolution and enable the extraction of seasonal (summer and winter) temperature estimates. All individuals studied here show clear full or partial sinusoidal ^{18}O curves, with the exception of one individual (F7-14). The corresponding data is additionally displayed in Supplementary Table S4. Seasonal temperature information can therefore be extracted from minima and maxima of each sinusoidal $\delta^{18}O$ curve. Summer peaks and winter trough $\delta^{18}O$ values were obtained by visual inspection of sinusoidal curves to determine the maximum and minimum values where the $\delta^{18}O$ curve undergoes a clearly visible turn (the exact location for each extracted point is marked in Supplementary Figure S8). As not all teeth preserve full annual cycles, not every tooth could yield both summer and winter seasonal $\delta^{18}O$ information, which leads to

differences in the number of total data points extracted for summer and winter $\delta^{18}O$ values in each layer. Mean annual $\delta^{18}O$ values were obtained by averaging summer and winter points, analogous to standard procedures to calculate mean annual temperature.

Climatic information from seasonal $\delta^{18}O$ curves can either be interpreted in a relative manner, by comparing raw $\delta^{18}O$ values of individuals from different archaeological layers or different sites to explore diachronic temperature change or differences between sites. This allows for nuanced comparisons and distinction of even relatively small climatic differences, but can only be applied within the same or very closely related species, due to the species-specific isotopic fractionation between drinking water and body water.[20,24–26] This limits the quantitative comparisons that can be made with other sites, especially considering the small number of $\delta^{18}O$ data sets from animal bioapatite phosphate that are available for the Middle Paleolithic. To facility broader comparisons within the context of Late Pleistocene climate records generated from other proxies, raw $\delta^{18}O$ values need to be converted to absolute temperatures (see below), which introduces a larger amount of uncertainty.

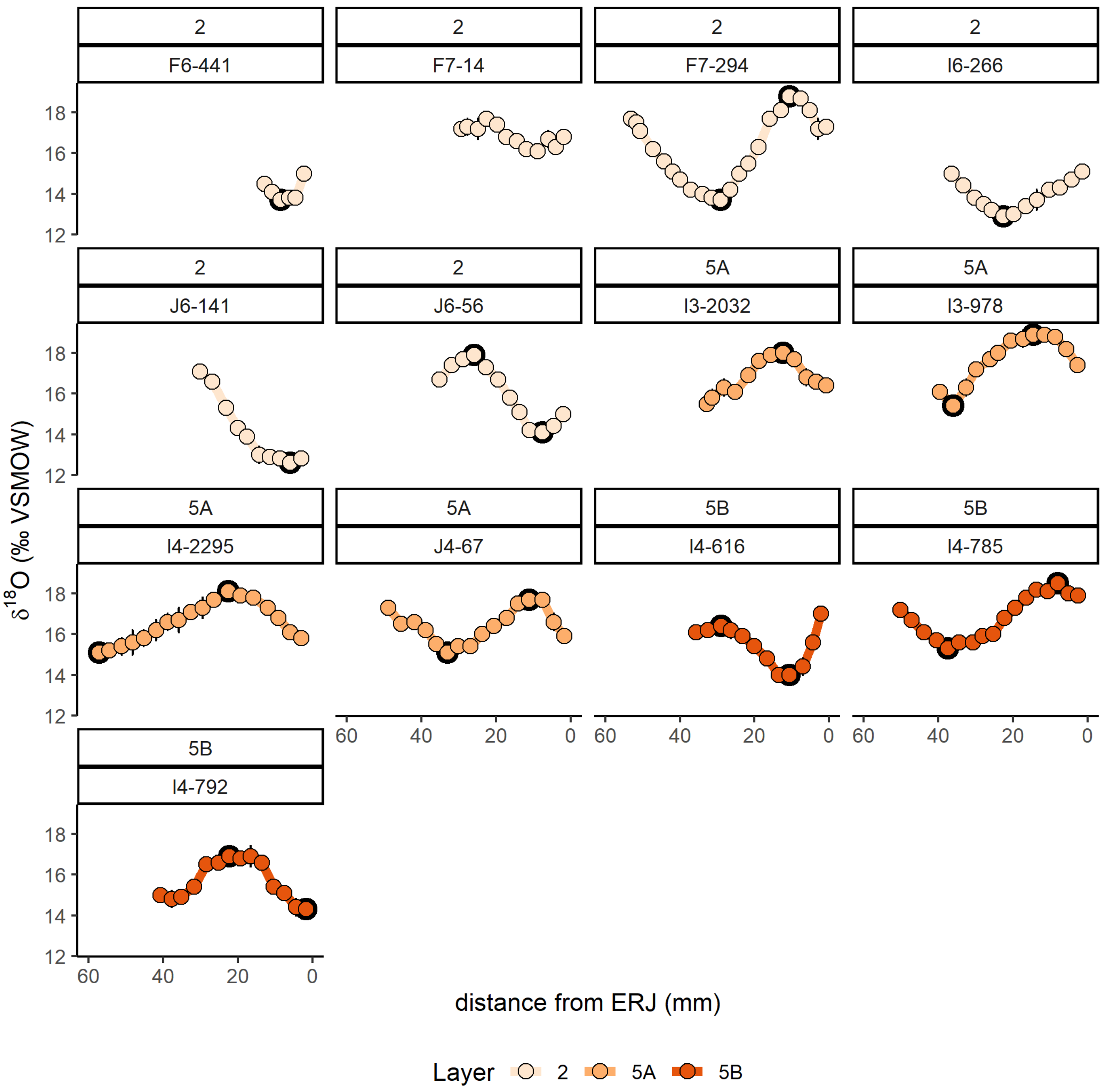

Supplementary Figure S8:Sequentially sampled tooth enamel of *Bos/Bison* individuals shows sinusoidal curves of seasonal change in δ^{18}O. δ^{18}O values are plotted relative to distance from the enamel-root-junction (ERJ). Individuals are ordered and colored by layer (beige: Layer 2, orange: Layer 5A, red: Layer 5B). Error bars (often smaller than point symbols) represent one standard deviation around the mean of replicate measurements. Summer peaks and winter troughs that were extracted for seasonal δ^{18}O analysis and temperature reconstruction are marked with bold symbols.

7. Inverse modeling of intratooth oxygen stable isotope profiles

Sequential sampling of herbivore tooth enamel necessarily introduces a certain amount of time averaging of the $\delta^{18}O$ signal. This is due to the nature of enamel formation (amelogenesis) and the sampling strategy. Due to the complex and extended multi-phase process of enamel formation and maturation, where most of the mineral mass - and therefore the dominant isotopic inputs into tooth enamel - is added in a diffuse process that does not follow the visible incremental geometry of enamel apposition, but is continuously averaged to a varying extent over the enamel maturation process.[27–35] It is therefore not possible for samples to capture discrete time intervals or biological increments. Rather, each sample necessarily represents a homogenized average of isotopic input over the complete duration of enamel maturation at the sampling point, largely independent of sample volume[28,34–36] This means that a sample series is a significantly time-averaged representation of the original isotopic input into animal tooth enamel.[20,31,33,34,36–38] This results in a predictable decrease of the amplitude (dampening) and an offset in the position of peaks and troughs of the sinusoidal $\delta^{18}O$ curve recorded in each tooth, which needs to be corrected before faithful absolute seasonal temperatures can be reconstructed.[33,34,38–42] If a consistent sampling strategy is used, a correction is not necessary for a relative comparison within the same species (and ideally the same tooth type), as the amount of time averaging is approximately consistent between all studied teeth.

To correct for this dampening effects on the amplitude of intratooth $\delta^{18}O$ profiles, an inverse modeling procedure using adapted version of code published in 39 was employed to reconstruct the original $\delta^{18}O$ input time series. This approach reconstructs the original $\delta^{18}O$ input into tooth enamel by reverse modeling the time averaging introduced through the sampling procedure by taking into account the sampling geometry and species-specific parameters of enamel formation. A detailed description of the modeling procedure with associated code can be found in 39. It should be noted that this inverse model was originally developed for ever-growing teeth and does not take into account the slowing of enamel growth towards the enamel-root-junction (ERJ) seen in some molars of large ungulates,

particularly horses.[40,43] A more recent alternative model developed for sheep molars improves on these aspects,[41] however the parameters necessary to growth rate in this model are currently unknown for large Bovidae such as cattle or bison. At the same time, studies of sheep teeth have shown that divergence between expected results and the outputs of the 39 model is mostly relevant for rapid short terms shifts in the isotopic input, but relatively limited for slow large scale shifts in isotopic input.[44] Application to reconstruct exclusively amplitudes of $\delta^{18}O$ seasonal curves should therefore yield sufficiently close approximations of input $\delta^{18}O$ seasonal change.

Enamel formation input parameters were chosen to reflect *Bos/Bison* amelogenesis following values given in 34 and 38. Initial enamel density was set at 24.6 %, enamel appositional length as 1.5 mm, and maturation length as 25 mm. Additionally, sample input variables were given for distance between samples and sample depth. During the modeling procedure a damping factor describing the damping of the isotopic profile amplitude needs to be chosen using and adjustment of a measured error term (E_{meas}) to the prediction error (E_{pred}). The adjusted damping factors determined for the specimens analyzed here fell between 0.001 and 0.015. Graphical representations of the modeled ^{18}O input time series can be found in Supplementary Figure S9.

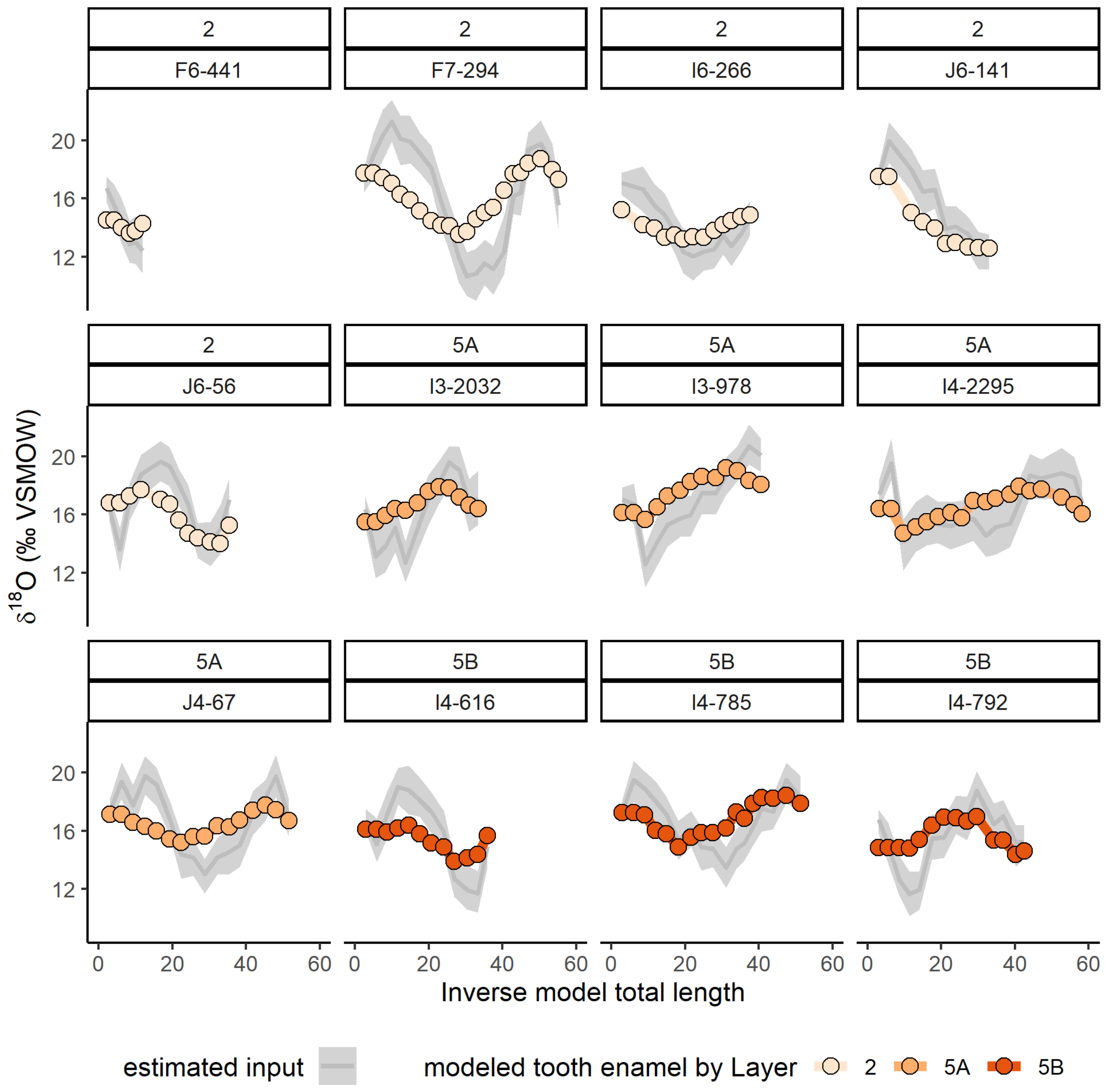

Supplementary Figure S9:Mean estimated δ^{18}O input (grey line) with 95% confidence interval (grey shaded area) and dMeasd variable model enamel δ^{18}O values (circles) from Passey et al. [39] inverse model.

8. Oxygen isotope analyses as a temperature proxy

While raw δ^{18}O values of individuals from different layers can be used to examine temperature change through time in a relative manner, a conversion to absolute temperatures is necessary to compare results with other paleotemperature records and modern day climates. This conversion relies on the empirically defined linear relationships

between temperature and precipitation $\delta^{18}O$ as well as between drinking water $\delta^{18}O$ and animal tooth enamel $\delta^{18}O$.[19,24,25,45–49] These relationships can be formalized in regression equations and inverted to yield temperature from tooth enamel $\delta^{18}O$. Both the regression procedure and inversion can be accomplished using different statistical approaches (see 46 and 47). In this study we employ an inverted forward regression approach, where each regression line is established in the original causal direction and the resulting equation is then inverted to yield drinking water $\delta^{18}O$ from tooth enamel $\delta^{18}O$ and temperature from drinking water/precipitation $\delta^{18}O$, following methods in 46. Individual estimates for $\delta^{18}O_{dw}$ are pooled for each archaeological layer to yield an average temperature estimate for each layer with compound uncertainty. Compound uncertainties for layer temperature estimates are computed also following methods in 46 and are used in order to quantify the propagated uncertainties incurred through both regression steps. For each regression step, appropriate species-specific and regional modern calibration data sets have to be chosen. Regression lines used in this study were established using a tooth enamel $\delta^{18}O$ to $\delta^{18}O_{dw}$ calibration data set that combines published data for Bison[19] and Cattle[50] as well as a the European air temperature to $\delta^{18}O_{precip}$ calibration data set published in 46. To obtain summer, winter and mean annual temperature estimates, summer peaks, winter troughs and mean annual values from the inverse model described in Supplementary Text 7 were used as input values for the temperature conversion. Summer peaks and winter troughs were isolated as maximum and minimum values in the locations corresponding to the peak and trough that were used in raw $\delta^{18}O$ curves (see Supplementary Text 6). Mean annual values were obtained by averaging these minimum and maximum model values, analogous to standard procedures of calculating mean annual temperature.

The use of $\delta^{18}O_{enamel}$ as a paleotemperature proxy relies on a number of specific assumptions related to animal physiology and drinking behavior, local hydrology, and atmospheric circulation. Firstly, the use a temperature proxy relies on the fact that studied animals drink relatively large amounts of water and that this water generally reflects $\delta^{18}O$ of precipitation. This is a necessary model assumption, as the effect of temperature on $\delta^{18}O$ is established

using $\delta^{18}O$ of precipitation, but this effect can vary between different types of water sources. Large Bovidae such as cattle or Bison are obligate drinkers and have large minimum daily water requirements and a very limited ability to meet substantial portions of their water requirements from other sources.[51–54] As such they have been reported to regularly consume large quantities of water by drinking across a variety of ecological and environmental settings[51–54] and their enamel $\delta^{18}O$ is strongly tied to $\delta^{18}O$ of local precipitation.[19,50] This demonstrates that cattle and bison generally consume large enough quantities of open surface waters, that their $\delta^{18}O_{enamel}$ values can reasonably be assumed to reflect $\delta^{18}O$ of liquid drinking water, with a negligible contribution from water contained in food or from structurally bound oxygen in food. Given the metabolic constraints on this characteristic and its apparent lack of variability across modern ecosystems, Bos/Bison drinking behavior is likely relatively conservative and can be assumed to have been similar in the Late Pleistocene. Similarly, the contribution of leaf water to the total water intake is directly related to daily food intake, driven by energy requirements and leaf water content – both factors that given are unlikely to be more different between the Late Pleistocene in Europe and today than across different modern day ecosystems. This supports that it is reasonable to assume that $\delta^{18}O$ of *Bos/Bison* tooth enamel is strongly tied to $\delta^{18}O$ of drinking water and that this relationship is applicable to a reasonably large variety of environments and time scales, including those found in the European Late Pleistocene.

The secondary assumption is then, that surface waters consumed by *Bos/Bison* are broadly reflective of $\delta^{18}O_{precip}$, which is the case for the majority but not all surface waters.[55] Notable exceptions are deep groundwaters, large rivers and large lakes, which can be isotopically decoupled from local precipitation due to effects of water transport, water residence time and evaporation.[56–58] As there are no large lakes present in the area of the site this leaves three potential scenarios of water source choice could lead to divergence of $\delta^{18}O_{dw}$ from $\delta^{18}O_{precip}$: 1) obtaining drinking water from deep groundwater fed springs, 2) occupation of areas with different $\delta^{18}O_{precip}$, such as areas on plateaus with higher elevation or 3) drinking water from rivers, in this case the nearby Vézère River. Pronounced seasonal signals seen in

the $\delta^{18}O_{enamel}$ time series (Supplementary Figure S8) demonstrate that *Bos/Bison* in this study did not regularly consume drinking water from seasonally buffered water sources such as deep groundwater. Using strontium isotope analysis we can exclude that *Bos/Bison* were ranging in higher elevation areas to the northeast of the site (see Supplementary Text 10). This leaves the possibility of consumption of water from the Vézère River or possibly the Dordogne river. The Vézère River flows within ca. 5 km distance of the La Ferrassie, while the Dordogne river is approximately 15 km distant from the site. Consumption of water from the Dordogne river would have a stronger impact on $\delta^{18}O$ patterns, as this river has its source area at higher elevation and greater distance from the site of La Ferrassie and would likely be isotopically more different from local $\delta^{18}O_{precip}$ than water from the Vézère River. However, strontium isotope ratios in *Bos/Bison* tooth enamel suggest that substantial consumption of Dordogne river water or of plants growing in the Dordogne valley are unlikely. A $^{87}Sr/^{86}Sr$ isoscape of bioavailable strontium (Supplementary Figure S12) as well as $^{87}Sr/^{86}Sr$ measurements directly from the Dordogne river[59] show that the river as well as sediments deposited in the valley exhibit $^{87}Sr/^{86}Sr$ values that are higher (ranging from 0.715 to 0.720) than $^{87}Sr/^{86}Sr$ in La Ferrassie *Bos/Bison* tooth enamel (ranging from 0.7100 and 0.7109). As *Bos/Bison* spending substantial amounts of time in the river valley would ingest strontium from both river water, suspended sediment and from plants that also source strontium from river sediments and river water[60] their $^{87}Sr/^{86}Sr$ should be substantially higher. We therefore conclude that consumption of large amounts of Dordogne river water are unlikely for the analyzed *Bos/Bison*. The same is true to an extent for the Vézère River, where strontium isotope analysis of river samples and the regional isoscape also suggest slightly higher $^{87}Sr/^{86}Sr$ values (0.713 for river samples and ca 0.712 - 0.714 in the isoscape; see 59 and Supplementary Figure S12). However, the isotopic difference from the enamel samples is not as large and the Vézère River valley is relatively small, giving more opportunity for *Bos/Bison* to range across valley adjacent areas with different baseline $^{87}Sr/^{86}Sr$ values. We can therefore not confidently exclude that *Bos/Bison* in this study were consuming water from the Vézère River. However, the impacts of river water consumption on oxygen isotope patterns would be quite limited in this case. As no direct oxygen isotope measurements are

available for the Vézére river, we make an estimation of the maximum isotopic deviation from precipitation in the low elevation plateaus around the La Ferrassie locality. The waters with lowest δ^{18}O that contribute to the Vézère River, will originate from the areas with the highest elevation in the source area of the river. Water with higher δ^{18}O will flow into the river at later points, making it progressively higher in δ^{18}O along its flow path. Therefore, while we do not know the δ^{18}O value of the river near the site of La Ferrassie, this δ^{18}O value will most likely be no lower than the δ^{18}O value of precipitation in source area of the Vézère River. The source area of the Vézère River lies at ca. 970 m a.s.l. and experiences precipitation with a δ^{18}O values of approximately – 8.5 ‰.[61] This is only approximately 1 ‰ lower than δ^{18}O of precipitation in the plateau regions around the site of La Ferrassie.[61] Considering that this isotopic difference is of comparable magnitude only as large as typical intra-individual variability within one archaeological layer and is at least 3 times smaller than the temperature induced isotopic difference expected between a glacial phase and an interglacial phase in the Late Pleistocene, we regard this effect to be negligible.

The reconstruction of air temperature from precipitation δ^{18}O in turn relies on a strong linear relationship between air temperature and precipitation δ^{18}O that occurs mid to high latitude environments.[16–18,62–64] The exact relationship between $\delta^{18}O_{precip}$ and temperature can vary slightly depending on geographic location due to secondary influences of atmospheric circulation on $\delta^{18}O_{precip}$, but the slopes of temperature to $\delta^{18}O_{precip}$ regressions are spatially remarkably stable with only small differences in slope between different mid- to high latitude regions.[55,64–68] At the same time, due to the influence of atmospheric circulation on the $\delta^{18}O_{precip}$-temperature relationship, air temperature reconstruction based on modern calibration data sets therefore also assumes a certain degree of equivalency in atmospheric circulation between modern day calibration data sets and past environments. The robustness of the $\delta^{18}O_{precip}$-temperature relationship between different regions with different atmospheric circulation regimes in modern day data sets suggests however, that the $\delta^{18}O_{precip}$-temperature relationship is stable to moderate circulation changes. In this study we employ a modern calibration data set that encompasses data from a range of

locations across Europe, and the variability in the $\delta^{18}O_{precip}$-temperature relationship contributes to the uncertainty estimates given for the final paleotemperature reconstructions.[46] Our model therefore already incorporates a numerical representation of a certain degree of variability related to impacts of atmospheric circulation on the $\delta^{18}O_{precip}$-temperature relationship to the extent that it is present across modern day localities. Although Pleistocene data on the $\delta^{18}O_{precip}$-temperature relationship is limited, simulations suggest relatively limited changes in circulation and oxygen isotope data for Pleistocene precipitation compared with temperature estimates from a variety of proxies shows little difference in the isotope-temperature relationship in the Late Pleistocene compared to modern slope estimates.[45,48,69–71] For example, Tütken and colleagues, have found a a modern day $\delta^{18}O_{precip}$-temperature slope of 0.44 compared to a slope of 0.497 estimated from groundwater $\delta^{18}O$ for the Late Pleistocene.[45] Given this data we conclude that the assumption that modern day $\delta^{18}O_{precip}$-temperature relationships can be applied to the Late Pleistocene is sufficiently met.

In addition to potential effects on $\delta^{18}O_{precip}$ from differences in atmospheric circulation, there can also be some isotopic impact from changes in sea water $\delta^{18}O$. Sea water $\delta^{18}O$ changes between glacial and interglacial phases due to the differences in global ice volume.[72] While the isotopic composition of precipitation is predominantly driven by circulation dynamics and precipitation conditions, the isotopic composition of sea water is does also affect $\delta^{18}O_{precip}$, as sea water forms the original source of water vapor that produces clouds and eventually precipitation.[55,63] This could therefore bias temperature reconstructions, that are based on modern calibration data sets and assume consistent sea water $\delta^{18}O$. However, changes in $\delta^{18}O$ of sea water in the Late Pleistocene are relatively minor and show only small effects on $\delta^{18}O_{precip}$. The oxygen isotopic composition of seawater shows a difference of approximately 1 ‰ between the Last Glacial Maximum (LGM) and modern day.[73] This difference is of approximately the same magnitude as modern day spatial differences in $\delta^{18}O$ of sea water.[63] Additionally, the ocean water isotopic difference would be even smaller between MIS 4 and MIS 3, as the extent of glaciation was smaller in MIS 4 than in the LGM

but larger in MIS 3 than today. Additionally, other lines of evidence for$\delta^{18}O_{precip}$ during the last glacial phase in France do not suggest that changes in $\delta^{18}O$ of sea water substantially dampened $\delta^{18}O_{precip}$ changes between glacial and interglacial phases. For example, fluid inclusion $\delta^{18}O$ – which is a relatively direct proxy for $\delta^{18}O_{precip}$ values – suggests that temperature difference between the MIS 6 and MIS 5 glacial and interglacial phases are clearly reflected in $\delta^{18}O_{precip}$.[74] Similarly, 75 report changes in $\delta^{18}O_{precip}$ (reconstructed from Arvicolinae tooth enamel $\delta^{18}O$) between -14 and -6 ‰ between MIS 4 and MIS 3 in south east France. This corresponds to an isotopic difference of ca. 8 ‰, which is in agreement with expectations given the temperature differences between the two phases. This suggests an overall dominance of temperature effects on $\delta^{18}O_{precip}$ across Late Pleistocene periods of climate change with little bias from sea water $\delta^{18}O$ changes.

9. Carbon and nitrogen isotopes of bone collagen

Bones were sampled and processed for collagen extraction and carbon and nitrogen stable isotope analysis at the Max-Planck-Institute for Evolutionary Anthropology Leipzig and at the Department of Archaeology, University of Aberdeen. Bone samples were cut from larger bone pieces using a clean diamond rotary cutting disk and cleaned by removing the external surface with air abrasion. Collagen was then extracted using a modified Longin method[76] following[77] and.[78] Demineralization of bone pieces was achieved over 3-10 days using 0.5 M hydrochloric acid (HCl), which was regularly exchanged. Collagen pseudomorphs were then rinsed to neutrality using MilliQ ultrapure water and the collagen gelatinized over 48 hours in weak HCl (pH = 3) at 70 °C. The gelatinized collagen was then filtered using a 5-8 μm Ezee Filter (Elkay Laboratory Products, Basingstoke, UK) and subsequently ultra-filtered using a 30,000 kD mass cutoff. The resulting purified solution was then frozen and lyophilized. Carbon and nitrogen isotopic analyses were conducted using a Flash 2000 Organic Elemental Analyzer (Thermo Fisher Scientific, Bremen, Germany) coupled to a Delta XP ratio mass spectrometer (Thermo Fisher Scientific, Bremen, Germany) via a Conflo III interface (Thermo Fisher Scientific, Bremen, Germany). Approximately 0.5 mg of collagen was weighed into tin

capsules and introduced to the Elemental Analyzer using an AS 200S autosampler. The oxidation and reduction reactors were held at 1020 °C and 650 °C respectively. Gas separation was achieved with the GC column held at 65 °C with a carrier gas flow of 115 mL/min. All samples were analyzed in duplicate with a precision of replicate measurements of better than 0.1 ‰ on average. Carbon and nitrogen stable isotope values were two-point scale normalized to the VPDB and AIR scale respectively using IAEA-CH-6 (sucrose, $\delta^{13}C$ = -10.449 ± 0.033 ‰), IAEA-CH-7 (polyethylene, $\delta^{13}C$ = -32.151 ± 0.050 ‰), IAEA-N-1 (ammonium sulfate, $\delta^{15}N$ = 0.4 ± 0.2 ‰) and IAEA-N-2 (ammonium sulfate, $\delta^{15}N$ = 20.3 ± 0.2 ‰). Normalization was checked using NIST SRM 1577b (bovine liver, n = 4), which gave values of -21.60 ± 0.06 ‰ (1 s.d.) and 7.64 ± 0.03 ‰ (1 s.d.) for $\delta^{13}C$ and $\delta^{15}N$ respectively and in-house methionine standard (n = 14; Elemental Microanalysis, Okehampton, UK) which gave values of -30.05 ± 0.05 ‰ (1 s.d.) for $\delta^{13}C$ and -2.18 ± 0.02 ‰ (1 s.d) for $\delta^{15}N$. All carbon and nitrogen stable isotope values and standard deviations of replicates can be found in Supplementary Table S6.

10. Control for migratory behavior using $^{87}Sr/^{86}Sr$

To confirm that samples Bos/Bison did not exhibit long distance migratory behavior, seven individuals were sampled for Sr isotope analysis. For each of the seven individuals two data points from different seasons (as determined from $\delta^{18}O$) were obtained to assess whether seasonal movements were undertaken by these animals. All data points can be found in Supplementary Table S5.

All samples were prepared for $^{87}Sr/^{86}Sr$ analysis in a PicoTrace clean laboratory facility at the Department of Human Evolution, Max-Planck-Institute for Evolutionary Anthropology, Leipzig, following methods outlined in 79. Briefly, ca. 10 mg of tooth enamel were weighed into 7 mL Teflon beakers (Savillex, Eden Prairie, USA) and digested in 2 mL of 65% HNO_3 (Merck SupraPur grade, Merck, Darmstadt, Germany) for 2 hours at 120 °C and then evaporated to dryness. The resulting residue was re-dissolved in 1 mL of 3N HNO_3 at 120 °C for 1 hour. The resulting solution was loaded onto microcolumns containing a 0.5 cm bed of

cleaned and pre-conditioned 50-100 µm Sr-spec resin (Eichrom Technologies, Lisle, USA) microcolumns. All sample solutions were passed through the column step 4 times to ensure complete loading of strontium onto the resin. After matrix elution using 3 times 400 µL of 3N HNO_3, strontium was then eluted from the columns using 1.5 mL of MilliQ ultrapure water. The resulting Sr containing solution was evaporated to dryness at 120 °C and re-dissolved in 2 ml of 3 % HNO_3. Four process blanks were prepared alongside samples, in addition to aliquots of NIST SRM 1486, which served as a quality control standard. All samples were analyzed for $^{87}Sr/^{86}Sr$ using a Neptune Multi-Collector Inductively Coupled Plasma Mass Spectrometer (MC-ICPMS, Thermo Fisher Scientific, Bremen, Germany) at the CREAIT facility, Memorial University, St. John's, Canada. Data was corrected for on-peak blank intensities and normalized for instrument mass bias to $^{88}Sr/^{86}Sr$ = 8.375209 (exponential law). $^{87}Sr/^{86}Sr$ were monitored and corrected for Kr and ^{87}Rb interferences. External data normalization was conducted using the NIST SRM 987 reference material ($^{87}Sr/^{86}Sr$ accepted value = 0.710248,[80] average of measured values = 0.710290 $\pm$ 0.000012 (1 s.d., n = 9)) using a correction offset of -0.000042. Measurements of NIST SRM 1486 gave an average value of 0.70936 $\pm$ 0.0000095 (1 s.d., n = 2), which compares well with the expected value of 0.70930. Procedural blanks gave ^{88}Sr voltages of ca. 0.1 % of typical sample voltages. A lack of relationship between sample strontium concentration and $^{87}Sr/^{86}Sr$ values indicates that all samples preserve a biogenic strontium isotope ratio (Supplementary Figure S10).

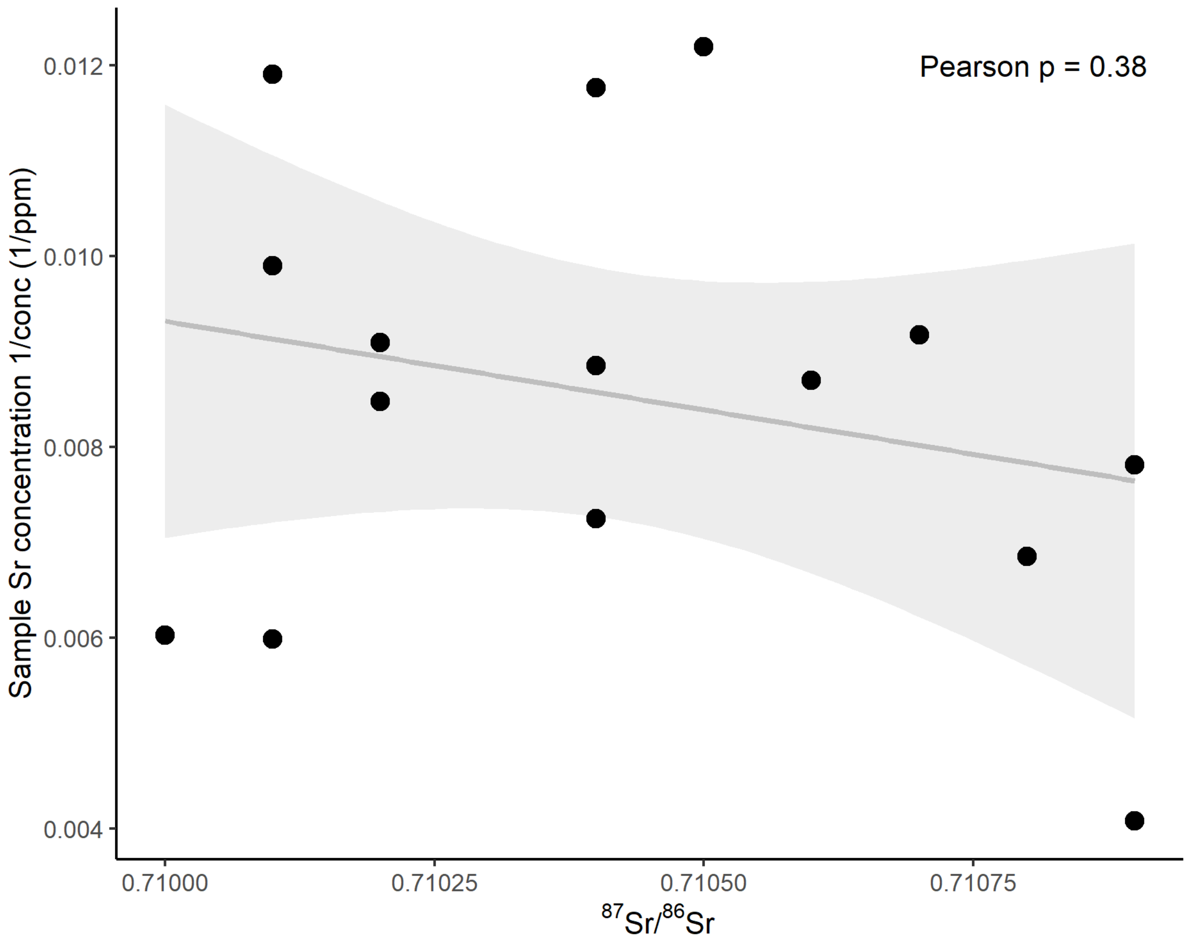

Supplementary Figure S10:Absence of a clear relationship between sample strontium concentration (1/conc) and radiogenic strontium isotope ratios in La Ferrassie Bos/Bison indicates good preservation and a lack of diagenetic alteration of strontium isotope values in these samples.

All $^{87}Sr/^{86}Sr$ measurements are consistent between seasons and across individuals (Supplementary Figure S11) and fall within the expected range of values for locally bioavailable strontium values of limestone/carbonate and sand/clay lithologies in the vicinity of La Ferrassie, as determined by local plant and snail samples as well modeled values from Sr isoscapes for France and the Dordogne region.[81,82] Matching strontium isotope ratios between summer and winter seasons indicate that *Bos/Bison* did not seasonally move to isotopically different lithologies.

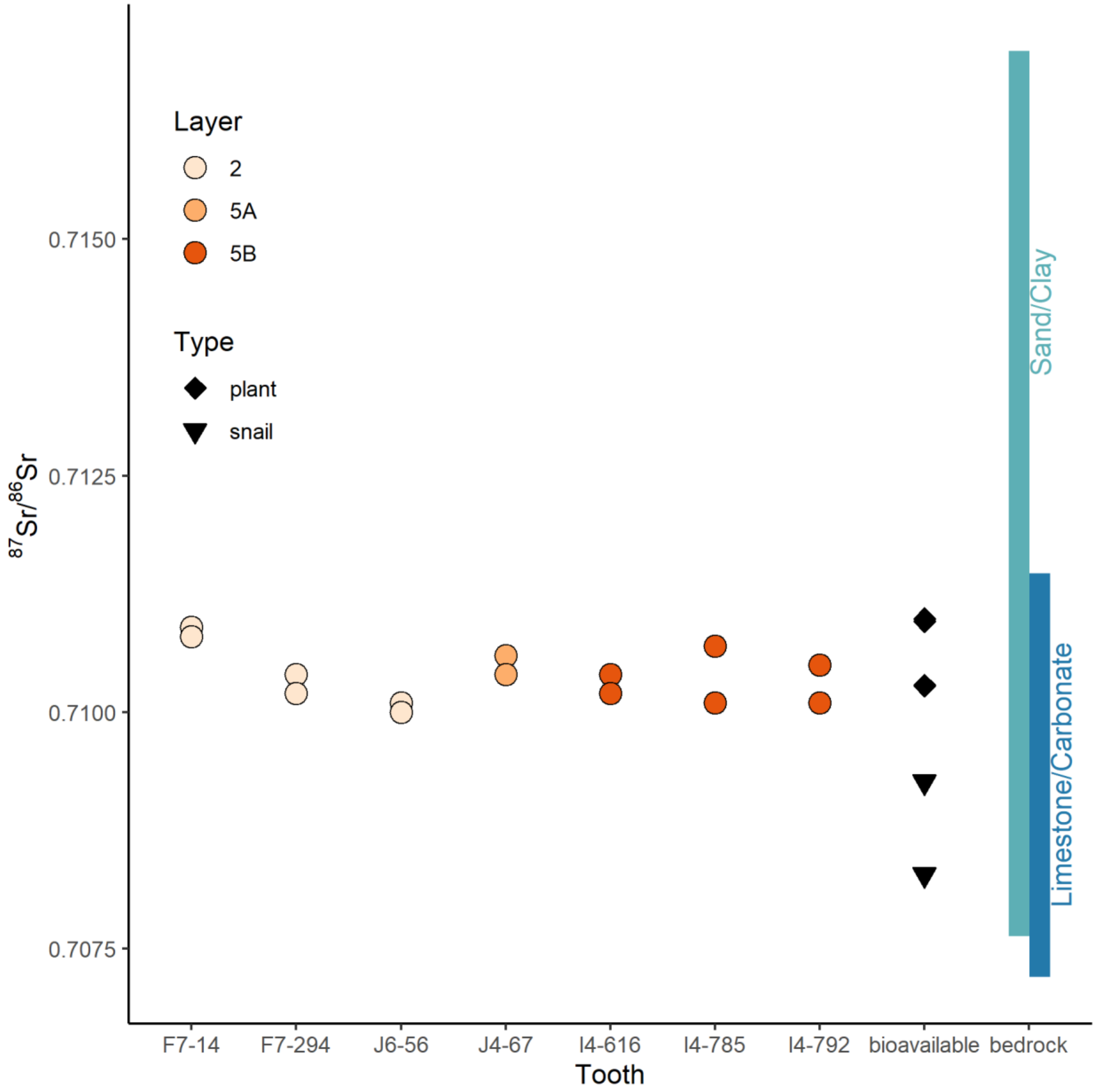

Supplementary Figure S11:$^{87/86}$Sr values of La Ferrassie *Bos/Bison* (circles) are homogenous within and across individuals and match both plant samples taken at the site (black diamonds; data from 82) and predicted values for the underlying limestone geology (blue bar) and closeby sand/clay river valleys (yellow bar), indicating absence of long distance migratory behavior. Predicted geological $^{87/86}$Sr values were taken from 81.

Bos/Bison tooth enamel ^{87}Sr/^{86}Sr values from this study match well with ^{87}Sr/^{86}Sr values for the lime stone dominated lower elevation landscapes in the southwest of the region, which range from 0.707 to 0.711 (Supplementary Figure S12, while higher elevation plateaus in the northeast are mostly characterized by substantially higher ^{87}Sr/^{86}Sr values. Small portions of the plateau are also formed of limestone lithologies and exhibit lower ^{87}Sr/^{86}Sr values between 0.709 and 0.702, more similar to the values seen in the La Ferrassie *Bos/Bison*. These

limestone plateau regions are approximately 100 km distant from the site of La Ferrassie. All *Bos/Bison* $^{87}Sr/^{86}Sr$ values studied here fall above 0.7100, and hence show no isotopic overlap with the limestone plateaus, despite a similarity in lithology between these plateaus regions and La Ferrassie. Therefore, while isotopic differences between these regions are relatively small, an origin of the hunted *Bos/Bison* from the lower elevation regions close to the site of La Ferrassie appears more parsimonious given both a better isotopic match with the low elevation regions and the large distance to the limestone plateau. The small but measurable isotopic differences between the low elevation carbonate lithologies and the limestone plateaus also suggests that animals originating from either of these areas should at least show small differences in $^{87}Sr/^{86}Sr$, which we do not detect between different layers at La Ferrassie.

We also use strontium isotope analysis to examine if *Bos/Bison* shifted between lower elevation plateaus around the site to river valley of the Dordogne or Vézère River, as this could have implications for available drinking water sources and the interpretation of oxygen stable isotope results. Strontium isotope ratios in *Bos/Bison* tooth enamel suggest that substantial use of the Dordogne valley as a habitat are unlikely. A $^{87}Sr/^{86}Sr$ isoscape of bioavailable strontium (Supplementary Figure S12) as well as $^{87}Sr/^{86}Sr$ measurements directly from the Dordogne river[59] show that the river as well as sediments deposited in the valley exhibit $^{87}Sr/^{86}Sr$ values that are higher (ranging from 0.715 to 0.720) than $^{87}Sr/^{86}Sr$ in La Ferrassie *Bos/Bison* tooth enamel (ranging from 0.7100 and 0.7109). The same is true to an extent for the Vézère River, where strontium isotope analysis of river samples and the regional isoscape also suggest slightly higher $^{87}Sr/^{86}Sr$ values (0.713 for river samples and ca 0.712 - 0.714 in the isoscape; see 59 and Supplementary Figure S12). However, the isotopic difference from the enamel samples is not as large and the Vézère River valley is relatively small, giving more opportunity for *Bos/Bison* to range across valley adjacent areas with different baseline $^{87}Sr/^{86}Sr$ values.

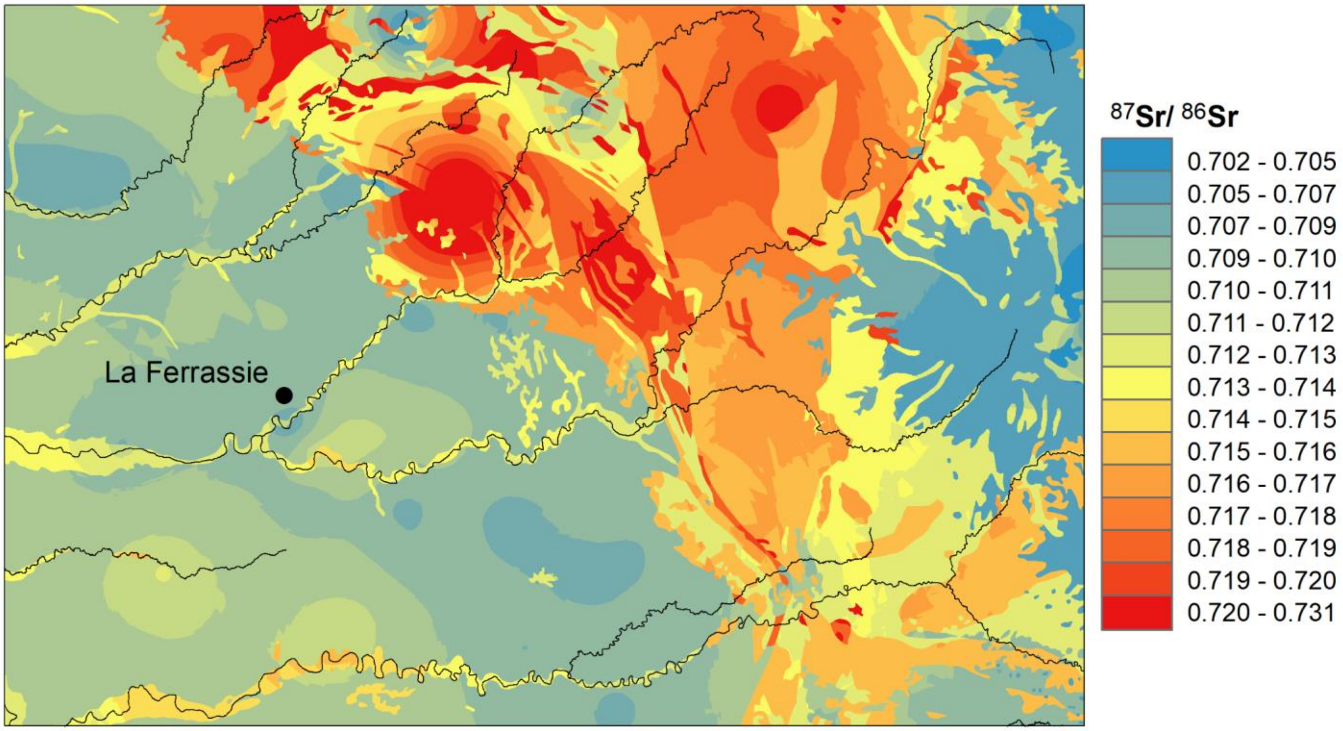

Supplementary Figure S12:Location of La Ferrassie in relation to an averaged $^{87}Sr/^{86}Sr$ isoscape of the Dordogne region based on plant and snail samples. Image by Mael Le Corre adapted from Figure 7b in 82.

11. Correlations between different isotopic systems

To investigate how different environmental and ecological changes may been connected throughout the sequence of La Ferrassie, and to explore the most likely major drivers of different isotopic systems in this case study, we conduct correlation tests of the different isotopic systems studied here (carbon, nitrogen, oxygen). A comparatively good correlation can be observed between diachronic changes in $\delta^{13}C$ and mean winter $\delta^{18}O$ (Supplementary Figure S13). Inter-layer variations in *Bos/Bison* bone collagen $\delta^{15}N$ values also broadly map onto changes seen in the other systems, however, there appears to a temporal lag compared to $\delta^{18}O$ and $\delta^{13}C$ (main text Figure 1), causing a lack of correlation between $\delta^{13}C$ and $\delta^{15}N$ overall (Supplementary Figure S14).

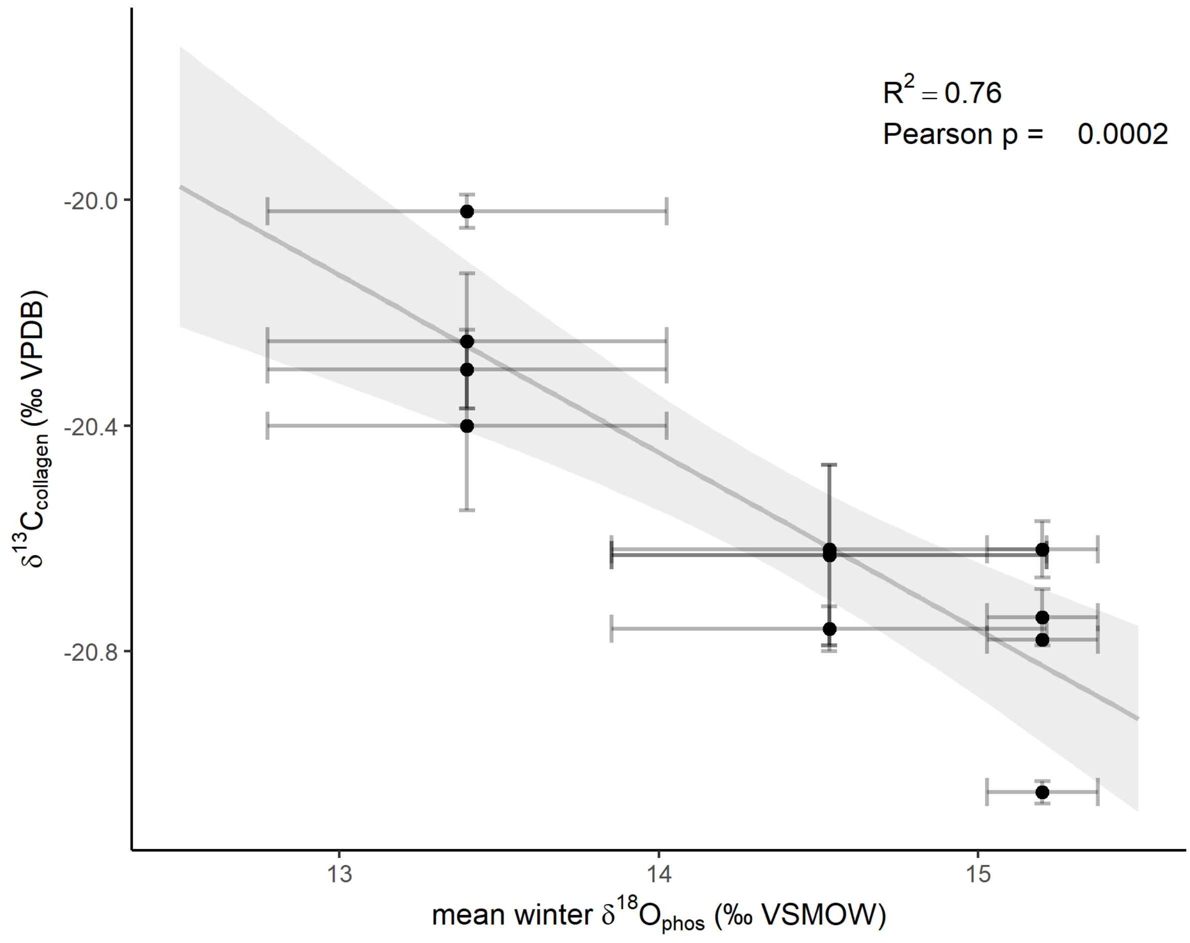

Supplementary Figure S13:Biplot of *Bos/Bison* collagen δ^{13}C and mean winter δ^{18}O of bioapatite phosphate (averaged by Layer) shows a comparatively good correlation between the tracers. This indicates that both systems robustly reflect small environmental and climatic changes though time and are at least partially driven by connected climatic and environmental influences. Error bars of δ^{18}O values represent the propagated error of the Layer mean derived by error propagation of the measurement error. δ^{13}C error bars represent 1 s.d. of replicate measurements.

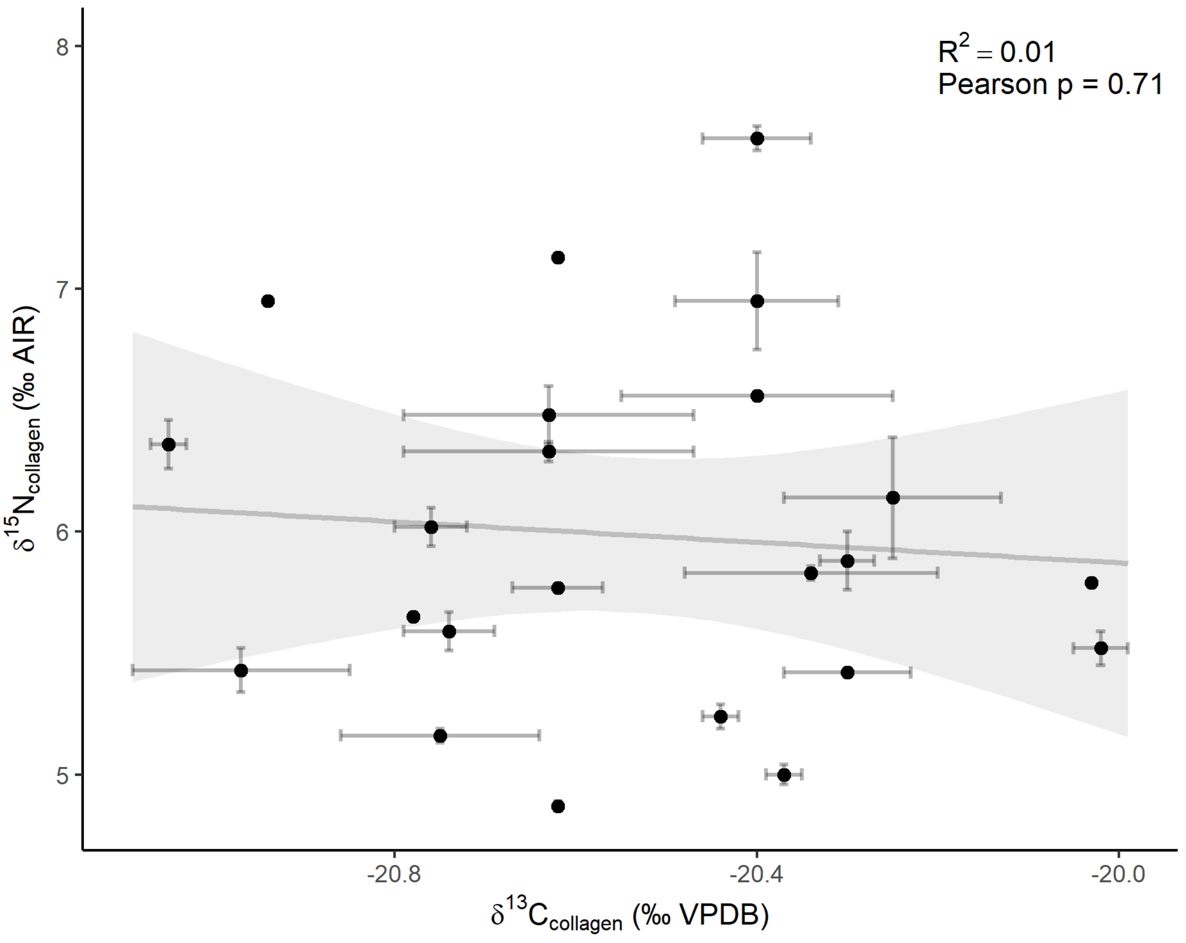

Supplementary Figure S14:While diachronic changes in *Bos/Bison* collagen δ^{13}C and δ^{15}N, show similar patterns, due to a time lag in the two tracers, no direct correlation can be observed. Error bars represent 1 s.d. of replicate measurements.

12. Sample provenance and spatial patterns in the stratigraphy

To exclude that the mismatch between climatic conditions indicated by *Bos/Bison* oxygen isotope values and the cold climate features in the sediments of Layer 2 is the result of a mixing of several occupations with differing climatic conditions within the same layer, we explore the spatial relationship between our sampled teeth, their oxygen stable isotope value, the location of reindeer bones in the sequence and the location of cold climate indicator in the layer sediments. A spatial plot of reindeer bones within the sequence reveals that they are spread evenly across Layer 2 (as well as the other studied layers) and can be found also in close proximity to *Bos/Bison* teeth that were sampled for this study

(Supplementary Figure S15). However, the number of bone fragments that could be specifically identified to *Rangifer tarandus* is low in all layers studied here, so we additionally also plot bone fragments identified as Cervid/Rangifer, which contain a mixture of large cervids and rangifer fragments, which show the same trend as the bone fragments specifically identified to *Rangifer tarandus* (Supplementary Figure S15).

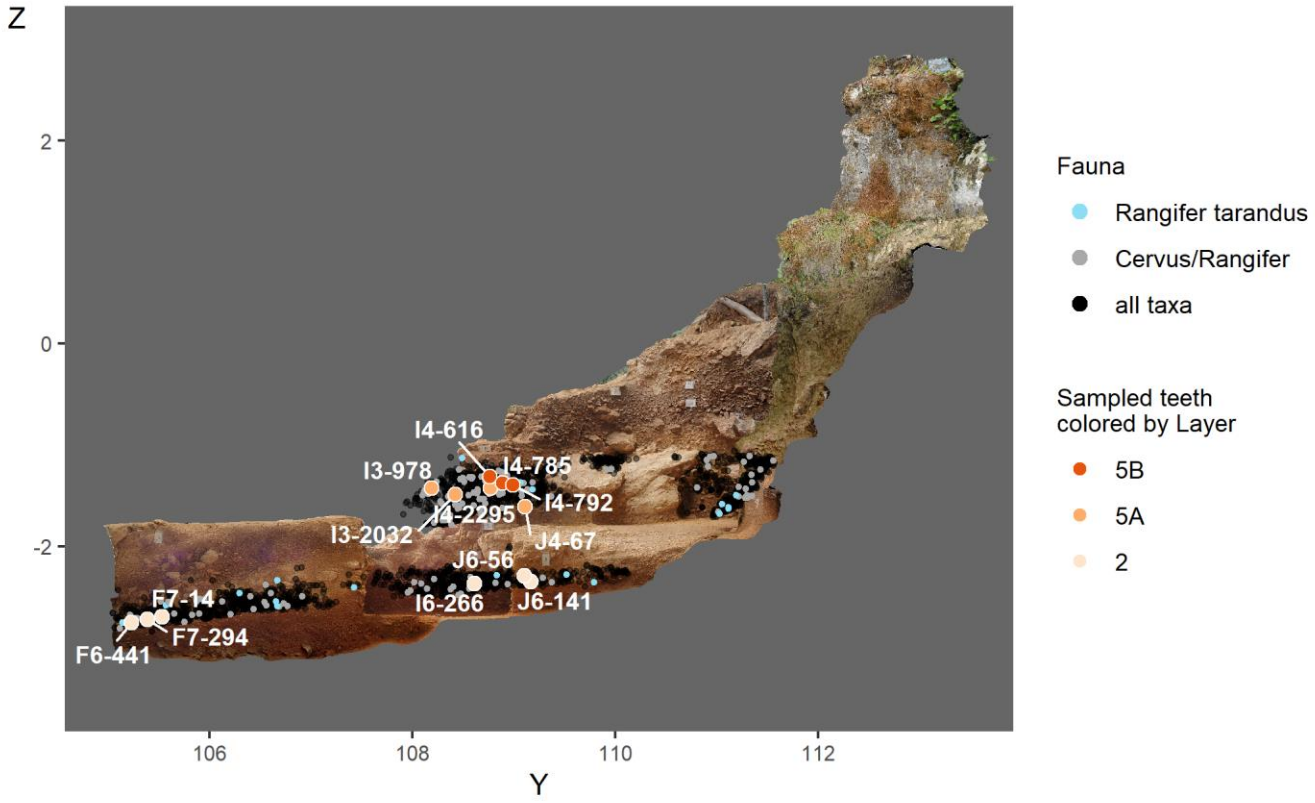

Supplementary Figure S15:Spatial plot of the find location of *Bos/Bison* teeth sampled for oxygen isotope analysis (large orange points, labelled by Sample ID, and colored by Layer) within the stratigraphic section. Shown is the west section of the Main area of the western sector, with the North wall face of the Abri on the right of the image. Plots of reindeer bone fragments (small blue circles) and of cervid/reindeer (small grey circles) in relation to all bone fragments > 2.5 cm (small black circles) shows that reindeer remains are evenly distributed throughout Layer 2 and can be found in close proximity to the sampled *Bos/Bison* remains.

In Layer 2 contains sediments near the North wall that are mainly formed by cryoclastic detachment of platy bedrock from the floor and walls and - in portions further away from the wall - sediments with ice lensing derived from the solifluction cone to the west of the excavation area. Therefore, while Layer 2 shows some spatial difference in lithologies, both

point to cold conditions during layer formation. Sampled Bos/Bison teeth were selected from sections both closer to and further away from the wall, and their location does not show a relationship with oxygen stable isotope values (Supplementary Figure S16). We therefore do not see any indication that Layer 2 constitutes a combination of several different occupations with different climatic conditions - as far as can be detected by spatial patterns within the layer. It does not appear to be the case that soliflucted material in particular represents reworked material from a warmer climatic phase, as this material is equivalent to the deposits originating from cryoturbation both in terms of presence of reindeer bones and *Bos/Bison* oxygen isotope values.

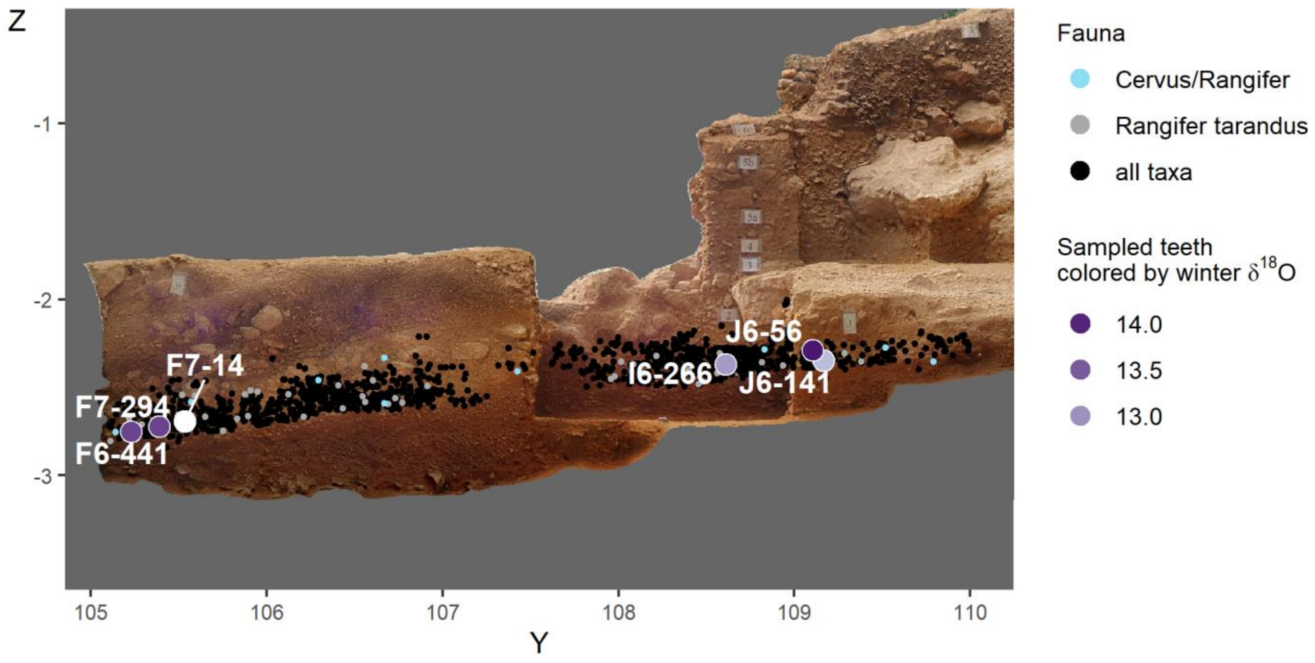

Supplementary Figure S16:In Layer 2 the spatial distribution of *Bos/Bison* tooth samples colored by oxygen stable isotope value (large purple points, labelled by Sample ID, lighter colors correspond to lower δ^{18}O values) does not show any isotopic differences between samples from different areas of Layer 2 with different extent of cold climate sedimentary features. Shown is the west section of the Main area of the western sector, with the North wall face of the Abri on the right of the image. Small black circles represent the location of bone fragments larger than 2.5 cm. Small blue circles mark the location of reindeer bone fragments and small grey circles the location of bone fragments identified as cervid/reindeer.

The seasonal oxygen isotope values of *Bos/Bison* teeth in Layer 5B show both higher $\delta^{18}O$ values similar to samples in Layer 5A and slightly lower $\delta^{18}O$ values, causing a small trend of decreasing $\delta^{18}O$ values. We use a spatial projection of *Bos/Bison* samples within Layer 5B to explore whether these tentative groups of $\delta^{18}O$ values correspond to a spatial pattern (Supplementary Figure S17). However, this does not appear to be the case as a samples with lower (I4-792) and higher (I4-785) $\delta^{18}O$ values were recovered in close proximity from the lower part of the layer, while sample I4-616 in a higher stratigraphic location again exhibits lower $\delta^{18}O$ values.

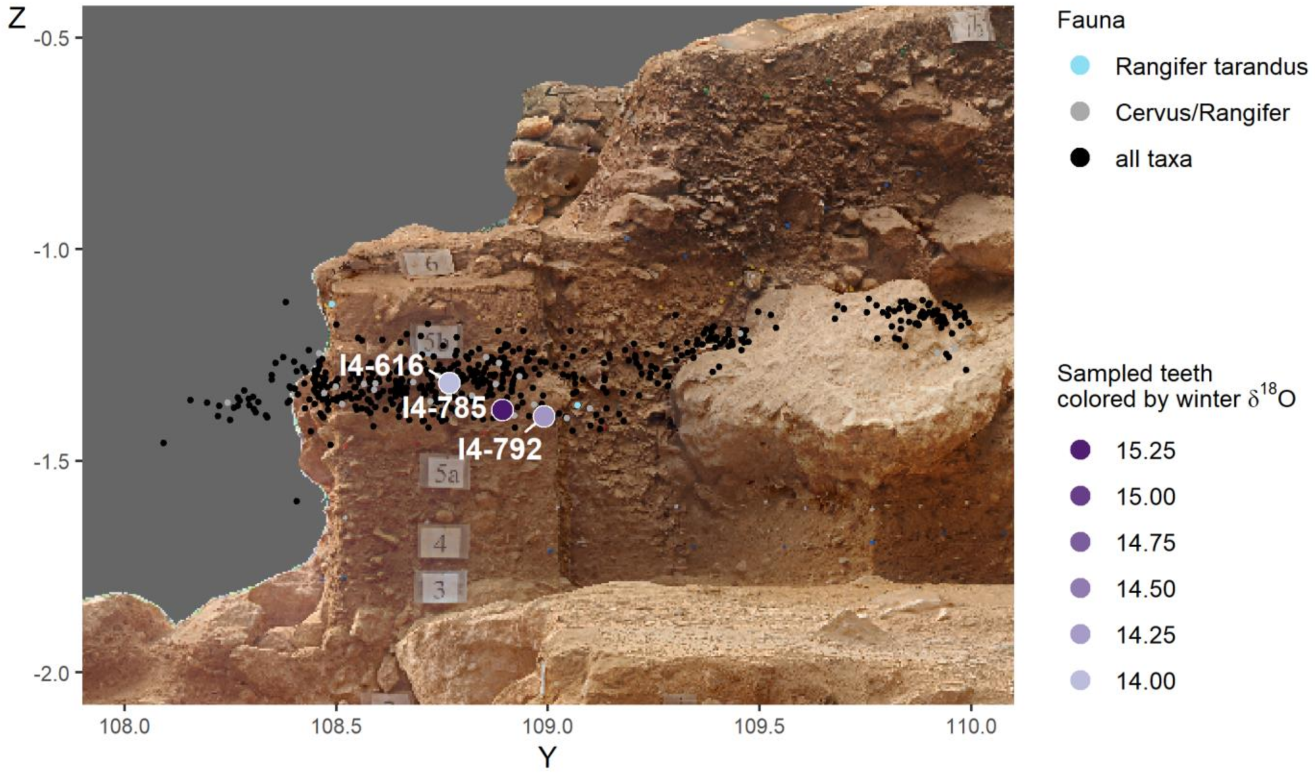

Supplementary Figure S17:In Layer 5B the spatial distribution of *Bos/Bison* tooth samples colored by oxygen stable isotope value (large purple points, labelled by Sample ID, lighter colors correspond to lower $\delta^{18}O$ values) does not show any isotopic patterns according to stratigraphic positions. Shown is the west section of the Main area of the western sector, with the North wall face of the Abri on the right of the image. Small black circles represent the location of bone fragments larger than 2.5 cm. Small blue circles mark the location of reindeer bone fragments and small grey circles the location of bone fragments identified as cervid/reindeer.

As we sample both teeth and bones from *Bos/Bison* that are not anatomically connected to each other, we explore the spatial relationship between tooth and bone samples to establish that both sample types represent comparable groups of animals and can be interpreted as evidence of climatic conditions for the same time period. For all three layer where teeth were analyzed, at least one tooth was recovered in close spatial proximity to a bone sample (Supplementary Figure S18). Additionally, bone samples originate from several different parts of each layer and should therefore not be substantially biased towards any particular group of faunal remains within any layer. The bone samples that are in close proximity to tooth samples are likely to represent the same populations as these teeth. The lack of isotopic variability within both bones and teeth then further indicates that this connection also extends to the tooth samples that are not themselves in proximity to a bone sample.

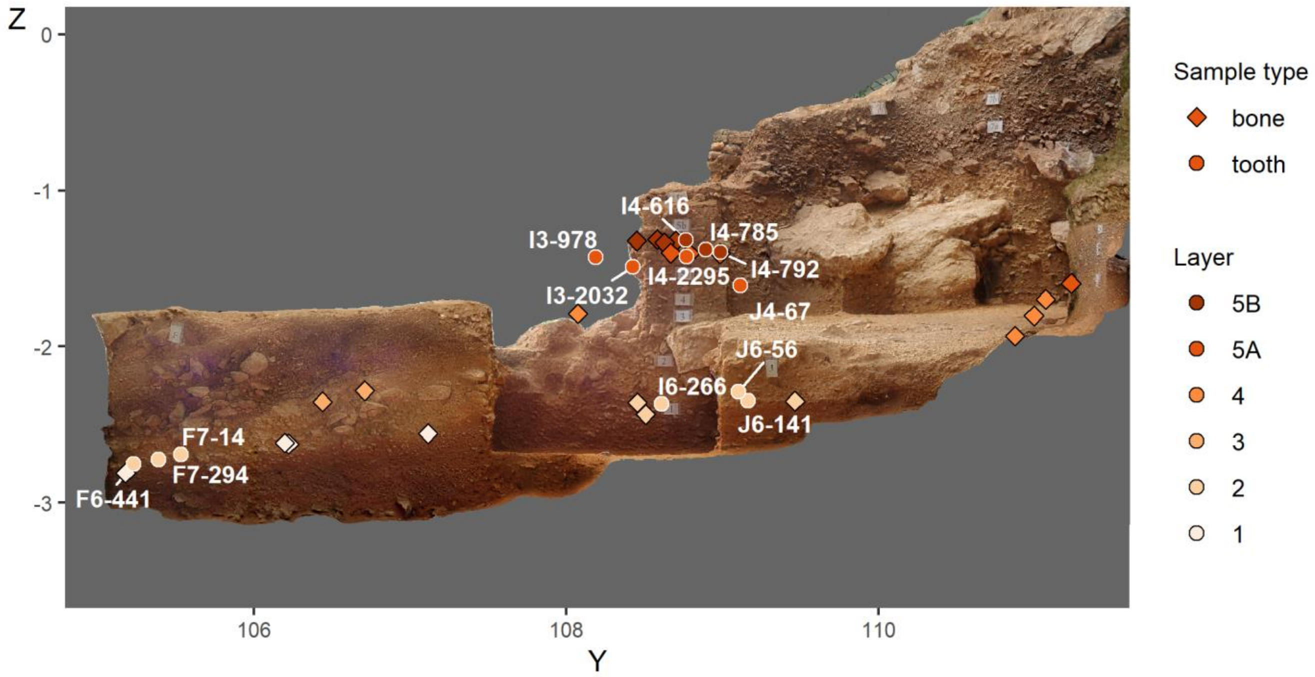

Supplementary Figure S18:Spatial projection of all teeth (circles) and bones (diamonds) sampled for stable isotope analysis show that for each layer at least one tooth sample was obtained from close proximity to a bone sample. Tooth samples are labelled with Sample IDs and all sample points are colored by layer. Shown is

the west section of the Main area of the western sector, with the North wall face of the Abri on the right of the image. Note that spatial projections of samples can plot slightly past layer boundaries that can be seen in the section orthophotograph as some samples were recovered at some distance from the section. One bone sample (F7-107; Layer 3) was excluded from this projection as it originates from a greater distance from the section and plotted far outside the layer boundaries of the section photograph.

Additionally, we plot the location of tooth samples in relation to all recorded *Bos/Bison* bones larger than 2.5 cm in Supplementary Figure S19. This plot shows that *Bos/Bison* bones are evenly spread throughout Layers 2, 5A and 5B, including all areas where teeth were obtained. We therefore overall believe that - to the extent that can be determined by spatial association - the Bos/Bison bones and teeth from La Ferrassie are sufficiently well connected to each other to assume they represent a comparable collection of animals.

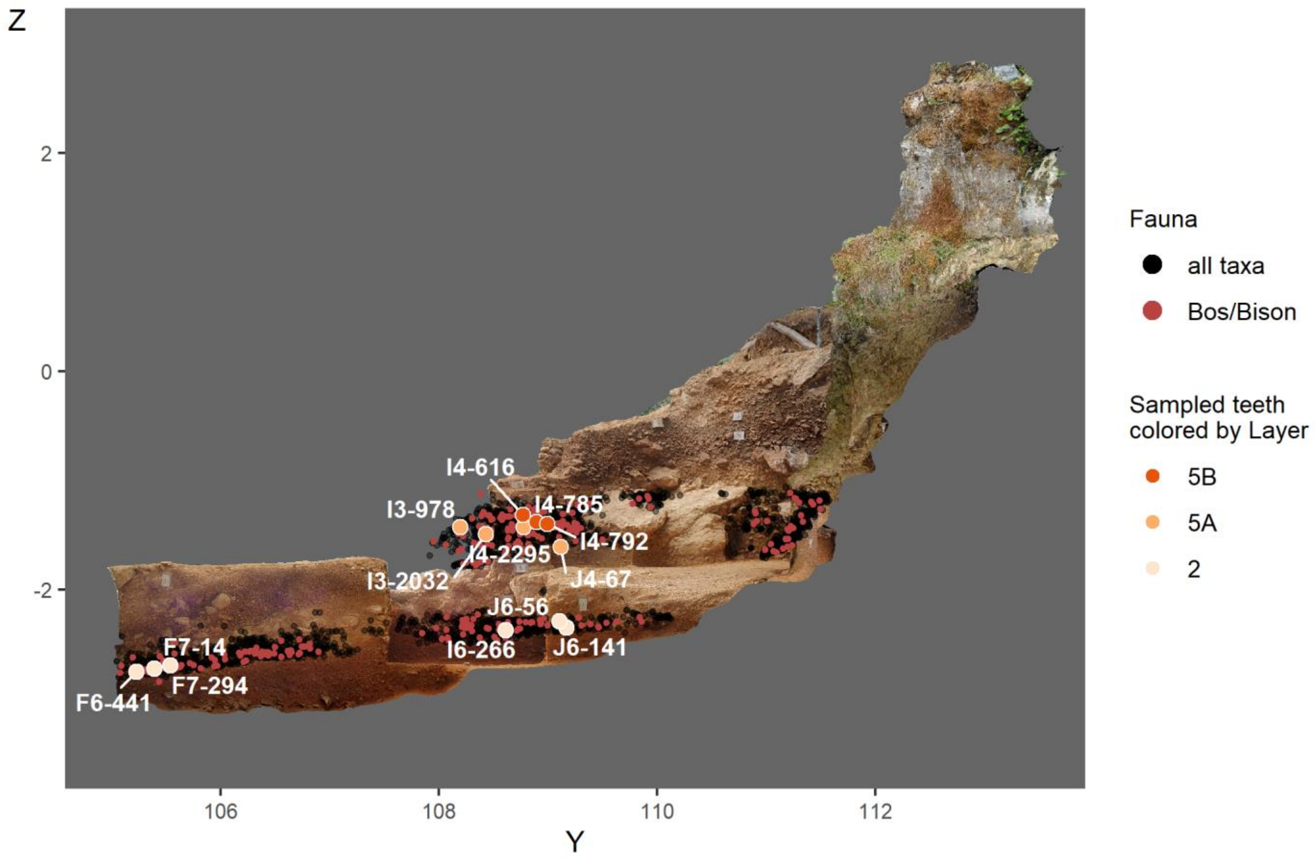

Supplementary Figure S19:Spatial plot of the find location of *Bos/Bison* teeth sampled for oxygen isotope analysis (large orange points, labelled by Sample ID, and colored by Layer) in relation to all recorded *Bos/Bison* bones > 2.5 cm (small red circles). All bone fragments > 2.5 cm (small black circles) are plotted for context in the background. Shown is the west section of the Main area of the western sector, with the North wall face of the Abri on the right of the image.

13. Software, code and data

This article, including code for all data analyses, was written in R version 4.0.2[83] on a Windows 10 operating system and the manuscript rendered using RMarkdown. All raw data as well as the RMarkdown script to reproduce the article and its analyses are available at https://osf.io/sfnb8/?view_only=622493c0f28b48c99eece196a5fe0b7b. The code for data analysis and manuscript and SI rendering makes use of the officer_0.3.14,[84] flextable_0.5.11,[85] stringr_1.4.0,[86] RColorBrewer_1.1-2,[87] ggnewscale_0.4.3,[88] ggstance_0.3.4,[89] captioner_2.2.3,[90] magick_2.4.0,[91] effsize_0.8.0,[92] lawstat_3.4,[93] cowplot_1.1.0,[94] purrr_0.3.4,[95] tidyr_1.1.2,[96] dplyr_1.0.2,[97] ggplot2_3.3.2,[98] knitr_1.29,[99] redoc_0.1.0.9000,[100] ggrepel_0.8.2,[101] gridExtra_2.3,[102] ggmap_3.0.0,[103] ratser_3.4.5,[104] tiff_0.1-5[105] packages.

14. SI References

1. Capitan, L. & Peyrony, D. Découverte d'un sixième squelette moustérien à La Ferrassie (Dordogne). *Revue Anthropologique* **31**, 382–388 (1921).

2. Capitan, L. & Peyrony, D. *Station préhistorique de la Ferrassie, commune de Savignac-du-Bugue (Dordogne)*. (E. Nourry, 1922).

3. Peyrony, D. La Ferrassie. Moustérien, Périgordien, Aurignacien. Préhistoire III. *Préhistoire* (1934).

4. Delporte, H. & Delibrias, G. *Le grand abri de la Ferrassie: fouilles 1968-1973*. (Ed. du Laboratoire de paléontologie humaine et de préhistoire, 1984).

5. Turq, A. *et al.* La Ferrassie: Rapport d'opération pour l'année 2012. (2012).

6. Guérin, G. *et al.* A multi-method luminescence dating of the Palaeolithic sequence of La Ferrassie based on new excavations adjacent to the La Ferrassie 1 and 2 skeletons. *Journal of Archaeological Science* **58**, 147–166 (2015).

7. Frouin, M. *et al.* New luminescence dating results based on polymineral fine grains from the Middle and Upper Palaeolithic site of La Ferrassie (Dordogne, SW France). *Quaternary Geochronology* **39**, 131–141 (2017).

8. Talamo, S. *et al.* The new 14C chronology for the Palaeolithic site of La Ferrassie, France: the disappearance of Neanderthals and the arrival of Homo sapiens in France. *Journal of Quaternary Science* (2020) doi:10.1002/jqs.3236.

9. Dibble, H. L., Lin, S. C., Sandgathe, D. M. & Turq, A. Assessing the Integrity of Older Archeological Collections: an Example from La Ferrassie. *Journal of Paleolithic Archaeology* **1**, 179–201 (2018).

10. Niven, L. & Martin, H. Zooarcheological Analysis of the Assemblage from the 2000–2003 Excavations. in *The middle paleolithic site of pech de l'Azé iv* 95–116 (Springer, 2018).

11. Castel, J.-C. *et al.* Neandertal subsistence strategies during the Quina Mousterian at Roc de Marsal (France). *Quaternary International* **433**, 140–156 (2017).

12. Dettman, D. L. *et al.* Seasonal stable isotope evidence for a strong Asian monsoon. *Geology* **29**, 31–34 (2001).

13. Tütken, T., Vennemann, T. W., Janz, H. & Heizmann, E. P. J. Palaeoenvironment and palaeoclimate of the Middle Miocene lake in the Steinheim basin, SW Germany: A reconstruction from C, O, and Sr isotopes of fossil remains. *Palaeogeography, Palaeoclimatology, Palaeoecology* **241**, 457–491 (2006).

14. Brand, W. A. *et al.* Comprehensive inter-laboratory calibration of reference materials for δ18O versus VSMOW using various on-line high-temperature conversion techniques. *Rapid Communications in Mass Spectrometry* **23**, 999–1019 (2009).

15. Pucéat, E. *et al.* Revised phosphate-water fractionation equation reassessing paleotemperatures derived from biogenic apatite. *Earth and Planetary Science Letters* **298**, 135–142 (2010).

16. Clark, I. D. & Fritz, P. *Environmental isotopes in hydrogeology*. 328 (Lewis Publishers, 1997).

17. Rozanski, K., Araguás-Araguás, L. & Gonfiantini, R. Isotopic patterns in modern global precipitation. *Climate change in Continental Isotopic Records* **78**, 1–36 (1993).

18. Müller, S., Stumpp, C., Sørensen, J. H. & Jessen, S. Spatiotemporal variation of stable isotopic composition in precipitation: Post-condensational effects in a humid area. *Hydrological Processes* **31**, 3146–3159 (2017).

19. Hoppe, K. A. Correlation between the oxygen isotope ratio of North American bison teeth and local waters: Implication for paleoclimatic reconstructions. *Earth and Planetary Science Letters* **244**, 408–417 (2006).

20. Kohn, M. J., Schoeninger, M. J. & Valley, J. W. Herbivore tooth oxygen isotope compositions: Effects of diet and physiology. *Geochimica et Cosmochimica Acta* **60**, 3889–3896 (1996).

21. Fricke, H. C. & O'Neil, J. R. Inter- and intra-tooth variation in the oxygen isotope composition of mammalian tooth enamel phosphate: Implications for palaeoclimatological

and palaeobiological research. *Palaeogeography, Palaeoclimatology, Palaeoecology* **126**, 91–99 (1996).

22. Fricke, H. C., Clyde, W. C. & O'Neil, J. R. Intra-tooth variations in d18O (PO4) of mammalian tooth enamel as a record of seasonal variations in continental climate variables. *Geochimica et Cosmochimica Acta* **62**, 1839–1850 (1998).

23. Bryant, J. D., Koch, P. L., Froelich, P. N., Showers, W. J. & Genna, B. J. Oxygen isotope partitioning between phosphate and carbonate in mammalian apatite. *Geochimica et Cosmochimica Acta* **60**, 5145–5148 (1996).

24. Luz, B., Kolodny, Y. & Horowitz, M. Fractionation of oxygen isotopes between mammalian bone-phosphate and environmental drinking water. *Geochimica et Cosmochimica Acta* **48**, 1689–1693 (1984).

25. Longinelli, A. Oxygen isotopes in mammal bone phosphate: A new tool for paleohydrological and paleoclimatological research? *Geochimica et Cosmochimica Acta* **48**, 385–390 (1984).

26. Iacumin, P., Bocherens, H., Mariotti, a. & Longinelli, A. Oxygen isotope analyses of co-existing carbonate and phosphate in biogenic apatite: a way to monitor diagenetic alteration of bone phosphate? *Earth and Planetary Science Letters* **142**, 1–6 (1996).

27. Balasse, M. Potential biases in sampling design and interpretation of intra-tooth isotope analysis. *International Journal of Osteoarchaeology* **13**, 3–10 (2003).

28. Zazzo, A., Balasse, M. & Patterson, W. P. High-resolution δ13C intratooth profiles in bovine enamel: Implications for mineralization pattern and isotopic attenuation. *Geochimica et Cosmochimica Acta* **69**, 3631–3642 (2005).

29. Kierdorf, H., Kierdorf, U., Frölich, K. & Witzel, C. Lines of evidence-incremental markings in molar enamel of Soay sheep as revealed by a fluorochrome labeling and backscattered electron imaging study. *PloS one* **8**, e74597 (2013).

30. Suga, S. Progressive mineralization pattern of developing enamel during the maturation stage. *Journal of dental research* **61**, 1532–42 (1982).

31. Green, D. R. *et al.* Synchrotron imaging and Markov Chain Monte Carlo reveal tooth mineralization patterns. *PLoS ONE* **12**, 1–16 (2017).

32. Moss-Salentijn, L., Moss, M. L. & Yuan, M. S. T. The ontogeny of mammalian enamel. in *Tooth enamel microstructure* (eds. Koeningswald, W. V. & Sander, P. M.) 5–30 (A.A. Balkema, 1997).

33. Trayler, R. B. & Kohn, M. J. Tooth enamel maturation reequilibrates oxygen isotope compositions and supports simple sampling methods. *Geochimica et Cosmochimica Acta* **198**, 32–47 (2016).

34. Passey, B. H. & Cerling, T. E. Tooth enamel mineralization in ungulates: implications for recovering a primary isotopic time-series. *Geochimica et Cosmochimica Acta* **66**, 3225–3234 (2002).

35. Hoppe, K. A., Stover, S. M., Pascoe, J. R. & Amundson, R. Tooth enamel biomineralization in extant horses: Implications for isotopic microsampling. *Palaeogeography, Palaeoclimatology, Palaeoecology* **206**, 355–365 (2004).

36. Blumenthal, S. A. *et al.* Stable isotope time-series in mammalian teeth: In situ δ18O from the innermost enamel layer. *Geochimica et Cosmochimica Acta* **124**, 223–236 (2014).

37. Balasse, M. Reconstructing dietary and environmental history from enamel isotopic analysis: time resolution of intra-tooth sequential sampling. *International Journal of Osteoarchaeology* **12**, 155–165 (2002).

38. Kohn, M. J. Comment: Tooth enamel mineralization in ungulates: Implications for recovering a primary isotopic time-series, by B. H. Passey and T. E. Cerling (2002). *Geochimica et Cosmochimica Acta* **68**, 403–405 (2004).

39. Passey, B. H. *et al.* Inverse methods for estimating primary input signals from time-averaged isotope profiles. *Geochimica et Cosmochimica Acta* **69**, 4101–4116 (2005).

40. Bendrey, R., Vella, D., Zazzo, A., Balasse, M. & Lepetz, S. Exponentially decreasing tooth growth rate in horse teeth: implications for isotopic analyses. *Archaeometry* **57**, 1104–1124 (2015).

41. Green, D. R., Olack, G. & Colman, A. S. Determinants of blood water δ18O variation in a population of experimental sheep: Implications for paleoclimate reconstruction. *Chemical Geology* **485**, 32–43 (2018).

42. Blumenthal, S. A., Cerling, T. E., Smiley, T. M., Badgley, C. E. & Plummer, T. W. Isotopic records of climate seasonality in equid teeth. *Geochimica et Cosmochimica Acta* **260**, 329–348 (2019).

43. Zazzo, A. *et al.* A refined sampling strategy for intra-tooth stable isotope analysis of mammalian enamel. *Geochimica et Cosmochimica Acta* **84**, 1–13 (2012).

44. Zazzo, A. *et al.* The isotope record of short- and long-term dietary changes in sheep tooth enamel: Implications for quantitative reconstruction of paleodiets. *Geochimica et Cosmochimica Acta* **74**, 3571–3586 (2010).

45. Tütken, T., Furrer, H. & Walter Vennemann, T. Stable isotope compositions of mammoth teeth from Niederweningen, Switzerland: Implications for the Late Pleistocene climate, environment, and diet. *Quaternary International* **164-165**, 139–150 (2007).

46. Pryor, A. J. E., Stevens, R. E., O'Connell, T. C. & Lister, J. R. Quantification and propagation of errors when converting vertebrate biomineral oxygen isotope data to temperature for

palaeoclimate reconstruction. *Palaeogeography, Palaeoclimatology, Palaeoecology* **412**, 99–107 (2014).

47. Skrzypek, G., Sadler, R. & Wiśniewski, A. Reassessment of recommendations for processing mammal phosphate δ18O data for paleotemperature reconstruction. *Palaeogeography, Palaeoclimatology, Palaeoecology* **446**, 162–167 (2016).

48. Rozanski, K., Araguás-Araguás, L. & Gonfiantini, R. Relation Between Long-Term Trends of Oxygen-18 Isotope Composition of Precipitation and. *Source: Science, New Series* **258**, 981–985 (1992).

49. Arppe, L. M. & Karhu, J. A. Oxygen isotope values of precipitation and the thermal climate in Europe during the middle to late Weichselian ice age. *Quaternary Science Reviews* **29**, 1263–1275 (2010).

50. D'Angela, D. & Longinelli, A. Oxygen isotopes in living mammal's bone phosphate: Further results. *Chemical Geology: Isotope Geoscience section* **86**, 75–82 (1990).

51. Maloiy, G. M. O. Water metabolism of East African ruminants in arid and semi-arid regions. *Zeitschrift für Tierzüchtung und Züchtungsbiologie* **90**, 219–228 (1973).

52. Arias, R. A. & Mader, T. L. Environmental factors affecting daily water intake on cattle finished in feedlots. *Journal of Animal Science* **89**, 245–251 (2011).

53. McHugh, T. Social behavior of the American buffalo (Bison bison bison). *Zoologica* **43**, 1–40 (1958).

54. Winchester, C. F. & Morris, M. J. Water Intake Rates of Cattle. *Journal of Animal Science* **15**, 722–740 (1956).

55. Gat, J. R. *Isotope Hydrology: A Study of the Water Cycle*. vol. 6 189 (Imperila College Press, 2010).

56. Darling, W. G., Bath, A. H. & Talbot, J. C. The O & H stable isotopic composition of fresh waters in the British Isles. 2. Surface waters and groundwater. *Hydrology and Earth System Sciences* **7**, 183–195 (2003).

57. Halder, J., Terzer, S., Wassenaar, L. I., Araguás-Araguás, L. & Aggarwal, P. K. The Global Network of Isotopes in Rivers (GNIR): Integration of water isotopes in watershed observation and riverine research. *Hydrology and Earth System Sciences* **19**, 3419–3431 (2015).

58. Gonfiantini, R. Environmental isotopes in lake studies. *Handbook of Environmental Isotope Geochemistry ; The Terrestrial Environment* 113–168 (1986).

59. Parra, M., Castaing, P., Jouanneau, J.-M., Grousset, F. & Latouche, C. Nd–Sr isotopic composition of present-day sediments from the Gironde Estuary, its draining basins and the WestGironde mud patch (SW France). *Continental shelf research* **19**, 135–150 (1999).

60. Bentley, R. A. Strontium Isotopes from the Earth to the Archaeological Skeleton: A Review. *Journal of Archaeological Method and Theory* **13**, 135–187 (2006).

61. Bowen, G. J. OIPC: The online isotopes in precipitation calculator, version 3.1. (2020).

62. Stumpp, C., Klaus, J. & Stichler, W. Analysis of long-term stable isotopic composition in German precipitation. *Journal of Hydrology* **517**, 351–361 (2014).

63. Dansgaard, W. Stable isotopes in precipitation. *Tellus* **16**, 436–468 (1964).

64. Kohn, M. J. & Welker, J. M. On the temperature correlation of δ18O in modern precipitation. *Earth and Planetary Science Letters* **231**, 87–96 (2005).

65. Aggarwal, P. K. *et al.* Stable isotopes in global precipitation: A unified interpretation based on atmospheric moisture residence time. *Geophysical Research Letters* **39**, 1–6 (2012).

66. Akers, P. D., Welker, J. M. & Brook, G. A. Reassessing the role of temperature in precipitation oxygen isotopes across the eastern and central United States through weekly precipitation-day data. *Water Resources Research* **53**, 7644–7661 (2017).

67. Bowen, G. J. & West, J. B. Isotope Landscapes for Terrestrial Migration Research. in *Tracking animal migration with stable isotopes* (eds. Hobson, K. A. & Wassenaar, L. I.) vol. 2 79–105 (Elsevier, 2008).

68. Schmidt, G. A., Hoffmann, G., Shindell, D. T. & Hu, Y. Modeling atmospheric stable water isotopes and the potential for constraining cloud processes and stratosphere-troposphere water exchange. *Journal of Geophysical Research Atmospheres* **110**, 1–15 (2005).

69. Rozanski, K. Deuterium and oxygen-18 in European groundwaters — Links to atmospheric circulation in the past. *Chemical Geology: Isotope Geoscience section* **52**, 349–363 (1985).

70. Zuber, A., Weise, S. M., Motyka, J., Osenbrück, K. & Rózański, K. Age and flow pattern of groundwater in a Jurassic limestone aquifer and related Tertiary sands derived from combined isotope, noble gas and chemical data. *Journal of Hydrology* **286**, 87–112 (2004).

71. Kaspar, F., Kühl, N., Cubasch, U. & Litt, T. A model-data comparison of European temperatures in the Eemian interglacial. *Geophysical Research Letters* **32**, 1–5 (2005).

72. Shackleton, N. Oxygen isotope analyses and Pleistocene temperatures re-assessed. *Nature* **215**, 15–17 (1967).

73. Schrag, D. P. *et al.* The oxygen isotopic composition of seawater during the Last Glacial Maximum. *Quaternary Science Reviews* **21**, 331–342 (2002).

74. Wainer, K. *et al.* Speleothem record of the last 180 ka in Villars cave (SW France): Investigation of a large δ18O shift between MIS6 and MIS5. *Quaternary Science Reviews* **30**, 130–146 (2011).

75. Navarro, N., Lécuyer, C., Montuire, S., Langlois, C. & Martineau, F. Oxygen isotope compositions of phosphate from arvicoline teeth and Quaternary climatic changes, Gigny, French Jura. *Quaternary Research* **62**, 172–182 (2004).

76. Longin, R. New method of collagen extraction for radiocarbon dating. *Nature* **230**, 241 (1971).

77. Collins, M. J. & Galley, P. Towards an optimal method of archaeological collagen extraction: the influence of pH and grinding. *Ancient Biomolecules* **2**, 209–223 (1998).

78. Brown, T. A., Nelson, D. E., Vogel, J. S. & Southon, J. R. Improved collagen extraction by modified Longin method. *Radiocarbon* **30**, 171–177 (1988).

79. Copeland, S. R. *et al.* Strontium isotope ratios (87Sr/86Sr) of tooth enamel: a comparison of solution and laser ablation multicollector inductively coupled plasma mass spectrometry methods. *Rapid Communications in Mass Spectrometry* **22**, 3187–3194 (2008).

80. Avanzinelli, R. *et al.* High precision Sr, Nd, and Pb isotopic analyses using the new generation thermal ionisation mass spectrometer thermofinnigan triton-Ti. *Periodico di Mineralogia* **75**, 147–166 (2005).

81. Willmes, M. *et al.* Mapping of bioavailable strontium isotope ratios in France for archaeological provenance studies. *Applied Geochemistry* **90**, 75–86 (2018).

82. Britton, K. *et al.* Sampling plants and malacofauna in 87Sr/86Sr bioavailability studies: implications for isoscape mapping and reconstructing of past mobility patterns. *in review in Frontiers in Ecology and Evolution*.

83. R Core Team. *R: A Language and Environment for Statistical Computing*. (R Foundation for Statistical Computing, 2020).

84. Gohel, D. *officer: Manipulation of Microsoft Word and PowerPoint Documents*. (2020).

85. Gohel, D. *flextable: Functions for Tabular Reporting*. (2020).

86. Wickham, H. *stringr: Simple, Consistent Wrappers for Common String Operations*. (2019).

87. Neuwirth, E. *RColorBrewer: ColorBrewer Palettes*. (2014).

88. Campitelli, E. *ggnewscale: Multiple Fill and Colour Scales in 'ggplot2'*. (2020).

89. Henry, L., Wickham, H. & Chang, W. *ggstance: Horizontal 'ggplot2' Components*. (2019).

90. Alathea, L. *captioner: Numbers Figures and Creates Simple Captions*. (2015).

91. Ooms, J. *magick: Advanced Graphics and Image-Processing in R*. (2020).

92. Torchiano, M. *effsize: Efficient Effect Size Computation*. (2019). doi:10.5281/zenodo.1480624.

93. Gastwirth, J. L. *et al. lawstat: Tools for Biostatistics, Public Policy, and Law*. (2019).

94. Wilke, C. O. *cowplot: Streamlined Plot Theme and Plot Annotations for 'ggplot2'*. (2019).

95. Henry, L. & Wickham, H. *purrr: Functional Programming Tools*. (2019).

96. Wickham, H. & Henry, L. *tidyr: Tidy Messy Data*. (2019).

97. Wickham, H., François, R., Henry, L. & Müller, K. *dplyr: A Grammar of Data Manipulation*. (2019).

98. Wickham, H. *ggplot2: Elegant Graphics for Data Analysis*. (Springer New York, 2016).

99. Xie, Y. knitr: a comprehensive tool for reproducible research in R. *Implement Reprod Res* **1**, 20 (2014).

100. Ross, N. *redoc: Reversible Reproducible Documents*. (2019).

101. Slowikowski, K. *ggrepel: Automatically Position Non-Overlapping Text Labels with 'ggplot2'*. (2020).

102. Auguie, B. *gridExtra: Miscellaneous Functions for "Grid" Graphics*. (2017).

103. Kahle, D. & Wickham, H. ggmap: Spatial Visualization with ggplot2. *The R Journal* **5**, 144–161 (2013).

104. Hijmans, R. J. *raster: Geographic Data Analysis and Modeling*. (2020).

105. Urbanek, S. *tiff: Read and write TIFF images*. (2013).

15. SI Tables

Supplementary Table S2: List of *Bos/Bison* teeth sequentially sampled for stable isotope analysis with stratigraphic positions and tooth wear information. Sup. - maxillary, inf. - mandibular, sin. - left, dex. - right.

Findnumber	Layer	Tooth position	Crown height (mm)	Tooth wear	# sequential samples
J6-56	2	M3 sup. sin.	36.4	moderate	12
J6-141	2	M3 sup. dex.	31.0	moderate/heavy	10
I6-266	2	M3 sup. sin.	38.5	moderate/heavy	13
F6-441	2	P4 sup. sin.	12.0	moderate/heavy	6
F7-14	2	P4 sup. sin.	21.1	moderate	12
F7-294	2	M3 inf. sin.	54.5	light	22
J4-67	5A	M3 inf. sin.	56.7	light	16
I3-978	5A	M3 sup. dex.	37.7	moderate	13
I4-2295	5A	M2 inf. dex.	NA	very light	19
I3-2032	5A	M3 inf. dex.	31.7	moderate/heavy	12
I4-792	5B	M3 inf. sin.	43.4	moderate/heavy	14
I4-785	5B	M3 inf. dex.	54.3	very light	17
I4-616	5B	M3 sup. dex.	37.8	moderate	12

Supplementary Table S3: Cohen's d effect sizes for comparison of oxygen isotope values across layers.

Comparison	Cohen's d
Layer 2 vs 5A Winter	-3.46
Layer 2 vs 5B Winter	-1.76
Layer 5A vs 5B Winter	1.34
Layer 2 vs 5A Summer	0.32
Layer 2 vs 5B Summer	1.12
Layer 5A vs 5B Summer	1.14
Layer 2 vs 5A MAT	-1.78
Layer 2 vs 5B MAT	0.31
Layer 5A vs 5B MAT	1.19

Supplementary Table S4: Oxygen isotope values and standard deviations of replicate measurements (commonly triplicate) for all sequential tooth enamel samples presented in this study.

Sample ID	Tooth	Layer	mm from ERJ	$\delta^{18}O$	SD
I4-792.A	I4-792	5B	40.64	15.0	0.2
I4-792.B	I4-792	5B	37.68	14.8	0.4
I4-792.C	I4-792	5B	35.15	14.9	0.1
I4-792.D	I4-792	5B	31.76	15.4	0.2
I4-792.E	I4-792	5B	28.41	16.5	0.2
I4-792.F	I4-792	5B	25.07	16.6	0.3
I4-792.G	I4-792	5B	22.27	16.9	0.0

Sample ID	Tooth	Layer	mm from ERJ	$\delta^{18}O$	SD
I4-792.H	I4-792	5B	19.24	16.8	0.3
I4-792.I	I4-792	5B	16.51	16.9	0.5
I4-792.J	I4-792	5B	13.58	16.6	0.3
I4-792.K	I4-792	5B	10.42	15.4	0.3
I4-792.L	I4-792	5B	7.51	15.1	0.2
I4-792.M	I4-792	5B	4.40	14.4	0.4
I4-792.N	I4-792	5B	1.71	14.3	0.3
I3-978.A	I3-978	5A	39.60	16.1	0.2
I3-978.B	I3-978	5A	36.06	15.4	0.1
I3-978.C	I3-978	5A	32.51	16.3	0.4
I3-978.D	I3-978	5A	29.79	17.2	0.3
I3-978.E	I3-978	5A	26.16	17.7	0.2
I3-978.F	I3-978	5A	23.94	18.0	0.3
I3-978.G	I3-978	5A	20.56	18.6	0.3
I3-978.H	I3-978	5A	17.30	18.7	0.4
I3-978.I	I3-978	5A	14.56	18.9	0.1
I3-978.J	I3-978	5A	11.48	18.9	0.2
I3-978.K	I3-978	5A	8.66	18.8	0.3
I3-978.L	I3-978	5A	5.64	18.2	0.3
I3-978.M	I3-978	5A	2.62	17.4	0.3
I4-616.A	I4-616	5B	35.62	16.1	0.3
I4-616.B	I4-616	5B	32.44	16.2	0.2
I4-616.C	I4-616	5B	28.99	16.4	0.1
I4-616.D	I4-616	5B	26.35	16.2	0.4
I4-616.E	I4-616	5B	23.17	15.9	0.1
I4-616.F	I4-616	5B	20.12	15.4	0.2
I4-616.G	I4-616	5B	16.63	14.8	0.3
I4-616.H	I4-616	5B	13.53	14.0	0.1
I4-616.I	I4-616	5B	10.61	14.0	0.1
I4-616.J	I4-616	5B	6.99	14.4	0.4
I4-616.K	I4-616	5B	4.32	15.6	0.2
I4-616.L	I4-616	5B	2.23	17.0	0.0
I4-785.A	I4-785	5B	50.21	17.2	0.3
I4-785.B	I4-785	5B	47.20	16.7	0.1
I4-785.C	I4-785	5B	43.84	16.1	0.1
I4-785.D	I4-785	5B	40.40	15.7	0.2
I4-785.E	I4-785	5B	37.37	15.3	0.1
I4-785.F	I4-785	5B	34.38	15.6	0.1

Sample ID	Tooth	Layer	mm from ERJ	$\delta^{18}O$	SD
I4-785.G	I4-785	5B	30.77	15.6	0.3
I4-785.H	I4-785	5B	28.14	15.9	0.1
I4-785.I	I4-785	5B	25.24	16.0	0.1
I4-785.J	I4-785	5B	22.22	16.8	0.1
I4-785.K	I4-785	5B	19.35	17.3	0.1
I4-785.L	I4-785	5B	16.35	17.8	0.3
I4-785.M	I4-785	5B	13.68	18.2	0.2
I4-785.N	I4-785	5B	10.73	18.1	0.2
I4-785.O	I4-785	5B	7.94	18.5	0.3
I4-785.P	I4-785	5B	5.13	18.0	0.2
I4-785.Q	I4-785	5B	2.52	17.9	0.2
J4-67.A	J4-67	5A	48.89	17.3	0.1
J4-67.B	J4-67	5A	45.54	16.5	0.3
J4-67.C	J4-67	5A	41.93	16.6	0.1
J4-67.D	J4-67	5A	38.89	16.2	0.1
J4-67.E	J4-67	5A	36.10	15.5	0.2
J4-67.F	J4-67	5A	33.01	15.1	0.5
J4-67.G	J4-67	5A	30.12	15.4	0.1
J4-67.H	J4-67	5A	26.83	15.4	0.3
J4-67.I	J4-67	5A	23.68	16.0	0.1
J4-67.J	J4-67	5A	20.60	16.4	0.0
J4-67.K	J4-67	5A	17.19	16.8	0.2
J4-67.L	J4-67	5A	14.15	17.5	0.3
J4-67.M	J4-67	5A	11.15	17.7	0.1
J4-67.N	J4-67	5A	7.61	17.7	0.2
J4-67.O	J4-67	5A	4.53	16.6	0.4
J4-67.P	J4-67	5A	1.72	15.9	0.2
J6-56.A	J6-56	2	35.18	16.7	0.1
J6-56.B	J6-56	2	31.88	17.4	0.3
J6-56.C	J6-56	2	28.87	17.7	0.3
J6-56.D	J6-56	2	25.88	17.9	0.1
J6-56.E	J6-56	2	22.72	17.3	0.0
J6-56.F	J6-56	2	19.50	16.7	0.1
J6-56.G	J6-56	2	16.30	15.8	0.1
J6-56.H	J6-56	2	13.69	15.1	0.1
J6-56.I	J6-56	2	10.99	14.2	0.1
J6-56.J	J6-56	2	7.57	14.1	0.1
J6-56.K	J6-56	2	4.59	14.4	0.0

Sample ID	Tooth	Layer	mm from ERJ	$\delta^{18}O$	SD
J6-56.L	J6-56	2	1.95	15.0	0.0
I6-266.A	I6-266	2	36.42	15.0	0.2
I6-266.B	I6-266	2	33.32	14.4	0.0
I6-266.C	I6-266	2	30.25	13.8	0.3
I6-266.D	I6-266	2	27.90	13.5	0.1
I6-266.E	I6-266	2	25.63	13.2	0.1
I6-266.F	I6-266	2	22.57	12.9	0.2
I6-266.G	I6-266	2	19.85	13.0	0.0
I6-266.H	I6-266	2	16.58	13.4	0.2
I6-266.I	I6-266	2	13.59	13.7	0.5
I6-266.J	I6-266	2	10.27	14.2	0.3
I6-266.K	I6-266	2	7.41	14.3	0.2
I6-266.L	I6-266	2	4.21	14.7	0.3
I6-266.M	I6-266	2	1.38	15.1	0.2
F6-441.A	F6-441	2	12.75	14.5	0.2
F6-441.B	F6-441	2	10.78	14.1	0.1
F6-441.C	F6-441	2	8.55	13.7	0.3
F6-441.D	F6-441	2	6.22	13.8	0.1
F6-441.E	F6-441	2	4.57	13.8	0.3
F6-441.F	F6-441	2	2.30	15.0	0.0
F7-14.A	F7-14	2	29.35	17.2	0.3
F7-14.B	F7-14	2	27.68	17.3	0.4
F7-14.C	F7-14	2	24.86	17.2	0.5
F7-14.D	F7-14	2	22.58	17.7	0.2
F7-14.E	F7-14	2	19.74	17.4	0.1
F7-14.F	F7-14	2	17.27	16.8	0.3
F7-14.G	F7-14	2	14.49	16.6	0.2
F7-14.H	F7-14	2	11.91	16.2	0.2
F7-14.I	F7-14	2	8.92	16.1	0.2
F7-14.J	F7-14	2	6.00	16.7	0.4
F7-14.K	F7-14	2	3.99	16.3	0.0
F7-14.L	F7-14	2	1.91	16.8	0.3
F7-294.A	F7-294	2	53.03	17.7	0.1
F7-294.B	F7-294	2	51.60	17.5	0.3
F7-294.C	F7-294	2	50.72	17.1	0.1
F7-294.D	F7-294	2	47.19	16.2	0.1
F7-294.E	F7-294	2	44.28	15.6	0.1
F7-294.F	F7-294	2	41.99	15.1	0.3

Sample ID	Tooth	Layer	mm from ERJ	$\delta^{18}O$	SD
F7-294.G	F7-294	2	39.95	14.7	0.1
F7-294.H	F7-294	2	37.08	14.2	0.2
F7-294.I	F7-294	2	33.97	14.0	0.3
F7-294.J	F7-294	2	31.49	13.8	0.3
F7-294.K	F7-294	2	29.08	13.7	0.2
F7-294.L	F7-294	2	26.53	14.2	0.1
F7-294.M	F7-294	2	24.04	15.0	0.1
F7-294.N	F7-294	2	21.62	15.5	0.2
F7-294.O	F7-294	2	18.89	16.3	0.1
F7-294.P	F7-294	2	15.99	17.7	0.1
F7-294.Q	F7-294	2	12.90	18.1	0.2
F7-294.R	F7-294	2	10.62	18.8	0.3
F7-294.S	F7-294	2	7.65	18.7	0.2
F7-294.T	F7-294	2	5.16	18.1	0.2
F7-294.U	F7-294	2	2.95	17.2	0.5
F7-294.V	F7-294	2	0.70	17.3	0.0
J6-141.A	J6-141	2	30.08	17.1	0.2
J6-141.B	J6-141	2	26.84	16.6	0.2
J6-141.C	J6-141	2	23.18	15.3	0.3
J6-141.D	J6-141	2	19.94	14.3	0.2
J6-141.E	J6-141	2	17.57	13.9	0.2
J6-141.F	J6-141	2	14.22	13.0	0.4
J6-141.G	J6-141	2	11.40	12.9	0.3
J6-141.H	J6-141	2	8.67	12.8	0.3
J6-141.I	J6-141	2	5.91	12.6	0.3
J6-141.J	J6-141	2	2.89	12.8	0.2
I4-2295.A	I4-2295	5A	60.30	16.5	0.1
I4-2295.B	I4-2295	5A	57.20	15.1	0.4
I4-2295.C	I4-2295	5A	54.40	15.2	0.3
I4-2295.D	I4-2295	5A	51.10	15.4	0.4
I4-2295.E	I4-2295	5A	48.20	15.6	0.6
I4-2295.F	I4-2295	5A	45.20	15.8	0.4
I4-2295.G	I4-2295	5A	41.90	16.2	0.5
I4-2295.H	I4-2295	5A	38.80	16.6	0.4
I4-2295.I	I4-2295	5A	35.80	16.7	0.6
I4-2295.J	I4-2295	5A	32.60	17.1	0.3
I4-2295.K	I4-2295	5A	29.40	17.3	0.5
I4-2295.L	I4-2295	5A	26.40	17.7	0.1

Sample ID	Tooth	Layer	mm from ERJ	$\delta^{18}O$	SD
I4-2295.M	I4-2295	5A	22.60	18.1	0.1
I4-2295.N	I4-2295	5A	19.20	17.9	0.2
I4-2295.O	I4-2295	5A	15.80	17.8	0.3
I4-2295.P	I4-2295	5A	12.00	17.3	0.1
I4-2295.Q	I4-2295	5A	9.10	16.8	0.2
I4-2295.R	I4-2295	5A	6.00	16.1	0.1
I4-2295.S	I4-2295	5A	3.00	15.8	0.2
I3-2032.A	I3-2032	5A	32.80	15.5	0.2
I3-2032.B	I3-2032	5A	31.30	15.8	0.4
I3-2032.C	I3-2032	5A	28.20	16.3	0.4
I3-2032.D	I3-2032	5A	25.20	16.1	0.3
I3-2032.E	I3-2032	5A	21.70	16.9	0.1
I3-2032.F	I3-2032	5A	18.80	17.6	0.3
I3-2032.G	I3-2032	5A	15.60	17.9	0.0
I3-2032.H	I3-2032	5A	12.30	18.0	0.3
I3-2032.I	I3-2032	5A	9.40	17.7	0.3
I3-2032.J	I3-2032	5A	6.10	16.8	0.4
I3-2032.K	I3-2032	5A	3.50	16.6	0.3
I3-2032.L	I3-2032	5A	0.70	16.4	0.3

Supplementary Table S5: Strontium isotope values of a subset of sequential tooth enamel samples used to assess *Bos/Bison* migratory behavior.

Sample ID	Layer	mm from ERJ	$^{87/86}Sr$	Concentration (ppm)
F7-294.E	2	44.28	0.7104	138
F7-294.L	2	26.53	0.7102	118
F7-14.C	2	24.86	0.7109	245
J6-56.C	2	28.87	0.7101	167
J6-56.I	2	10.99	0.7100	166
F7-14.F	2	17.27	0.7109	128
F7-14.I	2	8.92	0.7108	146
J4-67.C	5A	41.93	0.7106	115
J4-67.J	5A	20.60	0.7104	113
I4-792.G	5B	22.27	0.7101	84
I4-792.M	5B	4.40	0.7105	82
I4-616.D	5B	26.35	0.7104	85
I4-616.J	5B	6.99	0.7102	110
I4-785.C	5B	43.84	0.7107	109
I4-785.L	5B	16.35	0.7101	101

Supplementary Table S6: Carbon and nitrogen isotope results for *Bos/Bison* bone collagen samples with standard deviation of replicate measurements.

Sample ID	Layer	Taxon	Skeletal element	δ^{13}C	SD	δ^{15}N	SD	% C	% N	C/N
G6-600	1	Bos/Bison	R Femur	-20.3	0.0	5.9	0.1	27.2	9.6	3.3
F6-523	1	Bos/Bison	MT	-20.4	0.0	5.2	0.0	36.4	12.9	3.3
H6-366	1	Bos/Bison	Tibia	-20.9	NA	7.0	NA	36.1	12.8	3.3
G6-601	1	Bos/Bison	R Tibia	-20.8	0.1	5.2	0.0	41.9	14.3	3.4
I6-359	2	Bos/Bison	L Tibia	-20.3	0.1	5.4	0.0	36.2	12.5	3.4
I6-373	2	Bos/Bison	L Tibia	-20.2	0.1	6.1	0.2	23.4	8.3	3.3
J6-175	2	Bos/Bison	L Tibia	-20.4	0.1	6.6	0.0	37.4	13.0	3.4
I6-252	2	Bos/Bison	L Tibia	-20.0	0.0	5.5	0.1	31.0	10.9	3.3
G6-184	3	Bos/Bison	R Tibia	-20.0	NA	5.8	0.0	37.5	13.8	3.2
G6-154	3	Bos/Bison	MT	-20.4	0.1	7.0	0.2	11.7	4.0	3.4
F7-107	3	Bos/Bison	MC	-20.4	0.1	7.6	0.0	39.5	14.1	3.3
K6-212	4	Bos/Bison	Cranial	-20.6	0.0	4.9	0.0	27.7	9.5	3.4
L6-216	4	Bos/Bison	Rib	-21.0	0.1	5.4	0.1	38.8	13.7	3.3
K6-173	4	Bos/Bison	R Tibia	-20.4	0.0	5.0	0.0	38.7	13.3	3.4
I5-68	4	Bos/Bison	MT	-20.3	0.1	5.8	0.0	39.9	14.1	3.3
L6-141	5A	Bos/Bison	R Tibia	-20.7	0.0	5.6	0.1	32.0	10.9	3.4
I4-826	5A	Bos/Bison	Mandible	-20.6	0.0	5.8	0.0	33.4	11.6	3.4
I4-823	5A	Bos/Bison	Scapula	-21.0	0.0	6.4	0.1	33.4	11.5	3.4
I4-873	5A	Bos/Bison	Femur	-20.8	NA	5.7	NA	40.7	14.1	3.4
I4-637	5B	Bos/Bison	Humerus	-20.6	0.2	6.3	0.0	33.0	11.2	3.5
I4-615	5B	Bos/Bison	MT	-20.6	0.2	6.5	0.1	19.6	6.8	3.4
I4-602	5B	Bos/Bison	MT	-20.6	NA	7.1	NA	23.8	8.6	3.2
I4-605	5B	Bos/Bison	L Tibia	-20.8	0.0	6.0	0.1	38.9	13.7	3.3

Supplementary Table S7: Cross-comparison of all sampled *Bos/Bison* teeth from Layer 5B to determine which teeth could originate from the same individual. Criteria of tooth position, tooth wear and (where applicable) oxygen stable isotope data were used to determine if teeth could be possible matches for the same individual (indicated as 'possible match') or if origin from the same individual can be excluded (indicated as 'no match'). Decisive criteria for excluding matches are indicated in parentheses.

Layer 5B	I4-792	I4-785	I4-616
		no match (tooth wear)	no match (isotopes)
			no match (tooth position)

Supplementary Table S8: Cross-comparison of all sampled *Bos/Bison* teeth from Layer 5A to determine which teeth could originate from the same individual. Criteria of tooth position, tooth wear and (where applicable) oxygen stable isotope data were used to determine if teeth could be possible matches for the same individual (indicated as 'possible match') or if origin from the same individual can be excluded (indicated as 'no match'). Decisive criteria for excluding matches are indicated in parentheses.

Layer 5A	J4-67	I3-978	I4-2295	I3-2032
		no match (isotopes)	no match (tooth wear)	no match (tooth wear, isotopes)
			no match (tooth wear)	no match (tooth position)
				no match (tooth wear)

Supplementary Table S9: Cross-comparison of all sampled *Bos/Bison* teeth from Layer 2 to determine which teeth could originate from the same individual. Criteria of tooth position, tooth wear and (where applicable) oxygen stable isotope data were used to determine if teeth could be possible matches for the same individual (indicated as ‘possible match’) or if origin from the same individual can be excluded (indicated as ‘no match’). Decisive criteria for excluding matches are indicated in parentheses. As individual F7-14 did not yield a sinusoidal oxygen isotope time series, it does not isotopically match any other teeth and was not used for seasonal climate reconstruction. We have thus also excluded it from comparison here.

Layer 2	J6-56	J6-141	I6-266	F6-441	F7-294
		no match (tooth wear)	no match (tooth position)	possible match	no match (isotopes, tooth wear)
			no match (tooth wear)	no match (isotopes)	no match (isotopes, tooth wear)
				no match (isotopes, tooth wear)	no match (tooth wear)
					no match (tooth wear)
